# Synthesis of Triamino Acid Building Blocks with Different Lipophilicities

**DOI:** 10.1371/journal.pone.0124046

**Published:** 2015-04-14

**Authors:** Jyotirmoy Maity, Dmytro Honcharenko, Roger Strömberg

**Affiliations:** Department of Biosciences and Nutrition, Novum, Karolinska Institute (KI), Stockholm, Sweden; Louisiana State University Health Sciences Center, UNITED STATES

## Abstract

To obtain different amino acids with varying lipophilicity and that can carry up to three positive charges we have developed a number of new triamino acid building blocks. One set of building blocks was achieved by aminoethyl extension, *via* reductive amination, of the side chain of ortnithine, diaminopropanoic and diaminobutanoic acid. A second set of triamino acids with the aminoethyl extension having hydrocarbon side chains was synthesized from diaminobutanoic acid. The aldehydes needed for the extension by reductive amination were synthesized from the corresponding Fmoc-L-2-amino fatty acids in two steps. Reductive amination of these compounds with Boc-L-Dab-OH gave the C4-C8 alkyl-branched triamino acids. All triamino acids were subsequently Boc-protected at the formed secondary amine to make the monomers appropriate for the N-terminus position when performing Fmoc-based solid-phase peptide synthesis.

## Introduction

Lipophilicity has immense importance for pharmacological properties. Drug molecules are required to have lipophilic properties to accomplish a desired pharmacokinetic profile [1]. Oligonucleotides and peptides having inadequate affinity with the lipid bilayer of plasma membranes are conjugated with lipophilic parts to enhance their cellular uptake [2]. Antisense oligonucleotides were conjugated to cholesterol and bile acids to enhance lipophilicity and to improve liver specific drug targeting and hepatocellular uptake efficiency [3]. Arginine-based double-tailed lipid-peptide conjugates with a positive charge were synthesized as a potent nucleic acid transporter [4]. Cationic lipid-mediated nucleic acids delivery has emerged as a positive move towards delivering genes into mammalian cells. Various cationic liposomes have been used for gene delivery to mammalian cells *in vitro* and *in vivo* [5]. Arginine-rich peptide sequence with peptide amphiphiles at its N-terminus had shown spontaneous assembly formation of various nanostructures in aqueous solution. Micelles of these peptides were loaded with the anti-tumor drug doxorubicin and delivery of the drug into HeLa cells was observed [6]. Addition of a lipid tail at the N-terminus of the antimicrobial peptide tridecaptin A1 was found to enhance the biological activity. Some simpler analogues were also found to show antimicrobial activity against Gram-negative bacteria [7]. Di- or tri-peptide analogues, when lipidated with a C_12-18_ lipid at the C-terminus of the peptides, exhibited enhanced antimicrobial activity compared to their basic di- or tri-peptides [8].

Lysine is one of the naturally occurring amino acids that have an aliphatic side chain with a primary amine functionality at the terminus. Besides the high level of safe supplemental intake of L-lysine [9], it has been used therapeutically to restrain herpes simplex [10] and found to be effective in the treatment of stress-related intestinal disorders [11]. A triamino acid, 4-L-azalysine and its analogues have been found to retain engaging pharmacological properties. It showed inhibitory activity towards the growth of *E*. *Coli*. 9723 and a broad range of lactic acid bacteria [12]. It has been found to be efficient as a metabolic inhibitor of arylesterase [13]. Triamino acids at the N-terminus part of peptoid ligands targeting the α-helical conformation of the amyloid-β peptide (Aβ) related to Alzheimers disease have also been shown to improve their antineurotoxicity [14]. The biological significance and potential medicinal importance of triamino acids and amino acids/peptides with hydrocarbon tails encouraged us to extend the arsenal of amino acids with such functionalities by synthesis of some new triamino acid building blocks with as well as without a hydrocarbon branching.

**Fig 1:**
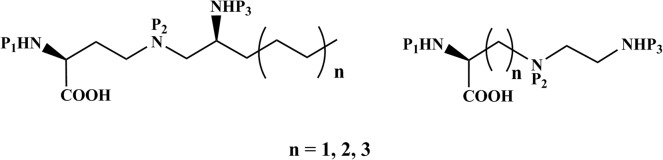
Schematic structures of the triamino acid building blocks. (P_1_-P_3_ = protecting groups).

We here describe the strategy for synthesis of two sets of triamino acids. One set is varied with respect to distance between the alpha-carbon and the secondary amine of the side chain and the other set of compounds have aliphatic hydrocarbon tails of different length adjacent to the terminus amino functionality of the side chain (Fig 1). When these amino acid monomers are incorporated at the N-terminus of any potential peptides/peptoids, the amino groups will be partially/fully protonated depending on pH of the solution and the pKa value of the respective amines, and together with the hydrocarbon chain of the branched derivatives this creates a cationic/hydrophobic microenvironment at the N-terminus of the peptide/peptoid. In addition to this, the hydrocarbon chain of the branched derivatives introduces additional lipophilic character, creating a cationic environment along with lipophilicity at the N-terminus of the peptides/peptoids. Protecting groups of these triamino acids have been manipulated in such a way that the final monomers would be suitable for their incorporation at the N-terminus end of a peptide/peptoid sequence by the Fmoc-strategy through solid-phase peptide synthesis, while still enabling further functionalization of the side chain.

## Results and Discussion

We chalked out synthetic schemes for the target molecules largely from commercially available, stereochemically pure and suitably protected starting materials. We found that reductive amination reaction between *N* ^α^-Fmoc-protected amino aldehydes and side chain primary amine of the *N* ^α^-Boc-protected diamino carboxylic acids would provide us the basic structural moiety of the target molecules. Originating the functionality of the aldehydes to carboxylic acids gave us a plan to start the synthetic pathway from the corresponding stereochemically pure *N*-protected *N* ^α^-amino-carboxylic acids.

The synthetic route chosen is based on that we wished to protect the terminus N7 amine position with a 9-fluorenylmethyloxycarbonyl (Fmoc) group, for possible further extension at this position. Accordingly we wanted the other primary and secondary amino functionalities N2 and N5 respectively, to be protected with a *tert*-butyloxycarbonyl (Boc) group so that these can be simultaneously removed upon final deprotection when performing Fmoc-based solid-phase synthesis. For incorporation into a peptide chain the Boc-protected aldehyde and an N2-Fmoc protection could instead be used. Conversion of Fmoc-protected amino acids into their corresponding Fmoc-protected amino aldehydes has been accomplished by two major approaches. One is through reduction of the acids into alcohols, followed by oxidation and a second is by the synthesis of Weinreb amides, followed by reduction [15–20]. Another noticeable approach is *via* synthesis of amino esters, where acids were converted into their corresponding ethyl esters by treatment with ethanol and sulphuric acid, followed by reduction with diisobutylaluminium hydride (DIBAL) under inert condition [21]. Synthetic procedures for the synthesis of these types of chiral aldehyde building blocks are also available in literature [22].

**Fig 2:**
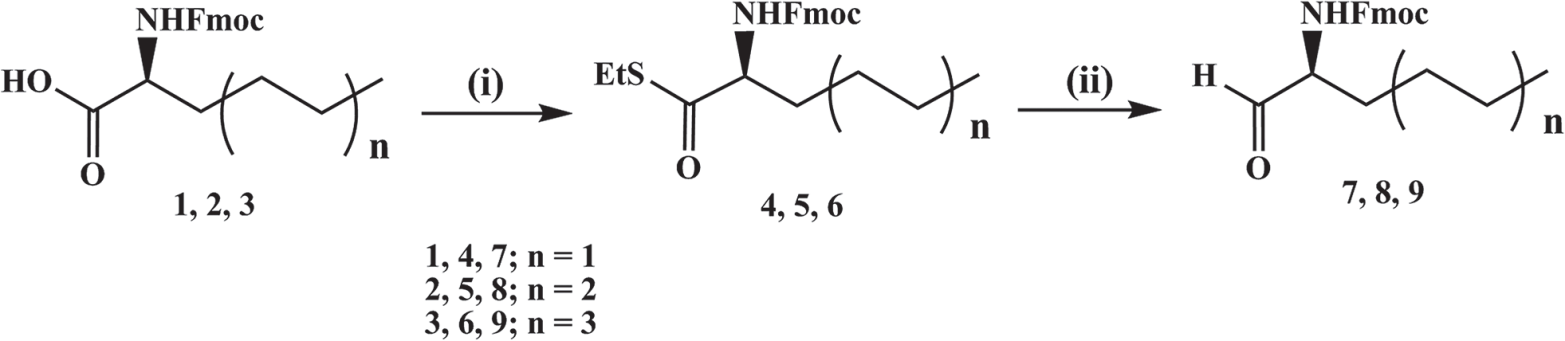
Synthesis of alkyl branched amino aldehydes. (i) EtSH, DCC, DMAP, dichloromethane at rt, 2 h (ii) Triethylsilane, 10% Pd/C, acetone at rt, 2 h.

Our strategy for the synthesis of amino aldehydes was through the synthesis of thioesters of the available amino acids [23–25], followed by reduction at neutral condition. We commenced our synthetic pathway with commercially available chiral (*S*)-*N*-Fmoc-2-amino-2-alkylacetic acids (**1**, **2** and **3**; Fig 2). The synthetic route was chosen so that the chirality of these molecules would be intact. Ethyl thioesters were readily derived from the corresponding carboxylic acids using 1.1 equiv. of *N*,*N'*-dicyclohexylcarbodiimide (DCC) in dichloromethane at room temperature [26]. Use of 3–10 mol% of 4-(*N*,*N*-dimethylamino)pyridine (DMAP) for this type of esterification reaction has been suggested to accelerate the rate of the reaction between carboxylic acids and thiols, and also to suppress side product formation [26]. The desired products were obtained in good yields when we performed these reactions at room temperature using 0.25 equiv. of DMAP. Reactions were smooth and complete conversion occurred in two hours. A facile work up procedure followed by purification gave us the corresponding *N*-Fmoc-2-amino-ethyl thioesters **4**, **5** and **6** respectively.

α-Amino aldehydes are widely used chiral synthons in organic chemistry but they have a tendency to racemize under acidic or basic conditions and also during chromatographic purification over silica gel. This directed us towards milder conditions for the reduction reaction with a simple work up procedure. We dissolved the ethanethiol esters in acetone at room temperature and treated them with triethylsilane in presence of catalytic 10% Pd/C to convert the thioester functionality into an aldehyde [27]. The reactions were allowed to proceed for two hours. A simple work up protocol and purification of the crude reaction mixture by column chromatography gave the *N*-Fmoc-2-amino-aldehydes **7**, **8** and **9** respectively (Fig 2).

**Fig 3:**
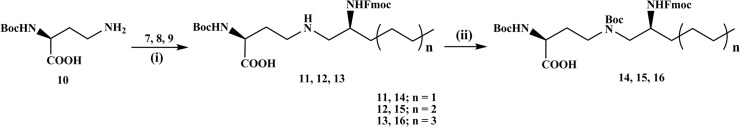
Synthesis of triamino acid building blocks with varied lipophilic tails. (i) NaBH_3_CN, 1% AcOH in methanol, rt, 18 h (ii) (Boc)_2_O, water:dioxane (v/v, 1:1), 10% aq. soln. of Na_2_CO_3_, rt, 18 h.

After synthesis of the chiral aldehydes our next task was to attempt the key step of our synthetic pathway, *i*.*e*. the reductive amination reaction of these chiral aldehydes with a chiral diamino carboxylic acid. Reductive amination reaction is a multipurpose and convenient method for the preparation of amines in organic synthesis [28]. A variety of organocatalysts, complexes of transition metals or boron, tin and silicon reagents are available for this reaction. We selected sodium cyanoborohydride (NaBH_3_CN) as a suitable reagent due to its earlier applications for reductive alkylation reaction in amino acid chemistry and/ or peptide chemistry [29–33]. *N* ^2^-Boc-2,4-diamino-butanoic acid (**10**) was subjected to a reductive amination reaction with aldehyde **7** at room temperature in a solvent mixture of acetic acid/ methanol (1:99, v/v) where NaBH_3_CN was used as the catalyst for the reaction (Fig 3). The reaction mixture was stirred for 18 h at room temperature for the production of the desired compound (with retained chirality at the C-2 and C-7 centre). The progress of the reaction was monitored with TLC and, after a work up process, the crude reaction mixture was purified by silica gel column chromatography to give compound **11** in good yield. The triamino acid was then further protected with Boc at the secondary amine by treatment with Boc-anhydride in a solvent mixture of water and dioxane (1:1, v/v), containing 10% Na_2_CO_3_ aqueous solution. This reaction afforded the final product (2*S*,2'*S*)-*N* ^2^,*N* ^4^-bis(*tert*-butoxycarbonyl)-*N* ^4^-[*N* ^2'^-(9-fluorenylmethyloxycarbonyl)-2'-aminohexyl]-2,4-diaminobutanoic acid (**14**).

Similar procedures were used for synthesis of triamino acids branched with longer (C6 and C8) alkyl chains. The diamino acid **10** was treated with the chiral aldehydes **8** and **9** in presence of NaBH_3_CN to produce the triamino carboxylic acids **12** and **13**, respectively. Subsequent Boc protection gave the monomers **15** and **16**, respectively (Fig 3).

Three different strategies for the synthesis of 4-L-azalysine have been described. The first strategy involved L-serine as the starting material which was converted into methyl (*S*)-oxazolidine-4-carboxylate in three steps. In next five steps, it was converted into the end product *via* Garner’s aldehyde. A second strategy involved the use of L-asparagine that was converted into *N*-protected 2,3-diaminopropionic ester, followed by reductive amination with *N*-Boc-2-aminoacetaldehyde. Strategic manipulation of protecting groups produced the final desired product in another four steps [34,35]. The third strategy was a solid-phase dependent procedure to synthesize di- or polycationic amino acid building blocks. In this protocol, protected aziridine-2-methanol was loaded onto a trityl bromide resin, followed by ring opening with a variety of primary amines. After detachment of the product from solid support, the primary alcohol was converted into a carboxylic acid [36]. For synthesis of the triamino acids with different distances between the alpha-carbon and the secondary amine of the side chain we opted for a short route starting with the respective amino acids, ortnithine, diaminopropanoic and diaminobutanoic acid. We have reported on synthesis of a couple of these triamino acids but with a different choice of protection scheme [14, 37]. As with the branched derivative we wished to have building blocks for termination of a peptide/peptoid with possibility for extension on the side chain and therefore an Fmoc-protection on the side chain amino group and the *N* ^2^-Boc protection was used on the diamino acid.

**Fig 4:**
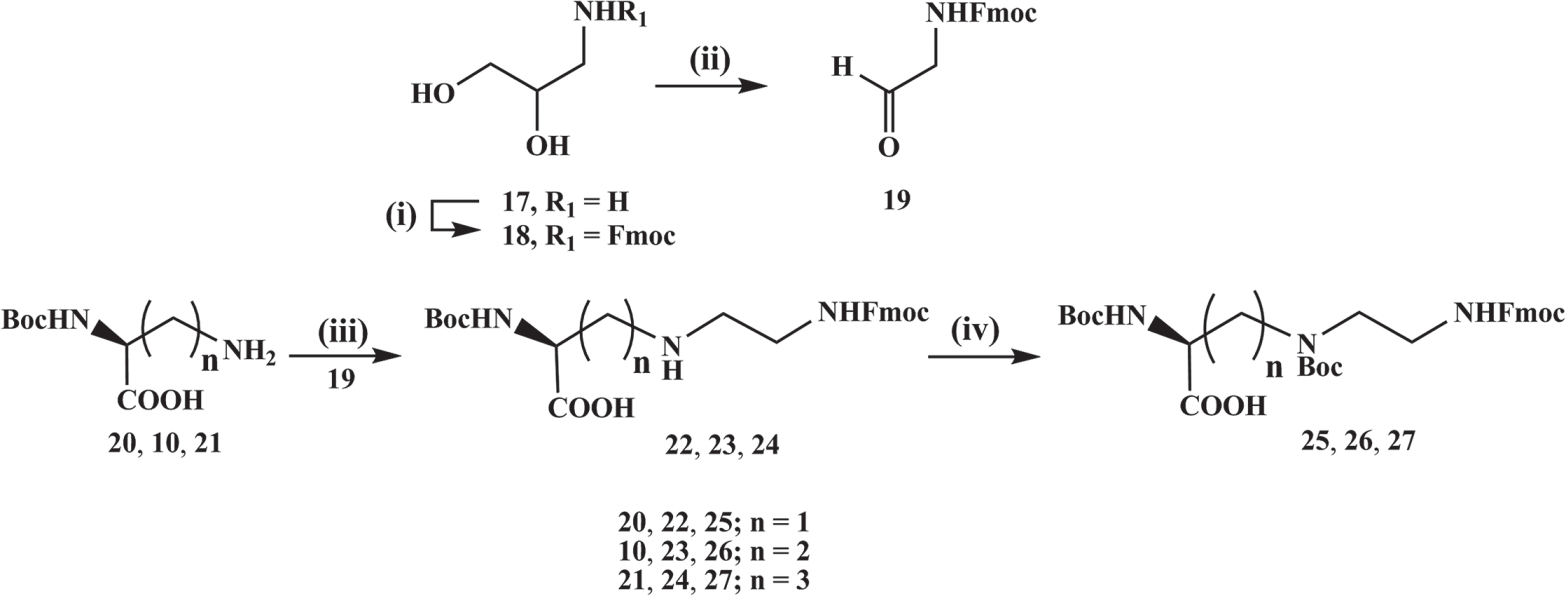
Synthesis of triamino acid building blocks with different distances between the α-amino group and the secondary amine. (i) Fmoc-OSu, MeOH/ pyridine, rt, 18 h (ii) NaIO_4_, THF, rt, 8 h (iii) NaBH_3_CN, 1% AcOH in methanol, rt, 18 h (iv) (Boc)_2_O, water:dioxane (v/v, 1:1), 10% aq. Na_2_CO_3_, rt, 18 h.

The synthetic pathway is similar to that for the compounds with aliphatic branching except that *N*-Fmoc-glycinal (**19**) was used instead of the branched aminoaldehydes. The *N*-Fmoc-glycinal was synthesized from of the inexpensive starting material 3-aminopropane-1,2-diol (**17**) which was Fmoc protected to form **18** and then oxidatively cleaved with periodate to give **19** (Fig 4) [38].

Reductive amination reaction with compound **19** and the respective *N* ^α^-Boc-diamino acids **10**, **20**, **21** in acetic acid/methanol (1:99, v/v) using NaBH_3_CN at room temperature resulted in the triamino acid derivatives **22–24**. After work up and purification by column chromatography Boc protection on the secondary amine was achieved by treatment with (Boc)_2_O to afford the products (*S*)-*N* ^2^,*N* ^3^-bis-*tert*-butoxycarbonyl-*N* ^3^-[*N*-(9-fluorenylmethyloxycarbonyl)-2-aminoethyl]-2,3-diaminopropanic acid (**25**), (*S*)-*N* ^2^,*N* ^4^-bis-*tert*-butoxycarbonyl-*N* ^4^-[*N*-(9-fluorenylmethyloxycarbonyl)-2-aminoethyl]-2,4-diaminobutanoic acid (**26**) and (*S*)-*N* ^2^,*N* ^5^-bis-*tert*-butoxycarbonyl-*N* ^5^-[*N*-(9-fluorenylmethyloxycarbonyl)-2-aminoethyl]-2,3-diaminopentanoic acid (**27**).

## Conclusion

We have revealed a facile synthetic procedure for the preparation of suitably protected triamino acids in decent to good yields. Thus an extension of the available arsenal of triamino acids building blocks with varying lipophilicity and that can carry up to three positive charges is provided. Starting from *N*-Fmoc-2-alkyl amino acids (**1–3**) with varied chain length of the alkyl group, we converted them into the corresponding aminoaldehydes (**7–9**) in two steps. These aldehydes were protected and suitable for reductive amination reaction with the protected diamino acid *N* ^α^-Boc-L-Dab (**10**). The resulting alkyl branched triamino acids were Boc-protected to obtain the final monomers (**14–16**). In addition another series of triamino acids with different distances between the alpha-carbon and the secondary amine of the side chain (**25–27**) were made by reductive amination with *N*-Fmoc-glycinal (**19**) and a series of *N* ^α^-Boc diamino acids (**20**, **10**, **21**). This facile and variable procedure provided novel amino acids with hydrocarbon branching of the aminoethyl extension and convenient synthesis of triamino acids with different distances between the alpha-carbon and the secondary amine. The final monomers were suitably protected for their incorporation at the N-terminus of a peptide/peptoid sequence by Fmoc-based solid-phase synthesis while enabling further functionalization of the side chain when still attached to the support.

## Experimental Section

Melting points of the compounds were recorded using Büchi Melting Point apparatus (B-545) and were uncorrected. Reactions under anhydrous conditions were carried out under a nitrogen atmosphere. Column chromatography was performed with silica gel 60 (particle size 0.040–0.063 mm, 230–400 mesh, Aldrich) and analytical grade solvents. Thin layer chromatography (TLC) was conducted on glass plates coated with silica gel 60 F_254_, obtained from Merck. TLC plates were visualized by UV light (254 or 360 nm) and/or by staining with I_2_ by keeping the plates in an iodine chamber. ^1^H NMR (400 MHz) and ^13^C NMR (100 MHz) spectra were recorded on a Bruker DRX-400 in CDCl_3_/CD_3_OD at 25°C. Chemical shifts are reported in ppm relative to TMS (Me_4_Si as internal standard, *δ* = 0 ppm) or the deuterated solvent as the internal standard for ^1^H and ^13^C NMR. Coupling constants (*J* values) are given in Hz. The following abbreviations are used in connection with NMR spectra: s = singlet, br s = broad singlet, d = doublet, dd = double doublet, t = triplet, q = quartet and m = multiplet. Structural assignments are based on DEPT-135, COSY and HMQC where applicable. HRMS (ESI-TOF) was recorded on a Micromass LCT either in positive ion mode or in negative ion mode. Specific rotation measurements were performed using an Autopol IV Automatic Polarimeter (Rudolph Research Analytical). RP-HPLC analysis of final building blocks is reported in S1 Supporting Information. THF, CH_2_Cl_2_, toluene, hexane, and Et_2_O were dried by standard procedures and stored over molecular sieves (4 Å). *N*-(9-Fluorenylmethyloxycarbonyl)-2-aminoacetaldehyde (**19**) [38], **(** *S*)-*N*-(9-fluorenylmethoxycarbonyl)-2-aminohexanal (**7**) [25] and **(** *S*)-*N* ^2^-*tert*-butoxycarbonyl-*N* ^5^-[*N*-(9-fluorenylmethyloxycarbonyl)-2-aminoethyl]-2,5-diaminopentanoic acid (**24**) [38] were synthesized as reported (see S1 Supporting Information). All other solvents and chemicals were of reagent grade and used without further purification. 2-Alkyl amino acids **1**, **2** and **3** were purchased from Watanabe Chemical Industries, Ltd., Japan and Boc-protected amino acids **10**, **20** and **21** were purchased from IRIS Biotech, Germany. Other reagents were obtained from common commercial sources and used as received.

### General procedure for synthesis of ethylthio esters (4, 5 and 6)

To a solution of Fmoc-amino acids (**1–3**, 1.3 mmol) in anhydrous dichloromethane (DCM, 20 mL) at rt, ethanethiol (5 mmol) was added dropwise, followed by addition of solid DCC (1.6 mmol) and (DMAP, 0.25 mmol) under inert atmosphere. The reaction mixture was stirred for 2 h at rt. Progress of the reaction was monitored by TLC. Upon complete conversion of the starting material into product, water (20 mL) was added into the reaction mixture and the layers were separated. The organic layer was collected and washed with brine (2 x 10 mL), dried over Na_2_SO_4_ and concentrated to dryness under reduced pressure to get the crude materials. Pure products (**4–6**) were obtained by eluting the crude through a short column of silica gel.

#### (*S*)-S-Ethyl *N*-(9-fluorenylmethoxycarbonyl)-2-aminohexanthioate (4)

Compound **4** was purified by flash column chromatography using 0 to 25% ethyl acetate (EtOAc) in hexane as eluent to afford a white amorphous solid (0.36 g, 71%); m.p. 116–117°C. R_*f*_ = 0.54 (EtOAc/hexane, 1:5, v/v). ^1^H NMR (400 MHz, CDCl_3_): *δ* = 7.69 (d, *J* = 7.6 Hz, 2 H, Ar-H), 7.54 (t, *J* = 7.2 Hz, 2 H, Ar-H), 7.33 (t, *J* = 7.6 Hz, 2 H, Ar-H), 7.24 (t, *J* = 7.6 Hz, 2 H, Ar-H), 5.13 (d, *J* = 8.0 Hz, 1 H, NH), 4.44–4.40 (m, 1 H, NCOOC*H*_2a_CH), 4.36–4.30 (m, 2 H, NCOOC*H*_2b_CH, 2-CH), 4.17 (t, *J* = 6.8 Hz, 1 H, NCOOCH_2_C*H*), 2.81 (q, *J* = 7.2 Hz, 2 H, COSC*H*_2_CH_3_), 1.85–1.80 (m, 1 H, 3-CH_2a_), 1.57–1.51 (m, 1 H, 3-CH_2b_), 1.30–1.23 (m, 4 H, 4-CH_2_, 5-CH_2_), 1.18 (t, *J* = 7.2 Hz, 3 H, COSCH_2_C*H*_3_), 0.83 (t, *J* = 6.8 Hz, 3 H, 6-CH_3_) ppm. ^13^C NMR (100 MHz, CDCl_3_): *δ* = 201.1 (COS), 156.0 (NCOO), 144.1, 143.9, 141.5 (C-Ar), 127.9, 127.2, 125.2, 120.1 (CH-Ar), 67.2 (NCOO*C*H_2_CH), 61.1 (C-2), 47.4 (NCOOCH_2_ *C*H), 32.8 (C-3), 27.5 (C-4), 23.4 (S*C*H_2_CH_3_), 22.4 (C-5), 14.6 (SCH_2_ *C*H_3_), 14.0 (C-6) ppm. MS-ESI (*m/z*): calcd. for C_23_H_28_NO_3_S [M+H]^+^ 398.1784; found 398.1779.

#### (*S*)-S-Ethyl *N*-(9-fluorenylmethoxycarbonyl)-2-aminooctanthioate (5)

Compound **5** was purified by flash column chromatography using 0 to 25% EtOAc in hexane as eluent to afford a white amorphous solid (0.33 g; 86%); m.p. 96–99°C. R_*f*_ = 0.56 (EtOAc/hexane, 1:5, v/v). ^1^H NMR (400 MHz, CDCl_3_): *δ* = 7.70 (d, *J* = 7.6 Hz, 2 H, Ar-H), 7.55 (t, *J* = 7.2 Hz, 2 H, Ar-H), 7.33 (t, *J* = 7.6 Hz, 2 H, Ar-H), 7.25 (t, *J* = 7.6 Hz, 2 H, Ar-H), 5.11 (d, *J* = 8.4 Hz, 1 H, NH), 4.44–4.40 (m, 1 H, NCOOC*H*_2a_CH), 4.36–4.30 (m, 2 H, NCOOC*H*_2b_CH, 2-CH), 4.17 (t, *J* = 6.8 Hz, 1 H, NCOOCH_2_C*H*), 2.82 (q, *J* = 7.2 Hz, 2 H, COSC*H*_2_CH_3_), 1.84–1.78 (m, 1 H, 3-CH_2a_), 1.57–1.49 (m, 1 H, 3-CH_2b_), 1.26–1.16 (m, 11 H, 4-CH_2_, 5-CH_2_, 6-CH_2_, 7-CH_2_, COSCH_2_C*H*_3_), 0.81 (t, *J* = 6.8 Hz, 3 H, 8-CH_3_) ppm. ^13^C NMR (100 MHz, CDCl_3_): *δ* = 201.1 (COS), 156.0 (NCOO), 144.1, 143.9, 141.5 (C-Ar), 127.9, 127.2, 125.2, 120.1 (CH-Ar), 67.2 (NCOO*C*H_2_CH), 61.1 (C-2), 47.4 (NCOOCH_2_ *C*H), 33.1 (C-3), 31.7 (C-4), 29.0 (C-5), 25.3 (C-6), 23.4 (S*C*H_2_CH_3_), 22.7 (C-7), 14.7 (SCH_2_ *C*H_3_), 14.2 (C-8) ppm. MS-ESI (*m/z*): calcd. for C_25_H_32_NO_3_S [M+H]^+^ 426.2103; found 426.2101.

#### (*S*)-S-Ethyl *N*-(9-fluorenylmethoxycarbonyl)-2-aminodecan-thioate (6)

Compound **6** was purified by flash column chromatography using 0 to 20% EtOAc in hexane as eluent to afford a white amorphous solid (0.39 g; 87%); m.p. 92–93°C. R_*f*_ = 0.59 (EtOAc/hexane, 1:5, v/v). ^1^H NMR (400 MHz, CDCl_3_): *δ* = 7.69 (d, *J* = 7.6 Hz, 2 H, Ar-H), 7.54 (t, *J* = 7.2 Hz, 2 H, Ar-H), 7.33 (t, *J* = 7.6 Hz, 2 H, Ar-H), 7.24 (t, *J* = 7.6 Hz, 2 H, Ar-H), 5.13 (d, *J* = 8.4 Hz, 1 H, NH), 4.44–4.39 (m, 1 H, NCOOC*H*_2a_CH), 4.36–4.30 (m, 2 H, NCOOC*H*_2b_CH, 2-CH), 4.17 (t, *J* = 6.8 Hz, 1 H, NCOOCH_2_C*H*), 2.81 (q, *J* = 7.2 Hz, 2 H, COSC*H*_2_CH_3_), 1.84–1.77 (m, 1 H, 3-CH_2a_), 1.56–1.50 (m, 1 H, 3-CH_2b_), 1.26–1.16 (m, 15 H, 4-CH_2_, 5-CH_2_, 6-CH_2_, 7-CH_2_, 8-CH_2_, 9-CH_2_, COSCH_2_C*H*_3_), 0.80 (t, *J* = 6.8 Hz, 3 H, 10-CH_3_) ppm. ^13^C NMR (100 MHz, CDCl_3_): *δ* = 201.1 (COS), 155.9 (NCOO), 144.0, 143.9, 141.5 (C-Ar), 127.9, 127.2, 125.2, 120.1 (CH-Ar), 67.2 (NCOO*C*H_2_CH), 61.1 (C-2), 47.4 (NCOOCH_2_ *C*H), 33.1 (C-3), 32.0 (C-4), 29.5, 29.3, 25.4 (C-5, C-6, C-7, C-8), 23.4 (S*C*H_2_CH_3_), 22.8 (C-9), 14.6 (C-10), 14.2 (SCH_2_ *C*H_3_) ppm. MS-ESI (*m/z*): calcd. for C_27_H_36_NO_3_S [M+H]^+^ 454.2416; found 454.2413.

### General procedure for synthesis of aliphatic amino aldehydes (7, 8 and 9)

Ethylthio esters (**4**–**6**, 0.35 mmol) were dissolved in dry acetone (6 mL) under inert atmosphere. 10% Pd/C was added to the solution followed by addition of triethylsilane (0.56 mmol) whereupon the mixture was stirred at rt. Progress of the reaction was monitored by TLC. After 2 h, the reaction was stopped by passing it through a short pad of celite and washed with acetone (3 x 6 mL). The combined organic layers was evaporated to dryness under reduced pressure and dissolved in ethylacetate (15 mL). After washing the organic layer with brine (2 x 8 mL) it was dried over Na_2_SO_4_ and concentrated under reduced pressure to get the crude products. Subsequent purification by silica gel column chromatography yielded compounds **7**–**9**.

#### (*S*)-*N*-(9-Fluorenylmethoxycarbonyl)-2-aminooctanal (8)

Compound **8** was purified by flash column chromatography using 0 to 60% EtOAc in hexane as eluent to afford a white amorphous solid (0.26 g; 92%); m.p. 75–76°C. R_*f*_ = 0.28 (EtOAc/hexane, 1:5, v/v). ^1^H NMR (400 MHz, CDCl_3_): *δ* = 9.59 (s, 1 H, CHO), 7.77 (d, *J* = 7.6 Hz, 2 H, Ar-H), 7.60 (d, *J* = 7.2 Hz, 2 H, Ar-H), 7.40 (t, *J* = 7.2 Hz, 2 H, Ar-H), 7.32 (t, *J* = 7.6 Hz, 2 H, Ar-H), 5.30 (d, *J* = 6.4 Hz, 1 H, NH), 4.43 (d, *J* = 6.8 Hz, 2 H, NCOOC*H*_2_CH), 4.34–4.29 (m, 1 H, 2-CH), 1.94–1.89 (m, 1 H, NCOOCH_2_C*H*), 1.66–1.58 (m, 1 H, 3-CH_2a_), 1.33–1.26 (m, 9 H, 3-CH_2b_, 4-CH_2_, 5-CH_2_, 6-CH_2_, 7-CH_2_), 0.87 (t, *J* = 7.2 Hz, 3 H, 8-CH_3_) ppm. ^13^C NMR (100 MHz, CDCl_3_): *δ* = 199.3 (CHO), 156.3 (NCOO), 143.8, 141.4 (C-Ar), 127.7, 127.1, 125.0, 120.0 (CH-Ar), 67.0 (NCOO*C*H_2_CH), 60.3 (C-2), 47.2 (NCOOCH_2_ *C*H), 31.5 (C-3), 29.2 (C-4), 29.0 (C-5), 25.0 (C-6), 22.5 (C-7), 14.0 (C-8) ppm. MS-ESI (*m/z*): calcd. for C_23_H_28_NO_3_ [M+H]^+^ 366.2064; found 366.2069.

#### (*S*)-*N*-(9-Fluorenylmethoxycarbonyl)-2-aminodecanal (9)

Compound **9** was purified by flash column chromatography using 0 to 80% EtOAc in hexane as eluent to afford a white amorphous solid (0.36 g; 84%); m.p. 69–70°C. R_*f*_ = 0.35 (EtOAc/hexane, 1:5, v/v). ^1^H NMR (400 MHz, CDCl_3_): *δ* = 9.58 (s, 1 H, CHO), 7.77 (d, *J* = 7.6 Hz, 2 H, Ar-H), 7.60 (d, *J* = 7.6 Hz, 2 H, Ar-H), 7.40 (t, *J* = 7.6 Hz, 2 H, Ar-H), 7.32 (t, *J* = 7.6 Hz, 2 H, Ar-H), 5.31 (d, *J* = 6.8 Hz, 1 H, NH), 4.43 (d, *J* = 6.8 Hz, 2 H, NCOOC*H*_2_CH), 4.34–4.29 (m, 1 H, 2-CH), 4.23 (t, *J* = 6.8 Hz, 1 H, NCOOCH_2_C*H*), 1.93–1.89 (m, 1 H, 3-CH_2a_), 1.65–1.58 (m, 1 H, 3-CH_2b_), 1.33–1.26 (m, 12 H, 4-CH_2_, 5-CH_2_, 6-CH_2_, 7-CH_2_, 8-CH_2_, 9-CH_2_), 0.88 (t, *J* = 7.2 Hz, 3 H, 10-CH_3_) ppm. ^13^C NMR (100 MHz, CDCl_3_): *δ* = 199.3 (CHO), 156.0 (NCOO), 143.8, 143.7, 141.3 (C-Ar), 127.7, 127.1, 125.0, 120.0 (CH-Ar), 67.0 (NCOO*C*H_2_CH), 60.3 (C-2), 47.2 (NCOOCH_2_ *C*H), 31.8, 29.3, 29.2, 25.0, 22.6 (C-3, C-4, C-5, C-6, C-7, C-8, C-9), 14.1 (C-10) ppm. MS-ESI (*m/z*): calcd. for C_25_H_32_NO_3_ [M+H]^+^ 394.2377; found 394.2390.

### General procedure for synthesis of triamino acids 11, 12 and 13

Boc-L-Dab-OH (**10**, 0.5 mmol) was dissolved in 1% acetic acid (AcOH) in methanol (MeOH, 10 mL) and kept stirring at rt. The respective amino aldehydes (**7–9**, 0.46 mmol) were added into the reaction mixture slowly followed by addition of NaBH_3_CN (1.14 mmol). The reaction mixture was stirred at rt for 18 h. The progress of the reaction was monitored by TLC. On attaining maximum conversion, the reaction mixture was evaporated to dryness and was dissolved in ethylacetate (20 mL). Organic layer was washed with water (10 mL) and brine (10 mL x 2), dried over Na_2_SO_4_ and evaporated to dryness under reduced pressure to get crude compounds. Pure compounds (**11**–**13**) were obtained by purification of the crude by silica gel column chromatography (Fig 3).

#### (2*S*,2'*S*)-*N* ^2^-(*tert*-Butoxycarbonyl)-*N* ^4^-[*N* ^2'^-(9-fluorenylmethyloxycarbonyl)-2'-aminohexyl]-2,4-diaminobutanoic acid (11)

Compound **11** was purified by flash column chromatography using 0 to 15% MeOH in dichloromethane containing 1% AcOH as eluent to afford a white amorphous solid (0.265 g; 50%); m.p. 93–97°C. R_*f*_ = 0.34 (MeOH/AcOH/DCM, 7.5:1:91.5, v/v/v) ^1^H NMR (400 MHz, CDCl_3_): *δ* = 7.72 (d, *J* = 7.2 Hz, 2 H, Ar-H), 7.59 (d, *J* = 7.2 Hz, 2 H, Ar-H), 7.36 (t, *J* = 7.2 Hz, 2 H, Ar-H), 7.27 (d, *J* = 7.6 Hz, 2 H, Ar-H), 5.89 (br s, 1 H, NHCOO), 5.79 (br s, 1 H, NHCOO), 4.40–4.34 (m, 1 H, 2-CH), 4.25–4.17 (m, 2 H, NCOOC*H*_2_CH), 4.02–3.96 (m, 1 H, NCOOCH_2_C*H*), 3.88–3.85 (m, 1 H, 2'-CH), 3.26–3.13 (m, 2 H, 4-CH_2_) 3.03–2.93 (m, 2 H, 1'-CH_2_), 2.12–2.01 (m, 3 H, 3-CH_2a_, 3'-CH_2_), 1.75–1.68 (m, 1 H, NH), 1.43–1.32 (m, 14 H, 3-CH_2b_, 4'-CH_2_, 5'-CH_2_, C(CH_3_)_3_), 0.90–0.86 (m, 3 H, 6'-CH_3_) ppm. ^13^C NMR (100 MHz, CDCl_3_): *δ* = 177.6 (COOH), 156.7, 155.7 (2 x NCOO), 144.1, 141.3 (C-Ar), 127.7, 127.1, 125.5, 125.4, 120.0 (CH-Ar), 79.7 (*C*(CH_3_)_3_), 67.1 (NCOO*C*H_2_CH), 51.4 (C-2), 50.8 (C-1'), 49.6 (C-2'), 48.7 (C-4), 47.3 (NCOOCH_2_ *C*H), 32.2, 31.7, 31.0 (C-3, C-3', C-4'), 28.5 (C(*C*H_3_)_3_), 22.4 (C-5'), 14.1 (C-6') ppm. MS-ESI (*m/z*): calcd. for C_30_H_40_N_3_O_6_ [M-H]^-^ 538.2923; found 538.2918.

#### (2*S*,2'*S*)-*N* ^2^-(*tert*-Butoxycarbonyl)-*N* ^4^-[*N* ^2'^-(9-fluorenylmethyloxycarbonyl)-2'-aminooctyl]-2,4-diaminobutanoic acid (12)

Compound **12** It was purified by flash column chromatography using 0 to 15% MeOH in dichloromethane containing 1% AcOH as eluent to afford a white amorphous solid (0.253 g; 57%); m.p. 72–76°C. R_*f*_ = 0.46 (MeOH/AcOH/DCM, 7.5:1:91.5, v/v/v) ^1^H NMR (400 MHz, CDCl_3_): *δ* = 7.73 (d, *J* = 6.8 Hz, 2 H, Ar-H), 7.60 (t, *J* = 6.0 Hz, 2 H, Ar-H), 7.36 (t, *J* = 6.4 Hz, 2 H, Ar-H), 7.29–7.23 (m, 2 H, Ar-H), 5.89 (br s, 1 H, NHCOO), 5.79 (br s, 1 H, NHCOO), 4.39–4.34 (m, 1 H, 2-CH), 4.22–4.15 (m, 2 H, NCOOC*H*_2_CH), 4.03–3.86 (m, 2 H, NCOOCH_2_C*H*, 2'-CH), 3.29–3.06 (m, 2 H, 4-CH_2_), 3.00–2.91 (m, 2 H, 1'-CH_2_), 2.10–1.96 (m, 3 H, 3-CH_2_, 3'-CH_2a_), 1.72–1.69 (m, 1 H, NH), 1.38–1.09 (m, 18 H, C(CH_3_)_3_, 3'-CH_2b_, 4'-CH_2_, 5'-CH_2_, 6'-CH_2_, 7'-CH_2_), 0.91–0.78 (m, 3 H, 8'-CH_3_) ppm. ^13^C NMR (100 MHz, CDCl_3_): *δ* = 177.7 (COOH), 156.7, 155.7 (2 x NCOO), 144.2, 141.4 (C-Ar), 127.7, 127.2, 125.5, 125.4, 120.0 (CH-Ar), 79.7 (*C*(CH_3_)_3_), 67.1 (NCOO*C*H_2_CH), 53.6 (C-2), 51.4 (C-1'), 50.7 (C-2'), 48.7 (C-4), 47.2 (NCOOCH_2_ *C*H), 32.0, 31.0, 29.1 (C-3, C-3', C-4'), 28.5 (C(*C*H_3_)_3_), 26.3 (C-5'), 26.2 (C-6'), 22.7 (C-7'), 14.2 (C-8') ppm. HRMS (ESI-TOF): calcd. for C_32_H_44_N_3_O_6_ [M–H]^–^ 566.3236, found 566.3226.

#### (2*S*,2'*S*)-*N* ^2^-(*tert*-Butyloxycarbonyl)-*N* ^4^-[*N* ^2'^-(9-fluorenylmethyloxycarbonyl)-2'-aminodecyl]-2,4-diaminobutanoic acid (13)

Compound **13** was purified by flash column chromatography using 0 to 15% MeOH in dichloromethane containing 1% AcOH as eluent to afford a white amorphous solid (0.325 g; 60%); m.p. 49–53°C. R_*f*_ = 0.29 (MeOH/AcOH/DCM, 7.5:1:91.5, v/v/v) ^1^H NMR (400 MHz, CDCl_3_): *δ* = 7.73 (d, *J* = 7.6 Hz, 2 H, Ar-H), 7.60 (t, *J* = 6.8 Hz, 2 H, Ar-H), 7.36 (t, *J* = 7.6 Hz, 2 H, Ar-H), 7.29–7.25 (m, 2 H, Ar-H), 5.90–5.86 (m, 1 H, NHCOO), 4.70–4.80 (m, 1 H, NHCOO), 4.35–4.31 (m, 1 H, 2-CH), 4.27–4.16 (m, 2 H, NCOOC*H*_2_CH), 4.04–3.86 (m, 2 H, NCOOCH_2_C*H*, 2'-CH), 3.27–3.14 (m, 2 H, 4-CH_2_), 3.02–2.92 (m, 2 H, 1'-CH_2_), 2.10–2.04 (m, 3 H, 3-CH_2_, 3'-CH_2a_), 1.77–1.57 (m, 1 H, 3'-CH_2b_), 1.38–1.19 (m, 22 H, C(CH_3_)_3_, 3'-CH_2b_, 4'-CH_2_, 5'-CH_2_, 6'-CH_2_, 7'-CH_2_, 8'-CH_2,_ 9'-CH_2_) 0.90–0.86 (m, 3 H, 10'-CH_3_) ppm. ^13^C NMR (100 MHz, CDCl_3_): *δ* = 177.6 (COOH), 156.7, 156.0 (2 x NCOO), 144.2, 144.1, 141.4 (C-Ar), 127.9, 127.8, 127.2, 125.4, 120.0 (CH-Ar), 79.9 (*C*(CH_3_)_3_), 67.2 (NCOO*C*H_2_CH), 54.6 (C-2), 51.5 (C-1'), 49.6 (C-2'), 48.8 (C-4), 47.3 (NCOOCH_2_ *C*H), 32.0, 31.0, 30.8, 29.6, 29.4 (C-3, C-3', C-4', C-5', C-6'), 28.5 (C(*C*H_3_)_3_), 26.3 (C-7'), 22.8 (C-8'), 21.0 (C-9'), 14.2 (C-10') ppm. HRMS (ESI-TOF): calcd. for C_34_H_48_N_3_O_6_ [M–H]^–^ 594.3549, found 594.3455.

### General procedure for Boc protection (14, 15 and 16)

The respective triamino acids (**11**–**13**, 0.22 mmol) were dissolved in a solvent mixture of water and dioxane (1:1, v/v, 10 mL) and then stirred at 0–5°C using an ice bath. Solid Na_2_CO_3_ (0.45 mmol) was added into the reaction mixture, followed by addition of Boc anhydride [(Boc)_2_O, 0.42 mmol]. The ice bath was removed after 1 hour and the reaction mixture was stirred at rt for 18 h. The progress of the reaction was monitored by TLC. After complete reaction, the temperature of the reaction mixture was set to 0–5°C and water (10 mL) was added. 1 M HCl was added into the reaction mixture dropwise to adjust the pH of the solution (to pH 3). The product was extracted with ethylacetate (15 mL x 3). The combined ethylacetate layers was washed with water (10 mL x 2) and brine (10 mL), dried over Na_2_SO_4_ and evaporated to dryness under reduced pressure. The crude compounds were purified by silica gel column chromatography to afford compounds **14**–**16**.

#### (2*S*,2'*S*)-*N* ^2^,*N* ^4^-Bis(*tert*-butoxycarbonyl)-*N* ^4^-[*N* ^2'^-(9-fluorenylmethyloxycarbonyl)-2'-amino-hexyl]-2,4-diaminobutanoic acid (14)

Compound **14** was purified by flash column chromatography using 0 to 100% EtOAc in hexane containing 1% AcOH as eluent to afford a white amorphous solid (0.245 g; 88%); m.p. 49–52°C. R_*f*_ = 0.47 (EtOAc/AcOH/hexane, 75:1.24, v/v/v) ^1^H NMR (400 MHz, CDCl_3_): *δ* = 7.75 (d, *J* = 7.6 Hz, 2 H, Ar-H), 7.57 (d, *J* = 6.8 Hz, 2 H, Ar-H), 7.38 (t, *J* = 7.6 Hz, 2 H, Ar-H), 7.29 (t, *J* = 6.8 Hz, 2 H, Ar-H), 4.50–4.32 (m, 2 H, NCOOC*H*_2_CH), 4.25–4.15 (m, 2 H, 2-CH, NCOOCH_2_C*H*), 3.73–3.54 (m, 3 H, 2'-CH, 1'-CH_2_), 3.42–3.38 (m, 1 H, 4-CH_2a_), 3.32–3.30 (m, 1 H, 4-CH_2b_), 3.14–2.89 (m, 2 H, 3-CH_2_), 2.12–2.00 (m, 2 H, 3'-CH_2_), 1.44–1.26 (m, 22 H, 2 x C(CH_3_)_3_, 4'-CH_2_, 5'-CH_2_), 0.92–0.86 (m, 3 H, 6'-CH_3_) ppm. ^13^C NMR (100 MHz, CDCl_3_): *δ* = 173.8 (COOH), 156.8, 156.7, 156.2 (3 x NCOO), 143.9, 141.3 (C-Ar), 127.7, 127.1, 125.2, 125.0, 120.0 (CH-Ar), 82.1, 81.1, 80.2 (2 x *C*(CH_3_)_3_), 66.9, 66.3, (NCOO*C*H_2_CH), 51.2 (C-2), 50.6 (C-1'), 49.6 (C-2'), 47.3 (NCOOCH_2_ *C*H), 43.6, 43.1 (C-4), 33.2, 32.9, 32.0, 31.1 (C-3, C-3'), 28.4 (2 x C(*C*H_3_)_3_), 27.9 (C-4'), 22.6 (C-5'), 14.0 (C-6') ppm. [α]_24_^D^ = *–*2.0 (*c* 0.1, MeOH). HRMS (ESI-TOF): calcd. for C_35_H_48_N_3_O_8_ [M–H]^–^ 638.3447, found 638.3454.

#### (2*S*,2'*S*)-*N* ^4^-Bis(*tert*-butoxycarbonyl)-*N* ^4^-[*N* ^2'^-(9-fluorenylmethyloxycarbonyl)-2'-amino-octyl]-*N* ^2^,2,4-diaminobutanoic acid (15)

Compound **15** was purified by flash column chromatography using 0 to 100% EtOAc in hexane containing 1% AcOH as eluent to afford a colourless sticky solid (0.232 g; 88%). R_*f*_ = 0.53 (EtOAc/AcOH/hexane, 75:1.24, v/v/v) ^1^H NMR (400 MHz, CDCl_3_): *δ* = 7.75 (d, *J* = 7.6 Hz, 2 H, Ar-H), 7.58 (d, *J* = 6.8 Hz, 2 H, Ar-H), 7.38 (t, *J* = 7.2 Hz, 2 H, Ar-H), 7.29 (t, *J* = 7.2 Hz, 2 H, Ar-H), 4.50–4.35 (m, 2 H, NCOOC*H*_2_CH), 4.22–4.11 (m, 2 H, 2-CH, NCOOCH_2_C*H*), 3.80–3.62 (m, 3 H, 2'-CH, 1'-CH_2_), 3.66–3.60 (m, 1 H, 4-CH_2a_), 3.49–3.44 (m, 1 H, 4-CH_2b_), 3.22–2.88 (m, 2 H, 3-CH_2_), 2.09–2.02 (m, 2 H, 3'-CH_2_), 1.44 (s, 18 H, 2 x C(CH_3_)_3_), 1.33–1.24 (m, 8 H, 4'-CH_2_, 5'-CH_2_, 6'-CH_2_, 7'-CH_2_), 0.92–0.84 (m, 3 H, 8'-CH_3_) ppm. ^13^C NMR (100 MHz, CDCl_3_): *δ* = 173.7 (COOH), 157.0, 156.24, 156.18, 155.6 (3 x NCOO), 144.1, 141.3 (C-Ar), 127.8, 127.2, 125.3, 125.1, 120.1 (CH-Ar), 82.0, 80.9, 80.8, 80.2 (2 x *C*(CH_3_)_3_), 66.3, 66.1 (NCOO*C*H_2_CH), 51.4 (C-2), 50.7 (C-1'), 47.4 (NCOOCH_2_ *C*H), 44.0, 43.0 (C-2', C-4), 33.5, 33.2, 31.8, 31.6, 30.5, 29.8, 29.4, 29.3 (C-3, C-3', C-4', C-5', C-6'), 28.5 (2 x C(*C*H_3_)_3_), 22.7 (C-7'), 14.2 (C-8') ppm. [α]_22_^D^ = –2.6 (*c* 0.3, MeOH). HRMS (ESI-TOF): calcd. for C_37_H_52_N_3_O_8_ [M–H]^–^ 666.3760, found 666.3755.

#### (2*S*,2'*S*)-*N* ^2^,*N* ^4^-Bis(*tert*-butoxycarbonyl)-*N* ^4^-[*N* ^2'^-(9-fluorenylmethyloxycarbonyl)-2'-amino-decyl]-2,4-diaminobutanoic acid (16)

Compound **16** was purified by flash column chromatography using 0 to 90% MeOH in dichloromethane containing 1% AcOH as eluent to afford a white amorphous solid (0.365 g; 79%); m.p. 44–46°C. R_*f*_ = 0.58 (EtOAc/AcOH/hexane, 75:1.24, v/v/v) ^1^H NMR (400 MHz, CDCl_3_): *δ* = 7.75 (d, *J* = 7.6 Hz, 2 H, Ar-H), 7.57 (d, *J* = 6.8 Hz, 2 H, Ar-H), 7.38 (t, *J* = 7.6 Hz, 2 H, Ar-H), 7.29 (t, *J* = 6.8 Hz, 2 H, Ar-H), 4.46–4.38 (m, 2 H, NCOOC*H*_2_CH), 4.19–4.16 (m, 2 H, 2-CH, NCOOCH_2_C*H*), 3.82–3.71 (m, 3 H, 2'-CH, 1'-CH_2_), 3.60–3.32 (m, 2 H, 4-CH_2_), 3.22–2.88 (m, 2 H, 3-CH_2_), 2.10–2.00 (m, 2 H, 3'-CH_2_), 1.44 (s, 18 H, 2 x C(CH_3_)_3_), 1.26 (m, 12 H, 4'-CH_2_, 5'-CH_2_, 6'-CH_2_, 7'-CH_2_, 8'-CH_2_, 9'-CH_2_), 0.87 (t, *J* = 6.8 Hz, 3 H, 10'-CH_3_) ppm. ^13^C NMR (100 MHz, CDCl_3_): *δ* = 174.0 (COOH), 156.8, 156.7, 156.1, 155.7 (3 x NCOO), 144.0, 141.3 (C-Ar), 127.7, 127.1, 125.2, 125.0, 120.0 (CH-Ar), 81.9, 81.0, 80.2 (2 x *C*(CH_3_)_3_), 66.8, 66.1 (NCOO*C*H_2_CH), 51.3 (C-2), 50.6, 49.6 (C-1'), 47.4 (NCOOCH_2_ *C*H), 43.9, 42.9 (C-4, C-2'), 33.4, 31.9, 30.4, 29.5, 29.3 (C-3, C-3', C-4', C-5', C-6', C-7',), 28.4 (2 x C(*C*H_3_)_3_), 25.8 (C-8'), 22.7 (C-9'), 14.1 (C-10') ppm. [α]_24_^D^ = –4.0 (*c* 0.1, MeOH). HRMS (ESI-TOF): calcd. for C_39_H_56_N_3_O_8_ [M–H]^–^ 694.4073, found 694.4063.

### General procedure for synthesis of triamino acids 22, 23 and 24

The respective *N*-Boc-L-diamino acids (**20**, **10**, **21**, 0.5 mmol) were dissolved under stirring in anhydrous methanol (10 mL, containing 1% of AcOH). *N*-(9-Fluorenylmethoxycarbonyl)glycinal (**19**, 0.46 mmol) was added to the reaction mixture under a nitrogen atmosphere, followed by the addition of sodium cyanoborohydride (1.14 mmol). The reaction mixture was stirred at room temperature for 18 h and the progress of the reaction was monitored by TLC. The solvents were evaporated *in vacuo*, and the residue was dissolved in ethyl acetate (25 mL). The organic layer was washed with water (15 mL) and brine (2 x 15 mL), dried over Na_2_SO_4_, filtered and concentrated under reduced pressure. The crude products were purified by eluting them with a solution of chloroform-methanol through a short silica gel column (Fig 4) to yield **22**–**24** respectively.

#### (*S*)-*N* ^2^-*tert*-Butoxycarbonyl-*N* ^3^-[*N*-(9-fluorenylmethyloxycarbonyl)-2-aminoethyl]-2,3-diaminopropionic acid (22)

Compound **22** was purified by flash column chromatography using 0 to 15% MeOH in dichloromethane containing 1% AcOH as eluent to afford a colourless sticky solid (0.2 g; 43%). R_*f*_ = 0.14 (MeOH/AcOH/DCM, 10:1:89, v/v/v) ^1^H NMR (400 MHz, CD_3_OD): *δ* = 7.68 (d, *J* = 7.2 Hz, 2 H, Ar-H), 7.53 (d, *J* = 7.2 Hz, 2 H, Ar-H), 7.28 (t, *J* = 7.2 Hz, 2 H, Ar-H), 7.19 (t, *J* = 7.2 Hz, 2 H, Ar-H), 4.28 (d, *J* = 6.8 Hz, 2 H, NCOOC*H*_2_CH), 4.09 (t, *J* = 6.8 Hz, 1 H, NCOOCH_2_C*H*), 4.03 (t, *J* = 6.0 Hz, 1 H, 2-CH), 3.35–3.32 (m, 2 H, NCH_2_C*H*_2_NHFmoc), 3.15–3.14 (m, 2 H, 3-CH_2_), 3.08–3.05 (m, 2 H, NC*H*_2_CH_2_NHFmoc), 1.32 (s, 9 H, C(CH_3_)_3_) ppm. ^13^C NMR (100 MHz, CD_3_OD): *δ* = 177.4 (COOH), 159.3, 158.0 (2 x NCOO), 145.2, 142.6 (C-Ar), 128.8, 128.1, 126.1, 120.9 (CH-Ar), 81.0 (*C*(CH_3_)_3_), 68.1 (NCOO*C*H_2_CH), 52.8 (C-2), 51.0 (C-3), 49.1 (N*C*H_2_CH_2_NHFmoc), 48.5 (NCOOCH_2_ *C*H), 38.6 (NCH_2_ *C*H_2_NHFmoc), 28.7 (C(*C*H_3_)_3_) ppm. HRMS (ESI-TOF): calcd. for C_25_H_30_N_3_O_6_ [M–H]^–^ 468.2140, found 468.2147.

#### (*S*)-*N* ^2^-*tert*-Butoxycarbonyl-*N* ^4^-[*N*-(9-fluorenylmethyloxycarbonyl)-2-aminoethyl]-2,4-diaminobutanoic acid (23)

Compound **23** was purified by flash column chromatography using 0 to 15% MeOH in dichloromethane containing 1% AcOH as eluent to afford a colourless sticky material (0.2 g; 42%). R_*f*_ = 0.20 (MeOH/AcOH/DCM, 10:1:89, v/v/v) ^1^H NMR (400 MHz, CD_3_OD): *δ* = 7.67 (d, *J* = 7.6 Hz, 2 H, Ar-H), 7.52 (d, *J* = 7.6 Hz, 2 H, Ar-H), 7.27 (t, *J* = 7.6 Hz, 2 H, Ar-H), 7.19 (t, *J* = 7.6 Hz, 2 H, Ar-H), 4.27 (d, *J* = 6.8 Hz, 2 H, NCOOC*H*_2_CH), 4.08 (t, *J* = 6.8 Hz, 1 H, NCOOCH_2_C*H*), 3.90 (t, *J* = 6.0 Hz, 1 H, 2-CH), 3.34–3.32 (m, 2 H, NCH_2_C*H*_2_NHFmoc), 2.98–2.97 (m, 4 H, 3-CH_2_, NC*H*_2_CH_2_NHFmoc), 2.06–1.99 (m, 1 H, 4-CH_2a_), 1.90–1.80 (m, 1 H, 4-CH_2b_, merged with other peak), 1.32 (s, 9 H, C(CH_3_)_3_) ppm. ^13^C NMR (100 MHz, CD_3_OD): *δ* = 179.9 (COOH), 159.2, 159.0 (2 x NCOO), 145.2, 142.6 (C-Ar), 128.8, 128.1, 126.1, 120.9 (CH-Ar), 80.7 (*C*(CH_3_)_3_), 68.1 (NCOO*C*H_2_CH), 54.9 (C-2), 48.9 (C-3), 48.3 (NCOOCH_2_ *C*H), 46.5 (C-4), 38.5 (N*C*H_2_CH_2_NHFmoc), 31.2 (NCH_2_ *C*H_2_NHFmoc), 28.7 (C(*C*H_3_)_3_) ppm. HRMS (ESI-TOF): calcd. for C_26_H_32_N_3_O_6_ [M–H]^–^ 482.2297, found 482.2286.

### General procedure for synthesis of final monomers 25, 26 and 27

The respective triamino acids (**22–24**, 0.22 mmol) were dissolved in a solvent mixture of 1, 4-dioxane and water (1:1, v/v, 10 mL) under stirring at rt. After cooling in an ice-water bath solid Na_2_CO_3_ (0.45 mmol) was added, followed by the addition of di-*tert*-butyl dicarbonate (0.42 mmol). The ice-water bath was removed after 1 h and the reaction mixture was stirred at room temperature for 18 h. Progress of the reaction was monitored by TLC and after complete consumption of starting material, the reaction mixture was chilled in an ice-water bath, water was added and the pH of the solution was adjusted to pH 3 by dropwise addition of 1 M HCl. The product was extracted with ethyl acetate (15 mL x 3). The organic phase was washed with water (15 mL) and brine (2 x 15 mL), dried over Na_2_SO_4_, filtered and concentrated to dryness under reduced pressure to get a crude product. Pure compounds (**25**–**27**) were obtained by passing them through column of silica gel and eluting with solvent gradient of EtOAc in hexane containing 1% acetic acid.

#### (*S*)-*N* ^2^,*N* ^3^-Bis-*tert*-butoxycarbonyl-*N* ^3^-[*N*-(9-fluorenylmethyloxycarbonyl)-2-aminoethyl]-2,3-diaminopropionic acid (25)

Compound **25** was purified by flash column chromatography using 0 to 90% EtOAc in hexane containing 1% AcOH as eluent to afford a white amorphous solid (0.084 g; 67%); m.p. 76–80°C. R_*f*_ = 0.20 (EtOAc/AcOH/hexane, 80:1:19, v/v/v) ^1^H NMR (400 MHz, CDCl_3_): *δ* = 7.67 (d, *J* = 7.2 Hz, 2 H, Ar-H), 7.51 (d, *J* = 7.2 Hz, 2 H, Ar-H), 7.31 (t, *J* = 7.2 Hz, 2 H, Ar-H), 7.21 (t, *J* = 7.2 Hz, 2 H, Ar-H), 4.45–4.30 (m, 3 H, 2-CH, NCOOC*H*_2_CH), 4.17–4.09 (m, 1 H, NCOOCH_2_C*H*), 3.50 (br s, 2 H, NCH_2_C*H*_2_NHFmoc), 3.37–3.14 (m, 4 H, 3-CH_2_, NC*H*_2_CH_2_NHFmoc), 1.36 (s, 18 H, 2 x C(CH_3_)_3_) ppm. ^13^C NMR (100 MHz, CDCl_3_): *δ* = 173.8 (COOH), 157.1, 156.7, 156.3, 155.7 (3 x NCOO), 144.0, 141.4 (C-Ar), 127.8, 127.2, 125.2, 120.1 (CH-Ar), 81.7, 81.4, 80.5 (2 x *C*(CH_3_)_3_), 67.6, 67.1 (NCOO*C*H_2_CH), 54.1, 53.0 (C-2), 50.1, 49.5, 48.6, 47.6 (C-3, N*C*H_2_CH_2_NHFmoc), 47.3 (NCOOCH_2_ *C*H), 40.7, 40.0 (NCH_2_ *C*H_2_NHFmoc), 28.4 (2 x C(*C*H_3_)_3_) ppm. [α]_24_^D^ = –11.0 (*c* 0.1, MeOH). HRMS (ESI-TOF): calcd. for C_30_H_38_N_3_O_8_ [M–H]^–^ 568.2664, found 568.2670.

#### (*S*)-*N* ^2^,*N* ^4^-Bis-*tert*-butoxycarbonyl-*N* ^4^-[*N*-(9-fluorenylmethyloxycarbonyl)-2-aminoethyl]-2,4-diaminobutanoic acid (26)

Compound **26** was purified by flash column chromatography using 0 to 15% MeOH in dichloromethane containing 1% AcOH as eluent to afford a white amorphous solid (0.085 g; 66%); m.p. 71–76°C. R_*f*_ = 0.28 (EtOAc/AcOH/hexane, 80:1:19, v/v/v) ^1^H NMR (400 MHz, CDCl_3_): *δ* = 7.69–7.67 (d, *J* = 7.6 Hz, 2 H, Ar-H), 7.53 (d, *J* = 7.2 Hz, 2 H, Ar-H), 7.32 (td, *J* = 7.2, 3.6 Hz, 2 H, Ar-H), 7.22 (td, *J* = 7.6, 2.0 Hz, 2 H, Ar-H), 4.45–4.44 (m, 1 H, NCOOCH_2_C*H*), 4.24–4.19 (m, 1 H, 2-CH), 4.13–4.10 (m, 2 H, NCOOC*H*_2_CH), 3.77–3.70 (m, 1 H, 3-CH_2a_), 3.30–3.25 (m, 3 H, NC*H*_2a_C*H*_2_NHFmoc), 2.98–2.94 (m, 1 H, NC*H*_2b_CH_2_NHFmoc), 2.87–2.83 (m, 1 H, 3-CH_2b_), 2.04–1.94 (m, 1H, 4-CH_2a_), 1.76–1.71 (m, 1 H, 4-CH_2b_), 1.39–1.35 (2s, 18 H, 2 x C(CH_3_)_3_) ppm. ^13^C NMR (100 MHz, CDCl_3_): *δ* = 173.0 (COOH), 158.4, 156.9, 155.6 (3 x NCOO), 144.1, 141.4 (C-Ar), 127.8, 127.2, 125.3, 120.1 (CH-Ar), 82.8, 81.7, 80.5 (2 x *C*(CH_3_)_3_), 67.1, 66.9 (NCOO*C*H_2_CH), 51.2 (C-2), 50.3 (C-3), 47.4 (NCOOCH_2_ *C*H), 46.3, 40.7, 34.4, 29.8 (C-4, N*C*H_2_ *C*H_2_NHFmoc), 28.5, 28.4 (2 x C(*C*H_3_)_3_) ppm. [α]_22_^D^ = –2.3 (*c* 0.3, MeOH). HRMS (ESI-TOF): calcd. for C_31_H_40_N_3_O_8_ [M–H]^–^ 582.2821, found 582.2816.

#### (*S*)-*N* ^2^,*N* ^5^-Bis-*tert*-butoxycarbonyl-*N* ^5^-[*N*-(9-fluorenylmethyloxycarbonyl)-2-aminoethyl]-2,5-diaminopentanoic acid (27)

Compound **27** was purified by flash column chromatography using 0 to 15% MeOH in dichloromethane containing 1% AcOH as eluent to afford a white sticky solid material (0.097 g; 74%). R_*f*_ = 0.17 (EtOAc/AcOH/hexane, 80:1:19, v/v/v) ^1^H NMR (400 MHz, CDCl_3_): *δ* = 7.67 (d, *J* = 7.6 Hz, 2 H, Ar-H), 7.45 (d, *J* = 7.6 Hz, 2 H, Ar-H), 7.30 (t, *J* = 7.6 Hz, 2 H, Ar-H), 7.21 (t, *J* = 7.6 Hz, 2 H, Ar-H), 4.43–4.27 (m, 3 H, NCOOC*H*_2_CH, C-2), 4.11 (t, *J* = 6.8 Hz, 1 H, NCOOCH_2_C*H*), 3.26–3.02 (m, 6 H, 3-CH_2_, NC*H*_2_C*H*_2_NHFmoc), 1.74–1.71 (m, 1 H, 4-CH_2a_), 1.56–1.51 (m, 3 H, 4-CH_2b_, 5-CH_2_), 1.36 (s, 18 H, 2 x C(CH_3_)_3_) ppm. ^13^C NMR (100 MHz, CDCl_3_): *δ* = 175.3 (COOH), 157.0, 156.8, 155.8 (3 x NCOO), 144.0, 141.4 (C-Ar), 127.8, 127.1, 125.2, 120.1 (CH-Ar), 80.7, 80.5, 80.2 (2 x *C*(CH_3_)_3_), 67.0, 66.8 (NHCOO*C*H_2_CH), 54.4, 53.0, 47.3, 46.8 (C-2, N*C*H_2_CH_2_NHFmoc), 46.4 (NHCOOCH_2_ *C*H), 40.5, 40.1, 29.9 (C-5, NCH_2_ *C*H_2_NHFmoc), 28.5 (2 x C(*C*H_3_)_3_), 27.0, 24.6, 24.2 (C-3, C-4) ppm. [α]_24_^D^ = +3.0 (*c* 0.1, MeOH). HRMS (ESI-TOF): calcd. for C_32_H_42_N_3_O_8_ [M–H]^–^ 596.2977, found 596.2984.

## Supporting Information

S1 Supporting Information Experimental procedures for compounds 7, 19 and 25 as well as ^1^H NMR and ^13^C NMR spectra of compounds 4–9, 11–16 and 22–27; RP-HPLC chromatograms of purified compounds 14–16 and 25–27. (PDF)

## References

[1] Liu X, Testa BA. Fahr Lipophilicity and its relationship with passive drug permeation. Pharm Res. 2011;28: 962–977. 10.1007/s11095-010-0303-7 21052797

[2] MacKellar C, Graham D, Will DW, Burgess D, Brown T. Synthesis and physical properties of anti-HIV antisense oligonucleotides bearing terminal lipophilic groups. Nucleic Acids Res. 1992;20: 3411–3417. 1630912 10.1093/nar/20.13.3411 PMC312497

[3] Lehmann TJ, Engels JW. Synthesis and properties of bile acid phosphoramidites 5'-tethered to antisense oligodeoxynucleotides against HCV. Bioorg Med Chem. 2001;9: 1827–1835. 11425584 10.1016/s0968-0896(01)00079-7

[4] Liberska A, Lilienkampf A, Unciti-Broceta A, Bradley M. Solid-phase synthesis of arginine-based double-tailed cationic lipopeptides: potent nucleic acid carriers. Chem Commun. 2011;47: 12774–12776. 10.1039/c1cc15805h 22045294

[5] Bhattacharya S, Bajaj A. Advances in gene delivery through molecular design of cationic lipids. Chem Commun. 2009; 4632–4656. 10.1039/b900666b 19641799

[6] Xu XD, Chen JX, Cheng H, Zheng XZ, Zhuo RX. Controlled peptide coated nanostructures *via* the self-assembly of functional peptide building blocks. Polym Chem. 2012;3: 2479–2486.

[7] Cochrane SA, Lohans CT, Brandelli JR, Mulvey G, Armstrong GD, Vederas JC. Synthesis and structure-activity relationship studies of N-terminal analogues of the antimicrobial peptide tridecaptin A_1_ . J Med Chem. 2014;57: 1127–1131. 10.1021/jm401779d 24479847

[8] Zhang L, Carmichael R. Short antimicrobial lipopeptides. WO 2013/ 142088 A_1_.

[9] Flodin NW The metabolic roles, pharmacology, and toxicology of lysine. J Am Coll Nutr. 1997;16: 7–21. 9013429 10.1080/07315724.1997.10718644

[10] Griffith RS, Norins AL, Kagan CA. A multicentered study of lysine therapy in Herpes simplex infection. Dermatologica 1978;156: 257–267. 640102 10.1159/000250926

[11] Smriga M, Torii K. L-Lysine acts like a partial serotinin receptor 4 antagonist and inhibits serotonin-mediated intestinal pathologies and anxiety in rats. Proc Natl Acad Sci U S A. 2003;100: 15370–15375. 14676321 10.1073/pnas.2436556100 PMC307574

[12] McCord TJ, Cook DE, Smith LG. The synthesis and biological activities of some aza analogs of amino acids. Arch Biochem Biophys. 1964;105: 349–351. 14186741 10.1016/0003-9861(64)90018-9

[13] Marek M. On the mechanism of some antimetabolic action on the biosynthesis of the ‘cooling protein’ by pupae Galleria Mellonella. Comp Biochem Biophys. 1970;35: 737–743.

[14] Honcharenko D, Bose PP, Maity J, Kurudenkandy FR, Juneja A, Flöistrup E, et al Synthesis and evaluation of antineurotoxicity properties of an amyloid-β peptide targeting ligand containing a triamino acid. Org Biomol Chem. 2014;12: 6684–6693. 10.1039/c4ob00959b 25030615

[15] Wen JJ, Crews CM. Synthesis of 9-fluorenylmethoxycarbonyl-protected amino aldehydes. Tetrahedron: Asymmetry. 1998;9: 1855–1858.

[16] Wang G, Mahesh U, Chen GYJ, Yao SQY. Solid-phase synthesis of peptide vinyl sulfones as potential inhibitors and activity-based probes of cysteine proteases. Org Lett. 2003;5: 737–740. 12605503 10.1021/ol0275567

[17] Bandyopadhyay A, Agarwal N, Mali SM, Jadhav SV, Gopi HN. Tin(II) chloride assisted synthesis of N-protected *γ*-amino *β*-keto esters through semipinacol rearrangement. Org Biomol Chem. 2010;8: 4855–4860. 10.1039/c0ob00199f 20734011

[18] Mandal PK, Ren Z, Chen X, Xiong C, McMurray JS. Structure-affinity relationships of glutamine mimics incorporated into phosphopeptides targeted to the SH2 domain of signal transducer and activator of Transcription 3. J Med Chem. 2009;52: 6126–6141. 10.1021/jm901105k 19728728 PMC2859832

[19] Lawton GR, Appella DH. Nonionic side chains modulate the affinity and specificity of binding between functionalized polyamines and structured RNA. J Am Chem Soc. 2004;126: 12762–12763. 15469256 10.1021/ja046436m

[20] Lee JT, Chen DY, Yang Z, Ramos AD, Hsieh JJD, Bogyo M. Design, syntheses, and evaluation of Taspase1 inhibitors. Bioorg Med Chem Lett. 2009;19: 5086–5090. 10.1016/j.bmcl.2009.07.045 19631530 PMC3513416

[21] Bondebjerg J, Xiang J, Bauzo RM, Haskell-Luevano C, Meldal M. A Solid-phase approach to mouse melanocortin receptor agonists derived from a novel thioether cyclized peptidomimetic scaffold. J Am Soc Chem. 2002;124: 11046–11055. 12224952 10.1021/ja0123913

[22] Fehrentd JA, Pothinol C, Califanol JC, Loff A, Martinez J. Synthesis of chiral N-protected α-amino aldehydes by reduction of N-protected N-carboxyanhydrides (UNCAs). Tetrahedron Lett. 1994;35: 9031–9034.

[23] Ho PT, Ngu K. An effective synthesis of N-(9-fluorenylmethyloxycarbonyl) α-amino aldehydes from *S*-benzyl thioesters. J Org Chem. 1993;58: 2313–2316.

[24] Fukuyama T, Lin SC, Li L. Facile reduction of ethyl thiol esters to aldehydes: application to a total synthesis of (+)-Neothramycin A methyl ether. J Am Chem Soc. 1990;112: 7050–7051.

[25] Debaene F, Mejias L, Harrisb JL, Winssinger N. Synthesis of a PNA-encoded cystine protease inhibitor library. Tetrahedron 2004;60: 8677–8690.

[26] Neises B, Steglich W. Simple method for the esterification of carboxylic acids. Angew Chem Int Ed. 1978;17: 522–524.

[27] Tokuyoma H, Yokoshim S, Li SC, Li L, Fukiyama T. Reduction of ethanethiol esters to aldehydes. Synthesis. 2002;8: 1121–1123. 12198385

[28] Tripathi RP, Verma SS, Pandey J, Tiwari VK. Recent development on catalytic reductive amination and applications. Curr Org Chem. 2008;12: 1093–1115.

[29] Brown ZZ, Schadmeister CE. Synthesis of hexa- and pentasubstituted diketopiperazines from sterically hindered amino acids. Org Lett. 2010;12: 1436–1439. 10.1021/ol100048g 20218644

[30] Mariani R, Granata G, Maffuoli SI, Serina S, Brunati C, Sosio M, et al Antibiotics GE23077, novel inhibitors of bacterial RNA polymerase. Part 3: Chemical derivatization. Bioorg Med Chem Lett. 2005;15: 3748–3752. 15990299 10.1016/j.bmcl.2005.05.060

[31] Gros L, Lorente SO, Jimenez CJ, Yardley V, Rattray L, Wharton H, et al Evaluation of azasterols as anti-parasitics. J Med Chem. 2006;49: 6094–6103. 17004723 10.1021/jm060290f

[32] Debaene F, Da Silva JA, Pianowski Z, Duran FJ, Winssinger N. Expanding the scope of PNA-encoded libraries: divergent synthesis of libraries targeting systeine, serine and metalloproteases as well as tyrosine phosphatases. Tetrahedron. 2007;63: 6577–6586.

[33] Tedeschi T, Sforza S, Maffei F, Corradini R, Marchelli RA. Fmoc-based submonomeric strategy for the solid-phase synthesis of optically pure chiral PNAs. Tetrahedron Lett. 2008;49: 4958–4961.

[34] Chhabra SR, Mahajan A, Chen WC. Synthesis of novel, orthogonally protected multifunctional amino acids. Tetrahedron Lett. 1999;40: 4905–4908.

[35] Chhabra SR, Mahajan A, Chan WC. Homochiral 4-azalysine building blocks: syntheses and applications in solid-phase chemistry. J Org Chem. 2002;67: 4017–4029. 12054934 10.1021/jo010456e

[36] Clausen JD, Linderoth L, Nielsen HM, Franzyk H. Solid-phase route to Fmoc-protected cationic amino acid building blocks. Amino Acids. 2012;43: 1633–1641. 22358257 10.1007/s00726-012-1239-5

[37] Maity J, Stromberg R. *N* ^2^-*tert*-Butoxycarbonyl-*N* ^5^-[*N*-(9-fluorenylmethyloxycarbonyl)-2-aminoethyl]-(*S*)-2,5-diaminopentanoic acid. Molbank 2014;3: M833.

[38] Matsumori N, Masuda R, Murata M Amphotericin B. covalent dimers bearing a tartarate linkage. Chem Biodiversity. 2004;1: 346–352. 17191852 10.1002/cbdv.200490030

