# Supplementary material for: Synthesis of Triamino Acid Building Blocks with Different Lipophilicities
Source: PLoS One. 2015 Apr 14;10(4):e0124046. doi: 10.1371/journal.pone.0124046 (PMC4397077; doi:10.1371/journal.pone.0124046)
Supplement: S1 Supporting Information — (PDF) [file pone.0124046.s001.pdf]

Supporting Information

for

Synthesis of Triamino Acid Building Blocks with Different Lipophilicities

Jyotirmoy Maity, Dmytro Honcharenko and Roger Stromberg*

Department of Biosciences and Nutrition, Karolinska Institute (KI), Huddinge, Sweden. Roger.Stromberg@ki.se

(*S*)-*N*-(9-Fluorenylmethoxycarbonyl)-2-aminohexanal (7) [25]

Ethylthio ester **4** (0.36 g, 0.9 mmol) was dissolved in dry acetone (12 mL) under inert atmosphere. 10% Pd/C (0.15 g) was added to the solution followed by addition of triethylsilane (0.217 mL, 1.34 mmol) whereupon the mixture was stirred at room temperature. Progress of the reaction was monitored by TLC on silica gel. After 2 h, the reaction was stopped by passing it through a short pad of celite and washed with acetone (3 x 20 mL). The combined organic layers was evaporated to dryness under reduced pressure and dissolved in ethylacetate (15 mL). After washing the organic layer with brine (2 x 8 mL) it was dried over Na_2SO_4 and concentrated under reduced pressure to get the crude product. The

compound **7** was purified by flash column chromatography using 0 to 50% EtOAc in hexane to afford a white amorphous solid (0.257 g; 84%); m.p. 92-94 ^{o}C. R_f = 0.22 (EtOAc/hexane, 1:5, v/v). ^{1}H NMR (400 MHz, $CDCl_3$): δ = 9.58 (s, 1 H, CHO), 7.76 (d, *J* = 7.6 Hz, 2 H, Ar-H), 7.60 (d, *J* = 7.2 Hz, 2 H, Ar-H), 7.40 (t, *J* = 7.2 Hz, 2 H, Ar-H), 7.32 (t, *J* = 7.6 Hz, 2 H, Ar-H), 5.31 (d, *J* = 6.8 Hz, 1 H, NH), 4.43 (d, *J* = 6.8 Hz, 2 H, NCOOCH_2CH), 4.32 (m, 1 H, 2-CH), 4.23 (t, *J* = 6.8 Hz, 1 H, $NCOOCH_2C$*H*), 1.93-1.89 (m, 1 H, 3-CH_{2a}), 1.63-1.58 (m, 1 H, 3-CH_{2b}), 1.33-1.24 (m, 4 H, 4-CH_2, 5-CH_2), 0.91 (t, *J* = 6.0 Hz, 3 H, 6-CH_3) ppm. ^{13}C NMR (100 MHz, $CDCl_3$): δ = 199.4 (CHO), 156.2 (NCOO), 144.0, 143.9, 141.5 (C- Ar), 127.9, 127.2, 125.2, 120.1 (CH-Ar), 67.1 (NCOO*C*H_2CH), 60.4 (C-2), 47.4 ($NCOOCH_2$*C*H), 29.0 (C-3), 27.3 (C-4), 22.6 (C-5), 14.0 (C-6) ppm. MS-ESI (*m/z*): calcd. for $C_{21}H_{24}NO_3$ $[M+H]^+$ 338.1751; found 338.1760.

_N_-Fmoc-glycinal (19)[38]

We commenced our synthetic procedure with Fmoc protection of the inexpensive starting material 3-aminopropane-1,2-diol (**17**). The primary amine present of the molecule was protected with Fmoc by stirring it overnight at r.t. with *N*-[{(9H-fluoren-9-yl)methoxy}carbonyloxy]-succinimide (Fmoc-OSu) using methanol as solvent and pyridine as base. Fmoc-OSu (3.5 g, 0.01 mol) and 3-aminopropane-1,2-diol (1.175 g, 0.013 mol) were dissolved in methanol (100 ml) with little amount of pyridine (2.75 mL). The mixture was stirred at room temperature for overnight. On completion of the reaction, the reaction mixture was diluted with brine solution (200 mL) and extracted with ethyl acetate (120 mL x 3). The combined organic layer was dried over Na_2SO_4 and concentrated under vaccum to get *N*-Fmoc-glycinal (**18**). Compound **18** was obtained quantitatively in this reaction, which was used then without further purification. In the next step, compound **18** was converted into its corresponding aldehyde **19**. Diol-**18** (1.95 g, 6 mmol) was dissolved in THF (15 ml) and aqueous $NaIO_4$ (0.5 M solution, 36 mL) was added into

it. The reaction mixture was stirred vigorously for 1 h and the solution was treated with 1M ethylene glycol (1.35 mL) to reduce excess amount of $NaIO_4$. The resulting solution was diluted with ethyl acetate (150 mL) and washed with brine solution, dried over Na_2SO_4. Removal of solvent under reduced pressure gave crude reaction mixture, which was purified by silica gel column chromatography (1-3% methanol in dichloromethane) to afford pure *N*-Fmoc-glycinal (**19**, 1.59 g, 91%). R_f = 0.40 (MeOH/DCM, 1:19, v/v). ^{1}H NMR (400 MHz, DMSO-d_6): δ = 9.50 (s, 1 H, CHO), 7.95-7.93 (m, 2 H, Ar-H), 7.76-7.74 (m, 2 H, Ar-H), 7.46 (t, *J* = 7.4 Hz, 2 H, Ar-H), 7.39-7.36 (m, 2 H, Ar-H), 4.38 (d, *J* = 6.8 Hz, 2 H, C$\underline{H_2}$CHO), 4.29-4.28 (m, 2 H, C$\underline{H}$-Fmoc), 3.38 (d, *J* = 5.6 Hz, 2 H, C$\underline{H_2}$-Fmoc) ppm. ^{1}H NMR (400 MHz, $CDCl_3$): δ = 9.63 (s, 1 H, CHO), 7.76-7.72 (m, 2 H, Ar-H), 7.59-7.55 (m, 2 H, Ar-H), 7.41-7.35 (m, 2 H, Ar-H), 7.32-7.28 (m, 2 H, Ar-H), 4.43 (d, *J* = 6.8 Hz, 2 H, C$\underline{H_2}$CHO), 4.22 (t, *J* = 6.8 Hz, 2 H, C$\underline{H}$-Fmoc), 4.12 (d, *J* = 4.8 Hz, 2 H, C$\underline{H_2}$-Fmoc) ppm. ^{13}C NMR (100 MHz, $CDCl_3$): δ = 195.4 (CHO), 156.2 (NCOO), 142.8, 140.3 (C- Ar), 126.7, 126.0, 124.0, 118.7 (CH-Ar), 66.2 (*C*H_2CHO), 50.6 (*C*H_2-Fmoc), 46.2 (*C*H-Fmoc) ppm.

(*S*)-*N*^{2}-*tert*-butoxycarbonyl-*N*^{5}-[*N*-(9-fluorenylmethyloxycarbonyl)-2-aminoethyl]-2,5-diaminopentanoic acid (24) [37]

N^2-*tert*-Butoxycarbonyl-L-2,5-diaminopropionic acid (**21**, Boc-L-ornithine, 0.116 g, 0.5 mmol) was dissolved in a solvent mixture (acetic acid/methanol, 1:99, v/v, 10 mL) at rt under stirring. *N*-Fmoc-glycinal (**19**, 0.129 g, 0.46 mmol) was added into the reaction mixture slowly followed by addition of $NaBH_3CN$ (0.072 g, 1.14 mmol) in a single lot. The reaction mixture was stirred at rt and the progress of the reaction was monitored by thin layer chromatography. After 18 h, the reaction mixture was evaporated to dryness under reduced pressure and was

dissolved in ethyl acetate (15 mL). Organic layer was washed with water (10 mL) and brine (10 mL × 2), dried over Na_2SO_4 and evaporated to dryness under reduced pressure to get crude compound. Pure compound was obtained by purification of the crude using column chromatography (2%–4% methanol in dichloromethane containing 1% acetic acid) to afford pure compound **24** (0.104 g, 42%) as a white sticky solid. R_f = 0.17 (Methanol/dichloromethane/acetic acid = 1:8.9:0.1, v/v/v). ^{1}H-NMR (400 MHz, CD_3OD) (δ/ppm): 7.70 (2H, d, *J* = 7.6 Hz, Ar-H), 7.55 (2H, d, *J* = 7.6 Hz, Ar-H), 7.29 (2H, d, *J* = 7.6 Hz, Ar-H), 7.21 (2H, d, *J* = 7.6 Hz, Ar-H), 4.32 (2H, d, *J* = 6.8 Hz, NCOOCH_2CH), 4.11 (1H, t, *J* = 6.8 Hz, NCOOCH_2C*H*), 3.86 (1H, br s, 2-CH), 3.31 (2H, t, *J* = 5.6 Hz, NCH_2CH_2NHFmoc), 2.98 (2H, t, *J* = 5.6 Hz, NCH_2CH_2NHFmoc), 2.90 (2H, t, *J* = 5.6 Hz, 5-CH_2), 1.74–1.61 (4H, m, 3-CH_2, 4-CH_2), 1.32 (9H, s, C$(CH_3)_3$). ^{13}C-NMR (100 MHz, CD_3OD) (δ/ppm), methine and methyl carbons were distinguished from methylene carbons by ^{13}C-DEPT: 179.5 (COOH), 159.2, 157.6 (2 × NCOO), 145.2, 142.6 (C-Ar), 128.8, 128.1, 126.1, 120.9 (CH-Ar), 80.2 (*C*$(CH_3)_3$), 68.0 (NCOO*C*H_2CH), 56.2 (C-2), 48.8, 48.6 (C-5, N*C*H_2CH_2NHFmoc), 48.4 (NCOOCH_2*C*H), 38.5 (NCH_2*C*H_2NHFmoc), 31.3 (C-3), 28.8 (C(*C*$H_3)_3$), 23.5 (C-4). IR (KBr) νmax 3433, 1700, 1570, 1415, 1355, 1250, 1160, 1050, 957, 815, 725, 692 and 620 cm^{-1}. $[\alpha]_{27}^{D}$ = +8.2 (*c* 1.0, MeOH). HRMS (ESI-Tof) *(m/z)* calcd for $C_{27}H_{34}N_3O_6$ $[M-H]^-$ 496.2453; found 496.2463.

Reversed-phase HPLC analysis of compounds 14–16 and 25–27

RP-HPLC analysis was accomplished on a Jasco apparatus using a Phenomenex Jupiter 4u Proteo 90A (250 × 4.6 mm, 4 μm) column. The UV detection was carried out at 262 nm (Fmoc-amino acids). Conditions: compounds **14** (t_R 7.45 min) and **15** (t_R 10.56 min), a flow rate of 1.2 ml/min and a linear gradient from 80% to 100% of solvent B in solvent A (solvent A = 0.1% TFA in water; solvent B = 0.1% TFA in 90%

aqueous acetonitrile) over 20 min were used; compound **16** (t_R 7.80 min), a flow rate of 1.5 ml/min and a linear gradient from 90% to 100% of solvent B in solvent A over 20 min were used; compounds **25** (t_R 15.77 min), **26** (t_R 15.83 min) and **27** (t_R 15.99 min), a flow rate of 0.8 ml/min and a linear gradient from 50% to 100% of solvent B in solvent A over 20 min were used. The HPLC equipment was controlled by the Jasco ChromPass software (version 1.8.6.1). The same software was used for integration and smaller variation in the baseline and peaks below 0.1% of the total area were in general not included.

Figure S1. ^{1}H NMR spectrum of **4** ($CDCl_3$, 400 MHz).

Figure S2. ^{13}C NMR spectrum of **4** ($CDCl_3$, 100 MHz).

Figure S3. DEPT-135 spectrum of **4** ($CDCl_3$, 100 MHz).

Figure S4. ^{1}H NMR spectrum of **5** ($CDCl_3$, 400 MHz).

Figure S5. ^{13}C NMR spectrum of **5** ($CDCl_3$, 100 MHz).

Figure S6. DEPT-135 spectrum of **5** (CD_3OD, 100 MHz).

Figure S7. ^{1}H NMR spectrum of **6** ($CDCl_3$, 400 MHz).

Figure S8. ^{13}C NMR spectrum of **6** ($CDCl_3$, 100 MHz).

Figure S9. DEPT-135 spectrum of **6** ($CDCl_3$, 100 MHz).

Figure S10. ^{1}H NMR spectrum of **7** ($CDCl_3$, 400 MHz).

Figure S11. ^{13}C NMR spectrum of **7** ($CDCl_3$, 100 MHz).

Figure S12. DEPT-135 spectrum of **7** ($CDCl_3$, 100 MHz).

Figure S13. ^{1}H NMR spectrum of **8** ($CDCl_3$, 400 MHz).

Figure S14. ^{1}H-^{1}H COSY spectrum of **8** in $CDCl_3$.

Figure S15. ^{13}C NMR spectrum of **8** ($CDCl_3$, 100 MHz).

Figure S16. DEPT-135 spectrum of **8** ($CDCl_3$, 100 MHz).

Figure S17. ^{1}H NMR spectrum of **9** ($CDCl_3$, 400 MHz).

Figure S18. ^{13}C NMR spectrum of **9** ($CDCl_3$, 100 MHz).

Figure S19. DEPT-135 spectrum of **9** ($CDCl_3$, 100 MHz).

Figure S20. ^{1}H NMR spectrum of **11** ($CDCl_3$, 400 MHz).

Figure S21. ^{13}C NMR spectrum of **11** ($CDCl_3$, 100 MHz).

Figure S22. DEPT-135 spectrum of **11** ($CDCl_3$, 100 MHz).

Figure S23. ^{1}H NMR spectrum of **12** ($CDCl_3$, 400 MHz).

Figure S24. ^{13}C NMR spectrum of **12** ($CDCl_3$, 100 MHz).

Figure S25. DEPT-135 spectrum of **12** ($CDCl_3$, 100 MHz).

Figure S26. ^{1}H NMR spectrum of **13** ($CDCl_3$, 400 MHz).

Figure S27. ^{13}C NMR spectrum of **13** ($CDCl_3$, 100 MHz).

Figure S28. ^{1}H NMR spectrum of **14** ($CDCl_3$, 400 MHz).

Figure S29. ^{13}C NMR spectrum of **14** ($CDCl_3$, 100 MHz).

Figure S30. ^{1}H NMR spectrum of **15** ($CDCl_3$, 400 MHz).

Figure S31. ^{13}C NMR spectrum of **15** ($CDCl_3$, 100 MHz).

Figure S32. DEPT-135 spectrum of **15** ($CDCl_3$, 100 MHz).

Figure S33. ^{1}H NMR spectrum of **16** ($CDCl_3$, 400 MHz).

Figure S34. ^{13}C NMR spectrum of **16** ($CDCl_3$, 100 MHz).

Figure S35. DEPT-135 spectrum of **16** ($CDCl_3$, 100 MHz).

Figure S36. ^{1}H NMR spectrum of **22** (CD_3OD, 400 MHz).

Figure S37. ^{13}C NMR spectrum of **22** (CD_3OD, 100 MHz).

Figure S38. DEPT-135 spectrum of **22** (CD_3OD, 100 MHz).

Figure S39. ^{1}H NMR spectrum of **23** (CD_3OD, 400 MHz).

Figure S40. ^{13}C NMR spectrum of **23** (CD_3OD, 100 MHz).

Figure S41. DEPT-135 spectrum of **23** (CD_3OD, 100 MHz).

Figure S42. ^{1}H NMR spectrum of **24** (CD_3OD, 400 MHz).

Figure S43. ^{13}C NMR spectrum of **24** (CD_3OD, 100 MHz).

Figure S44. DEPT-135 spectrum of **24** (CD_3OD, 100 MHz).

Figure S45. ^{1}H NMR spectrum of **25** ($CDCl_3$, 400 MHz).

Figure S46. ^{13}C NMR spectrum of **25** ($CDCl_3$, 100 MHz).

Figure S47. DEPT-135 spectrum of **25** ($CDCl_3$, 100 MHz).

Figure S48. ^{1}H NMR spectrum of **26** ($CDCl_3$, 400 MHz).

Figure S49. ^{13}C NMR spectrum of **26** ($CDCl_3$, 100 MHz).

Figure S50. DEPT-135 spectrum of **26** ($CDCl_3$, 100 MHz).

Figure S51. ^{1}H NMR spectrum of **27** ($CDCl_3$, 400 MHz).

Figure S52. ^{13}C NMR spectrum of **27** ($CDCl_3$, 100 MHz).

Figure S53. DEPT-135 spectrum of **27** ($CDCl_3$, 100 MHz).

Figure S54. RP-HPLC chromatogram of compound **14**.

Figure S55. Analysis of RP-HPLC chromatogram of compound **14**.

Figure S56. RP-HPLC chromatogram of compound **15**.

Figure S57. Analysis of RP-HPLC chromatogram of compound **15**.

Figure S58. RP-HPLC chromatogram of compound **16**.

Figure S59. Analysis of RP-HPLC chromatogram of compound **16**.

Figure S60. RP-HPLC chromatogram of compound **25**.

Figure S61. Analysis of RP-HPLC chromatogram of compound **25**.

Figure S62. RP-HPLC chromatogram of compound **26**.

Figure S63. Analysis of RP-HPLC chromatogram of compound **26**.

Figure S64. RP-HPLC chromatogram of compound **27**.

Figure S65. Analysis of RP-HPLC chromatogram of compound **27**.

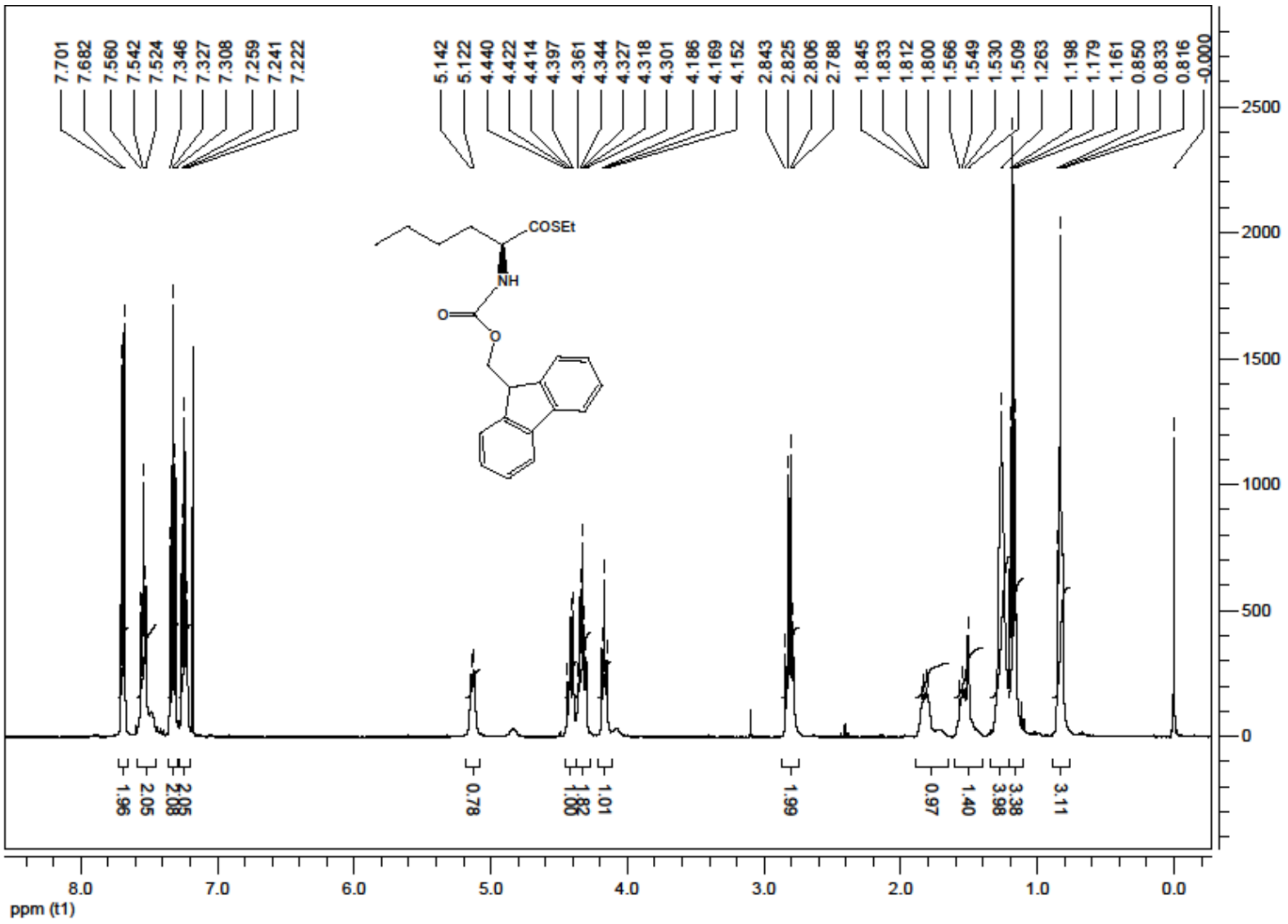

Figure S1. ^{1}H NMR spectrum of **4** ($CDCl_3$, 400 MHz).

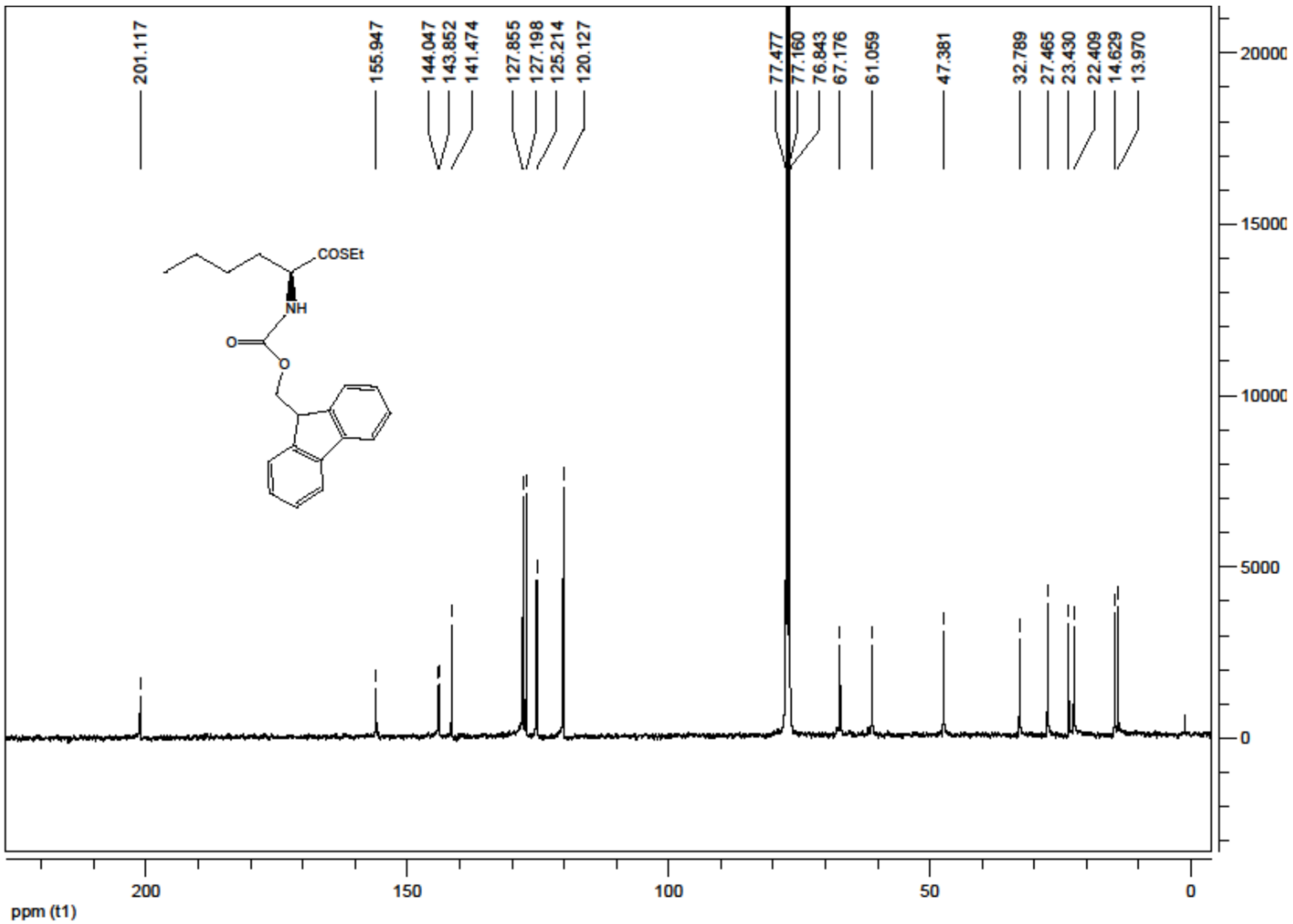

Figure S2. ^{13}C NMR spectrum of **4** ($CDCl_3$, 100 MHz).

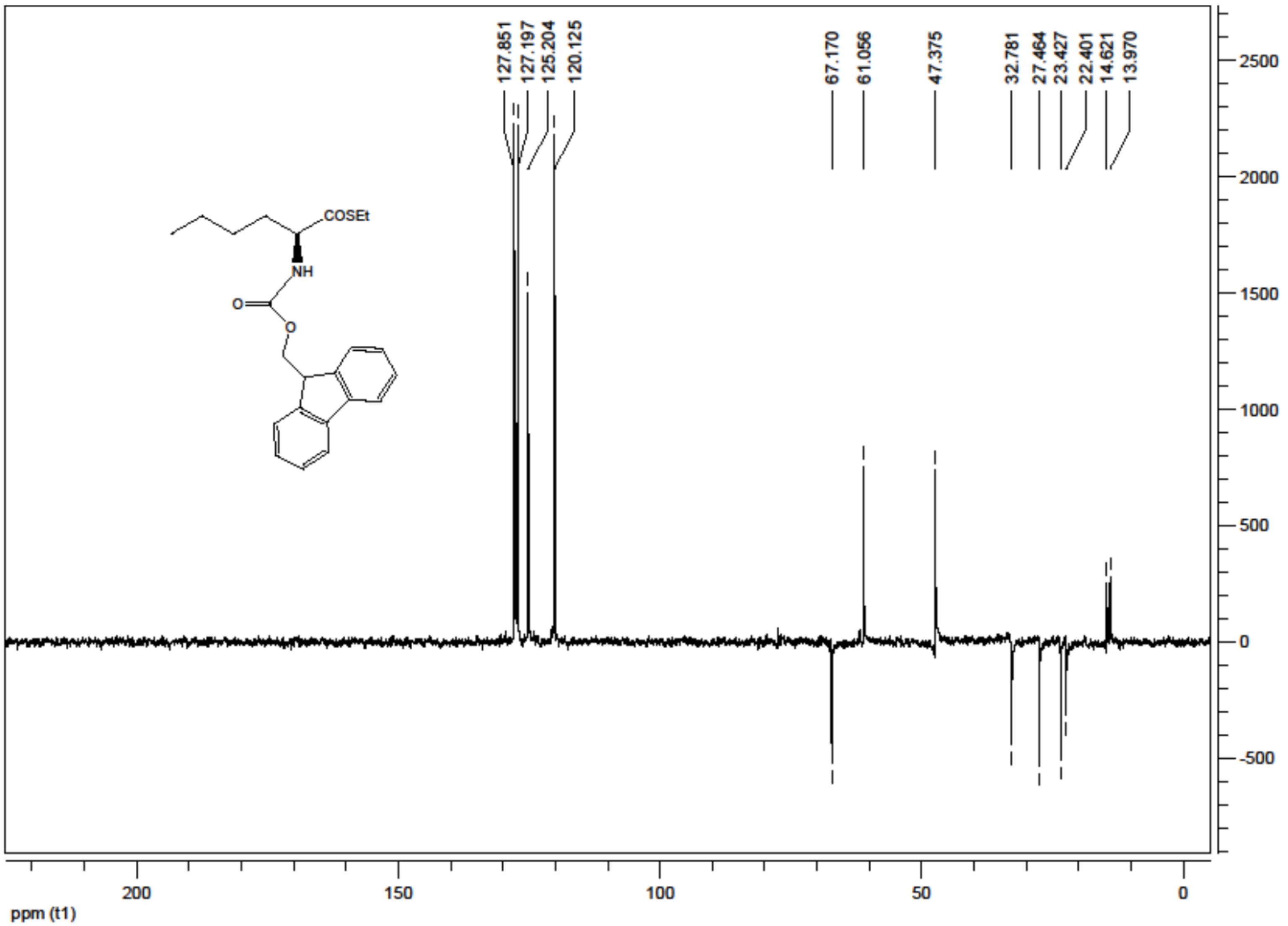

Figure S3. DEPT-135 spectrum of **4** ($CDCl_3$, 100 MHz).

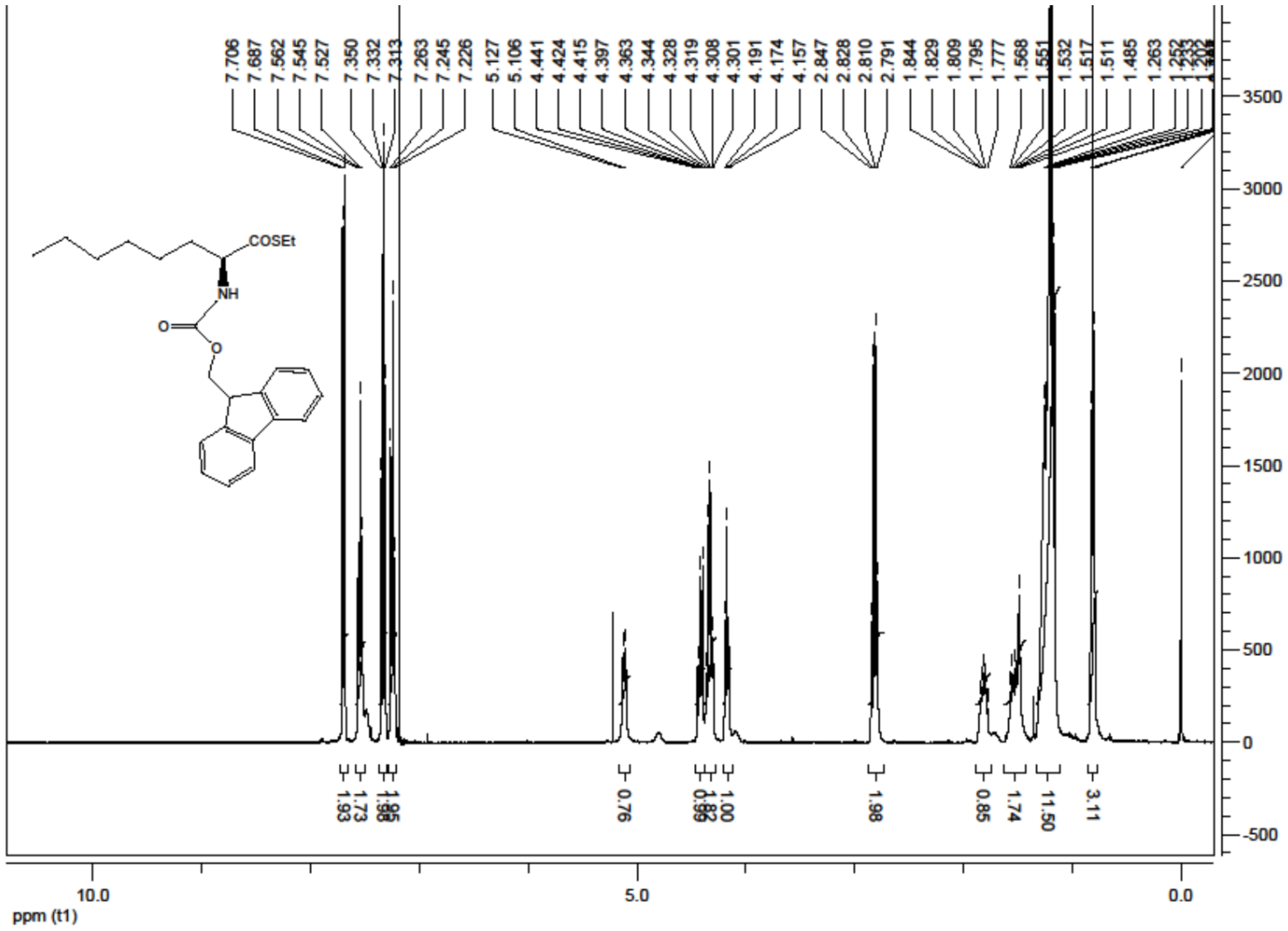

Figure S4. ^{1}H NMR spectrum of **5** ($CDCl_3$, 400 MHz).

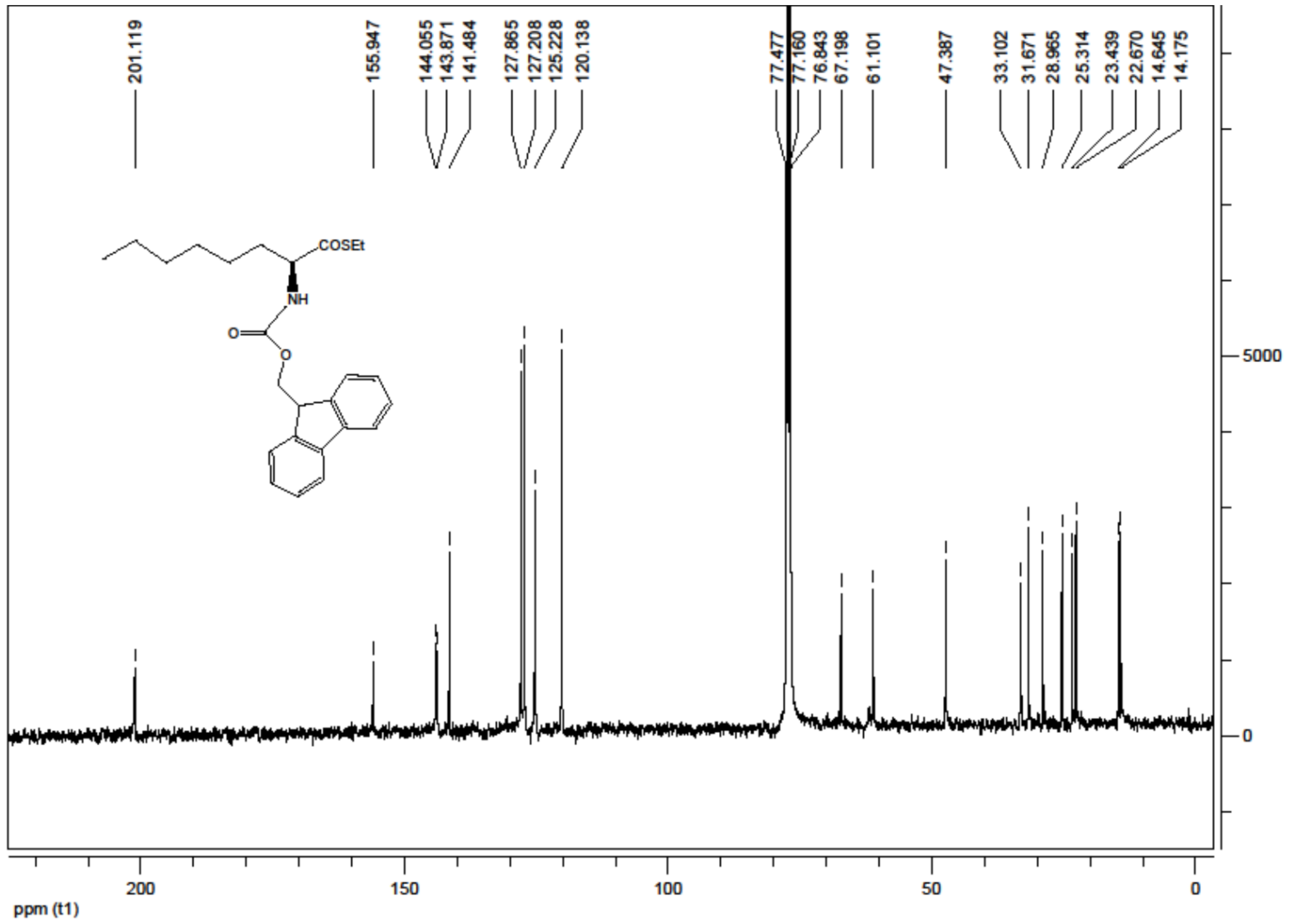

Figure S5. ^{13}C NMR spectrum of **5** ($CDCl_3$, 100 MHz).

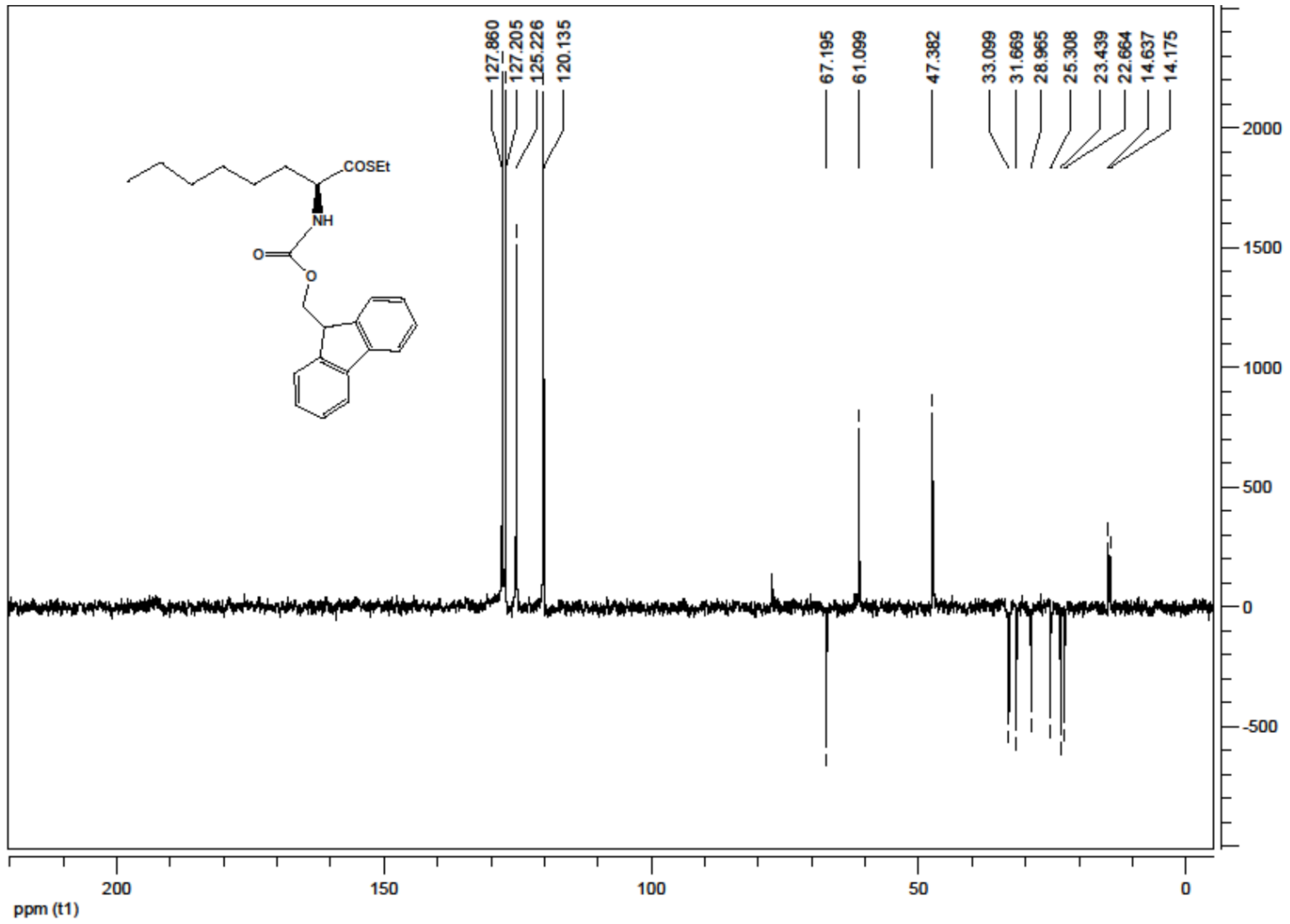

Figure S6. DEPT-135 spectrum of **5** ($CDCl_3$, 100 MHz).

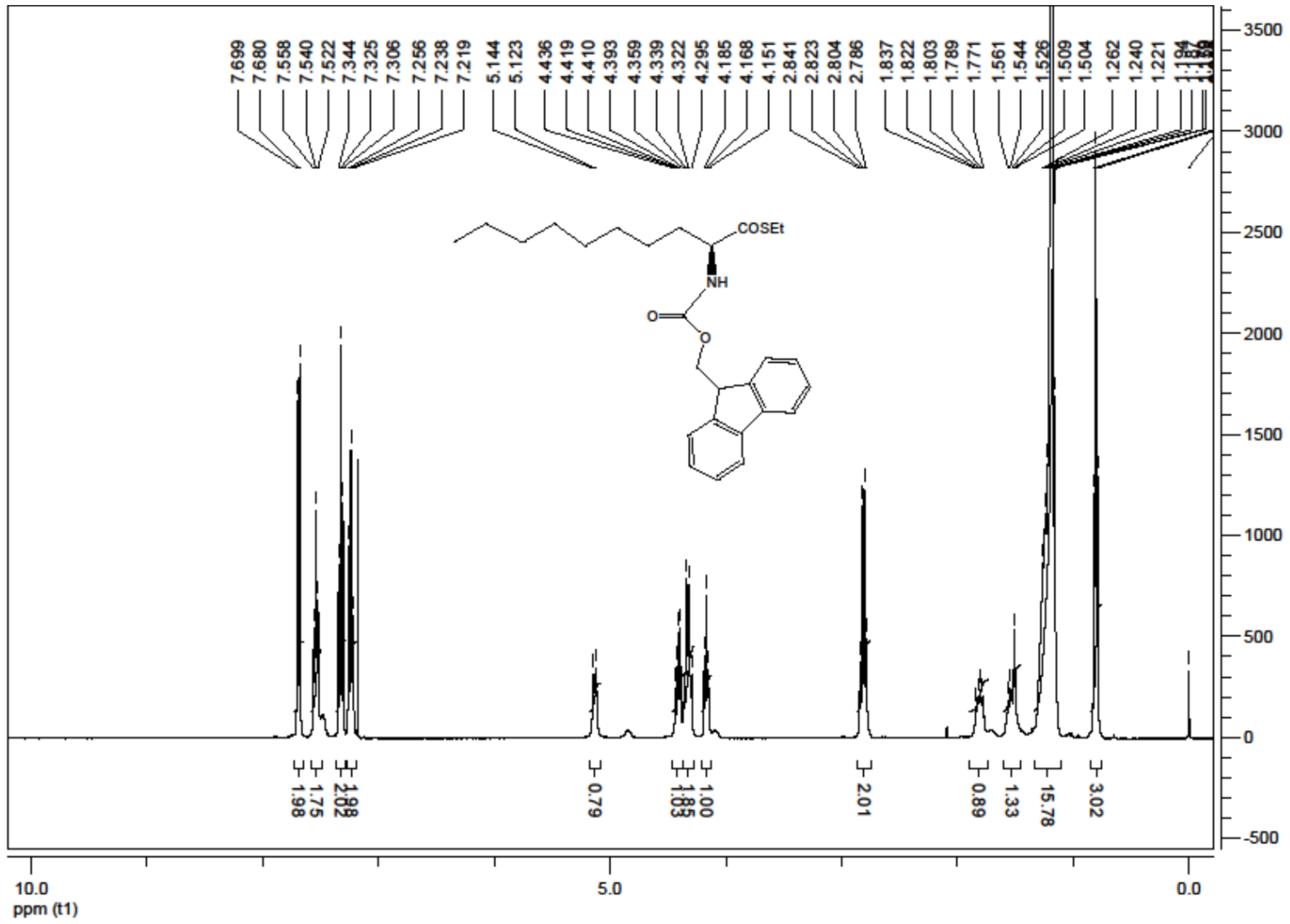

Figure S7. ^{1}H NMR spectrum of **6** ($CDCl_3$, 400 MHz).

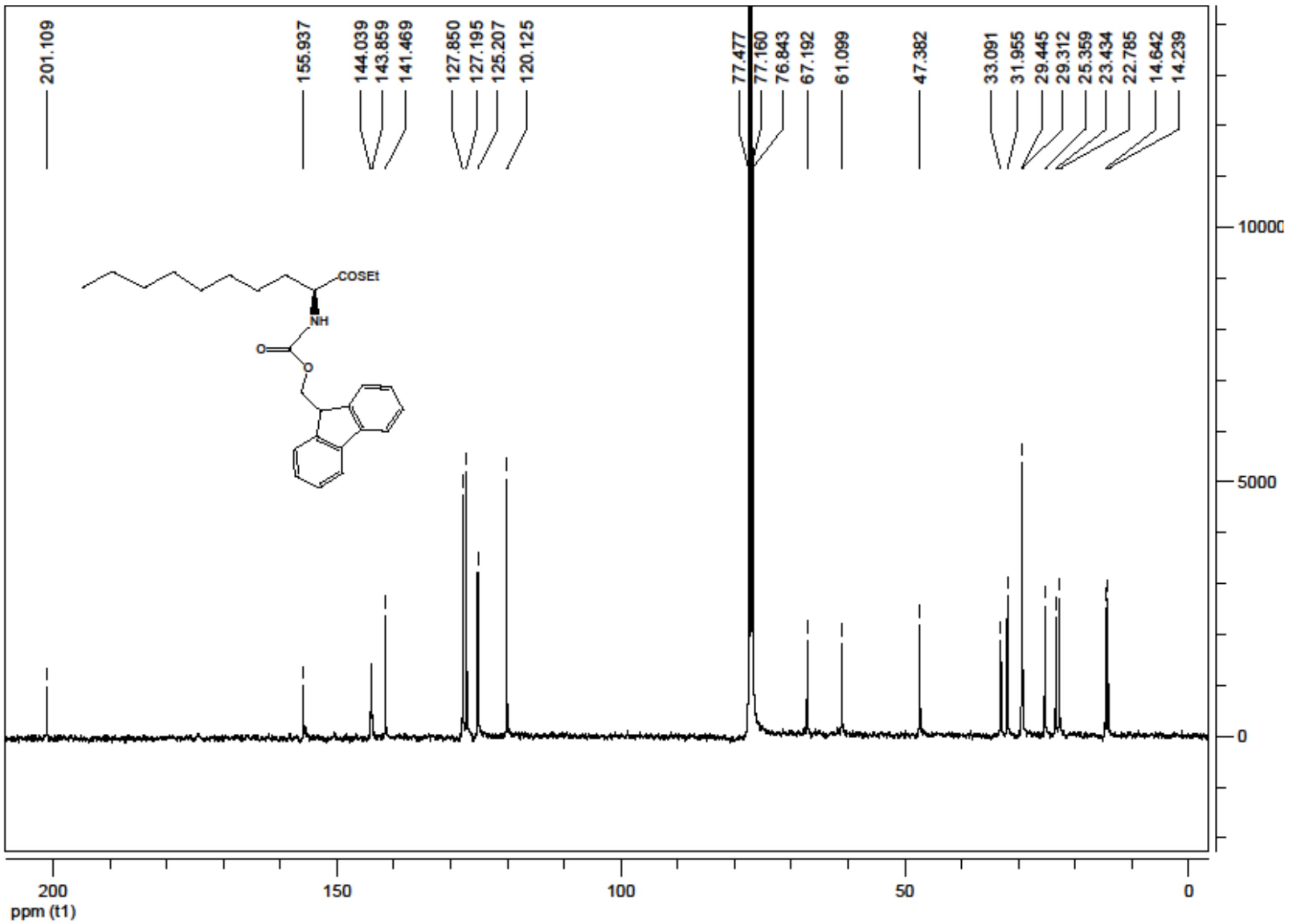

Figure S8.^{13}C NMR spectrum of **6** ($CDCl_3$, 100 MHz).

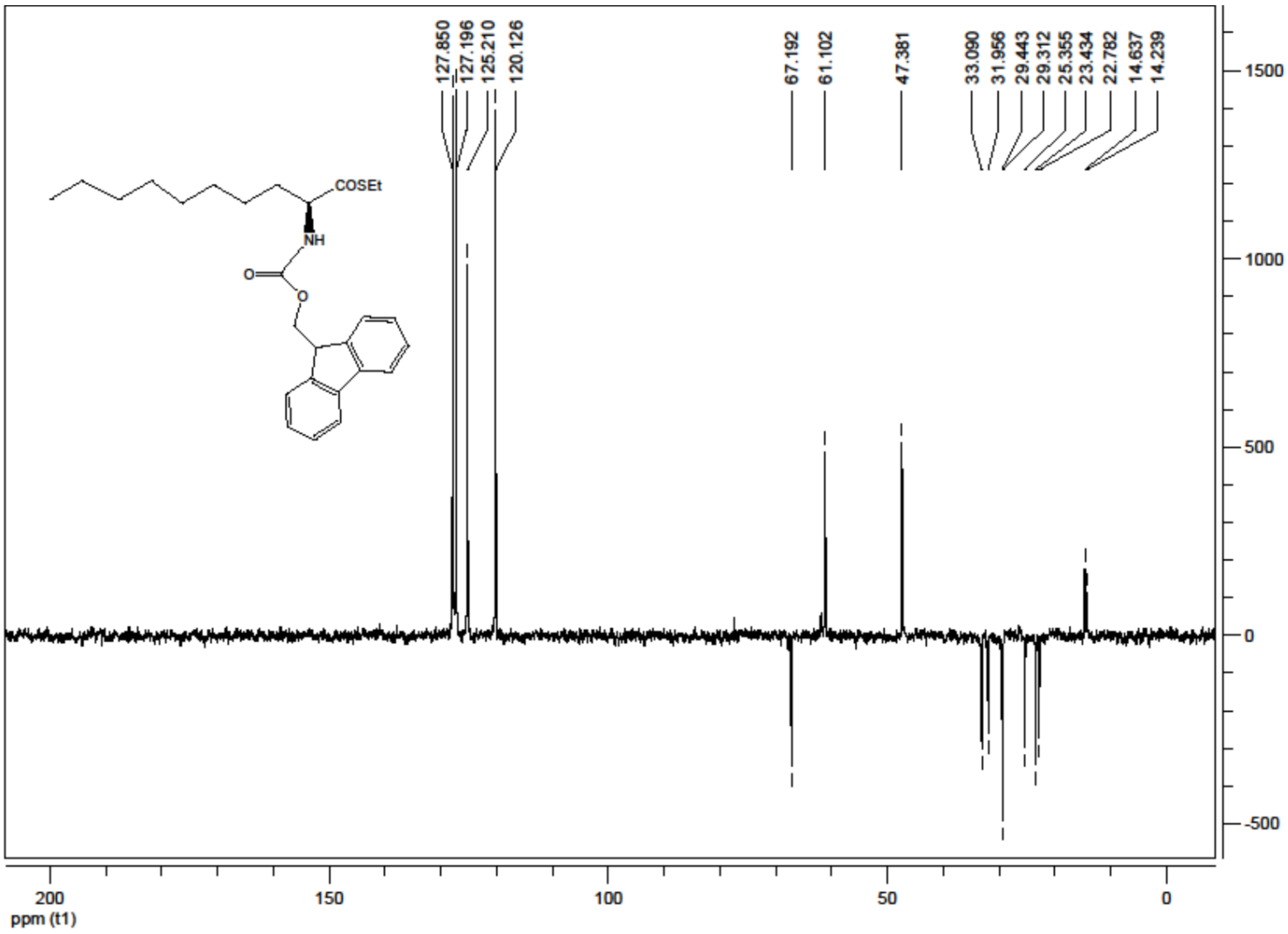

Figure S9. DEPT-135 spectrum of **6** ($CDCl_3$, 100 MHz).

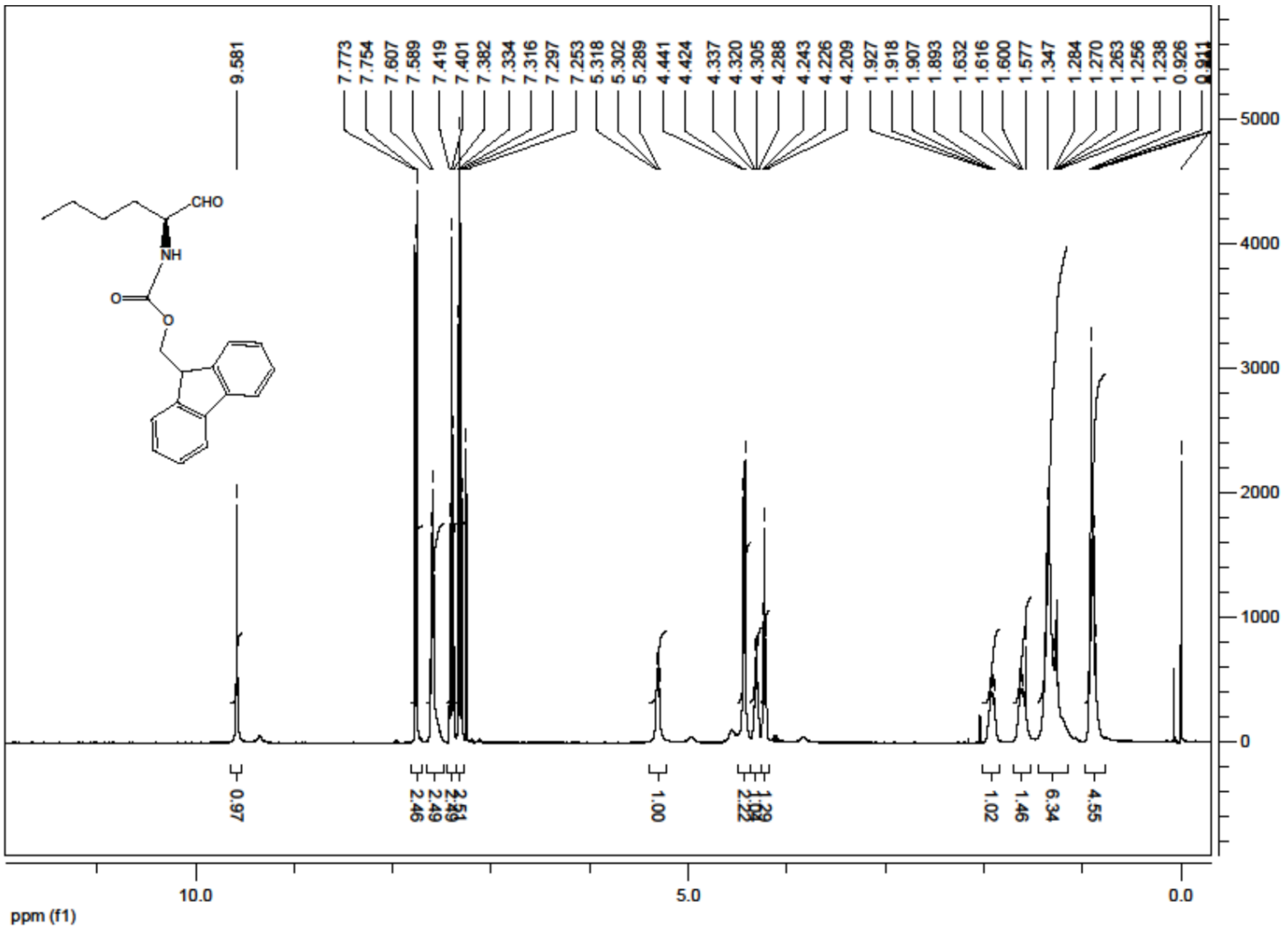

Figure S10. ^{1}H NMR spectrum of **7** ($CDCl_3$, 400 MHz).

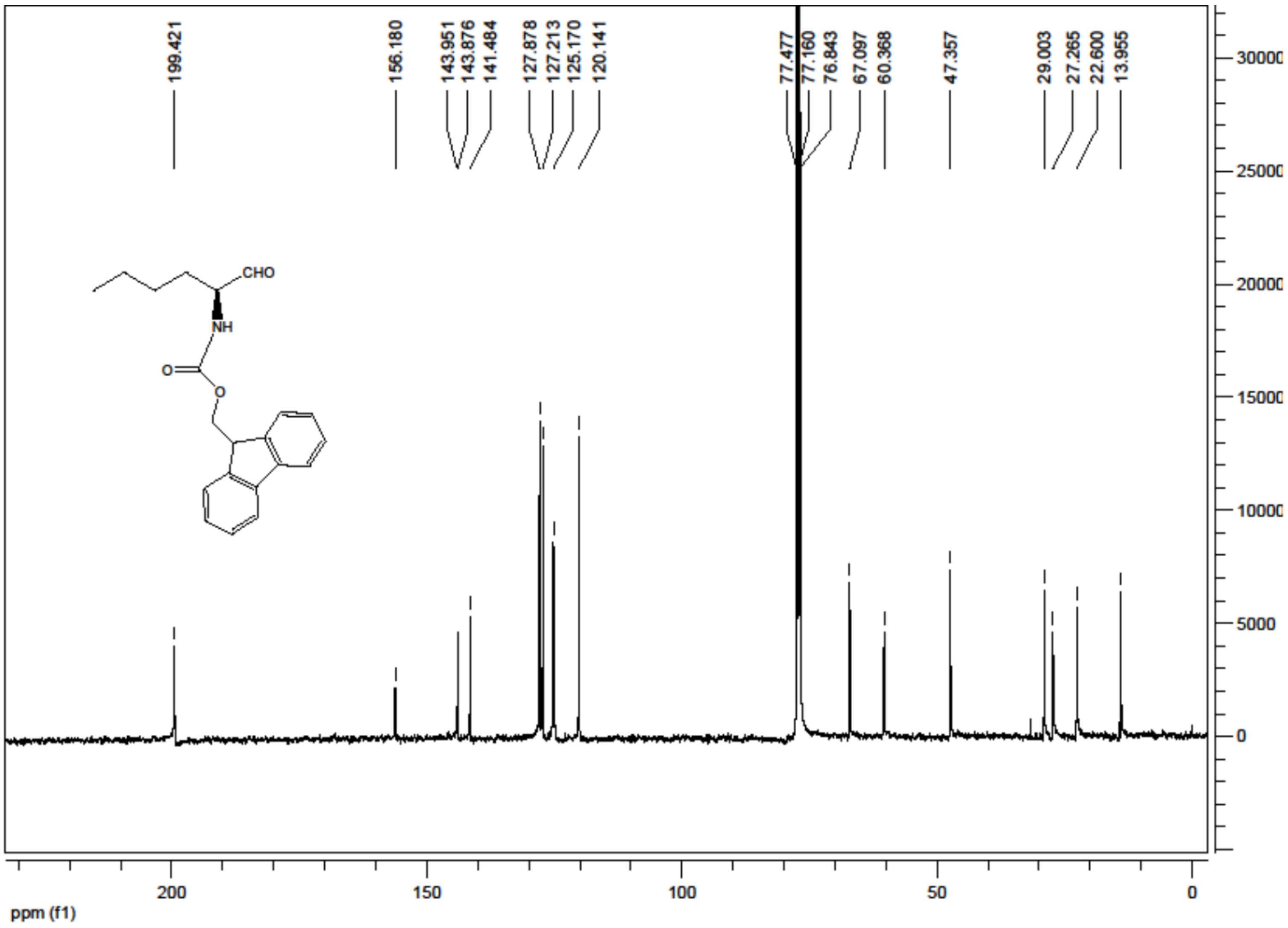

Figure S11. ^{13}C NMR spectrum of **7** ($CDCl_3$, 100 MHz).

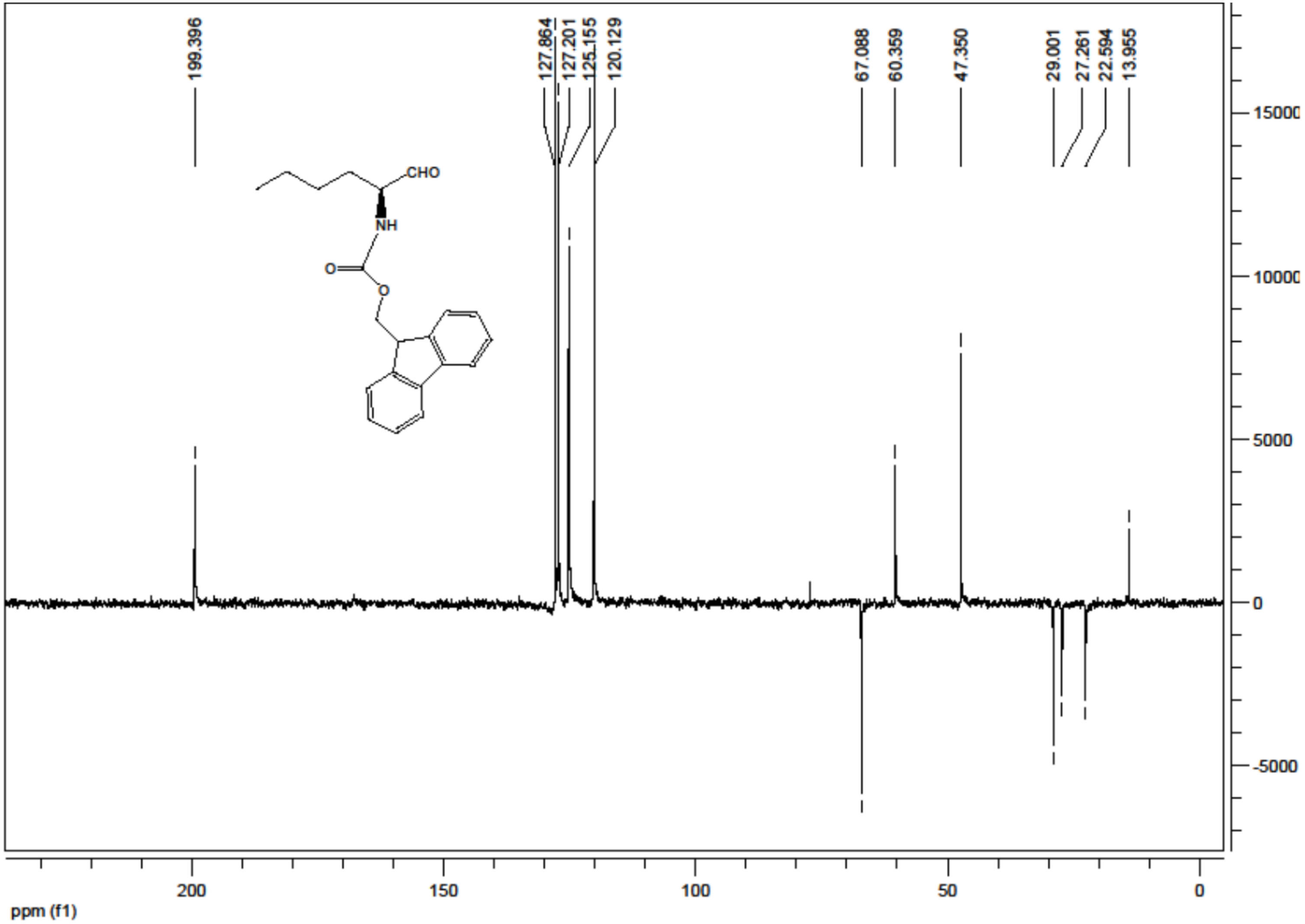

Figure S12. DEPT-135 spectrum of **7** ($CDCl_3$, 100 MHz).

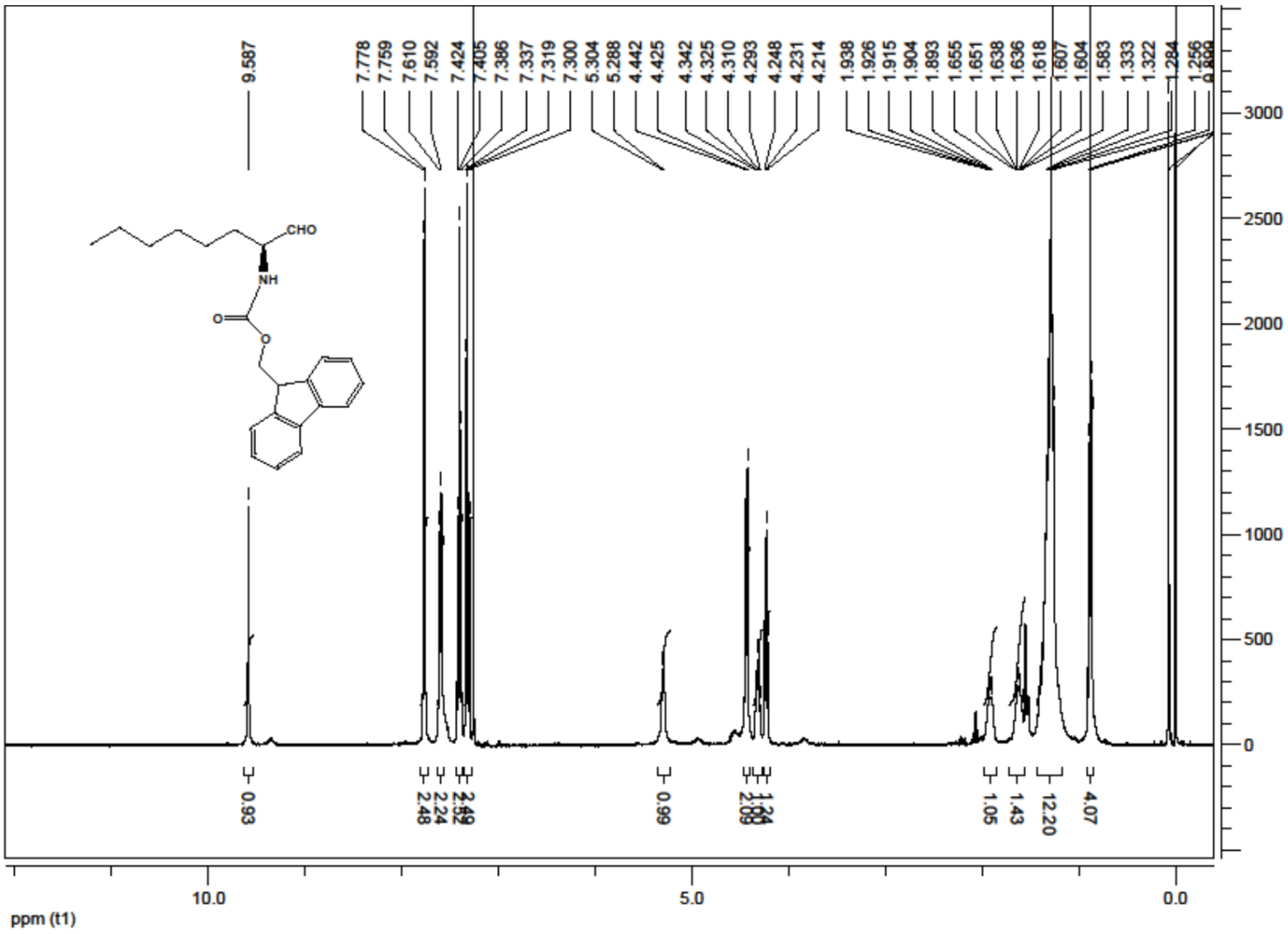

Figure S13. ^{1}H NMR spectrum of **8** ($CDCl_3$, 400 MHz).

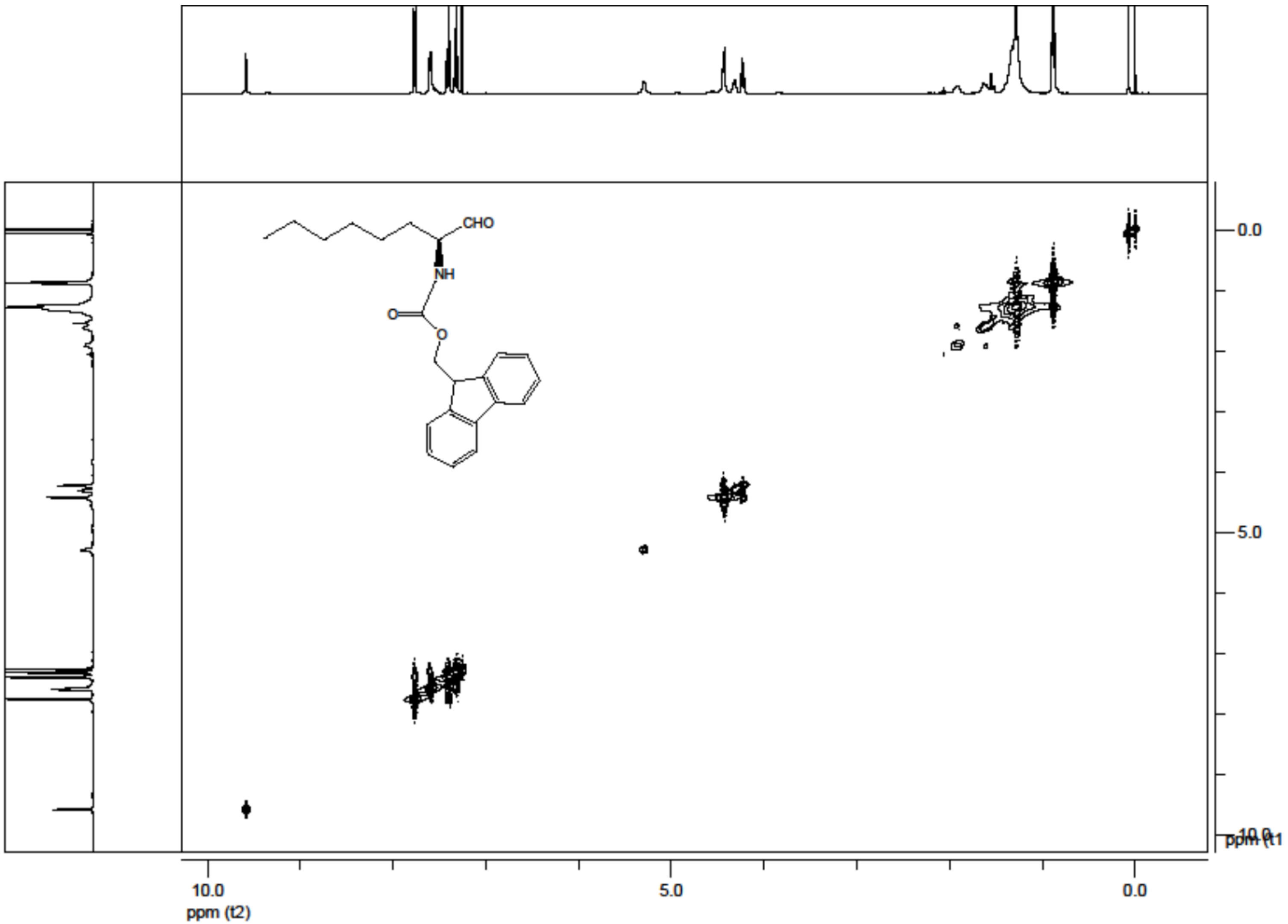

Figure S14. ^{1}H-^{1}H COSY spectrum of **8** in $CDCl_3$.

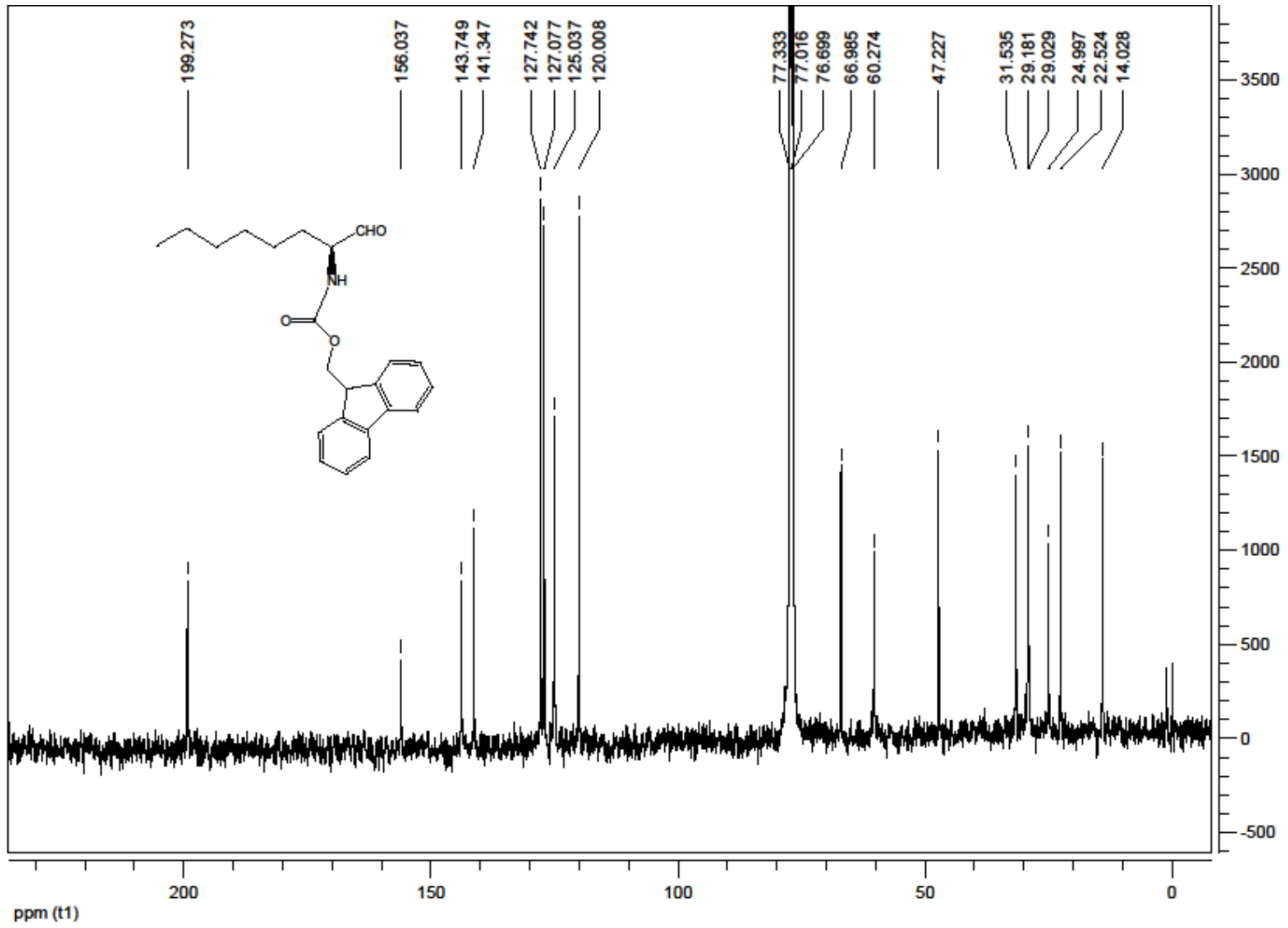

Figure S15. ^{13}C NMR spectrum of **8** ($CDCl_3$, 100 MHz).

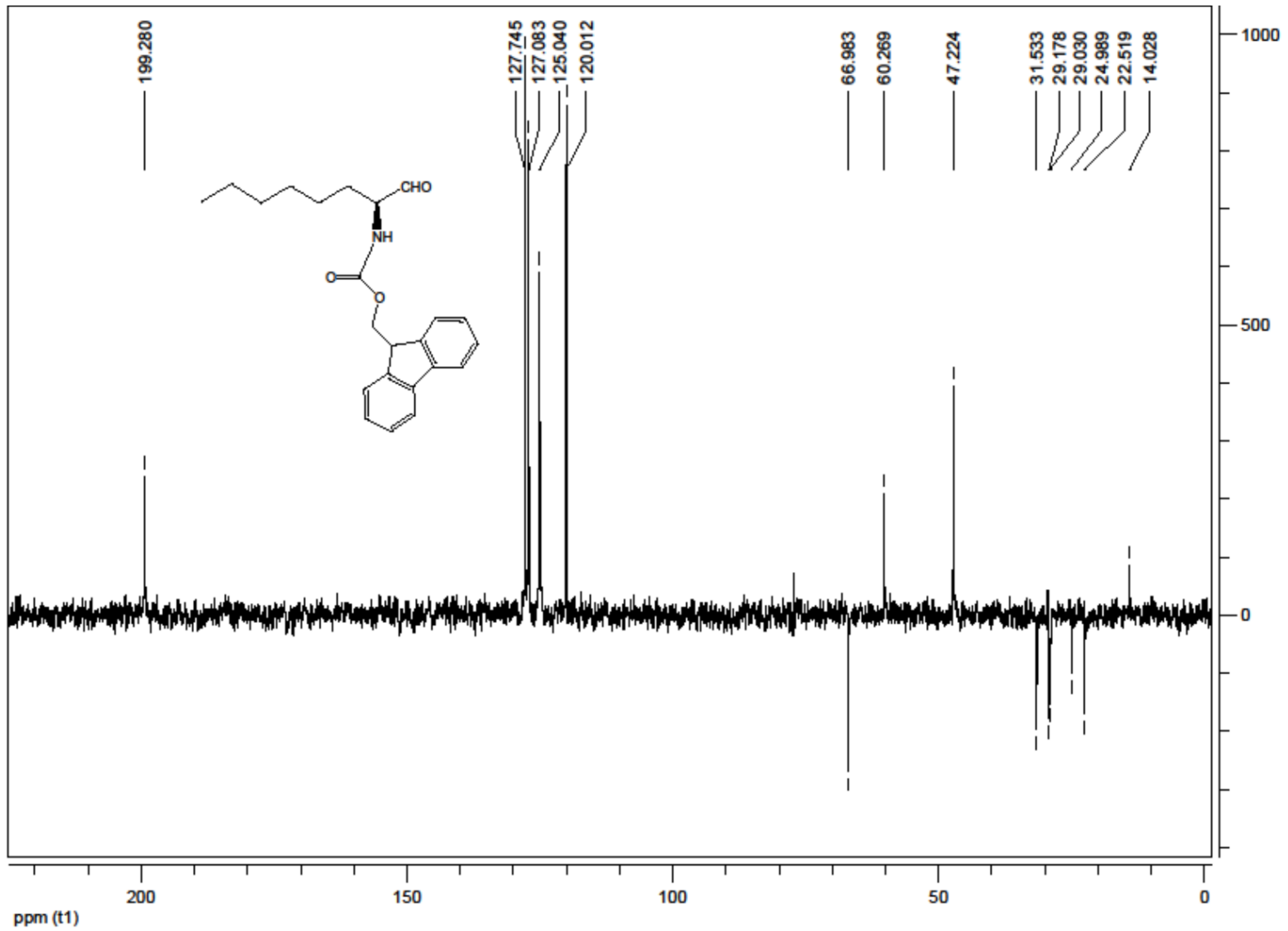

Figure S16. DEPT-135 spectrum of **8** ($CDCl_3$, 100 MHz).

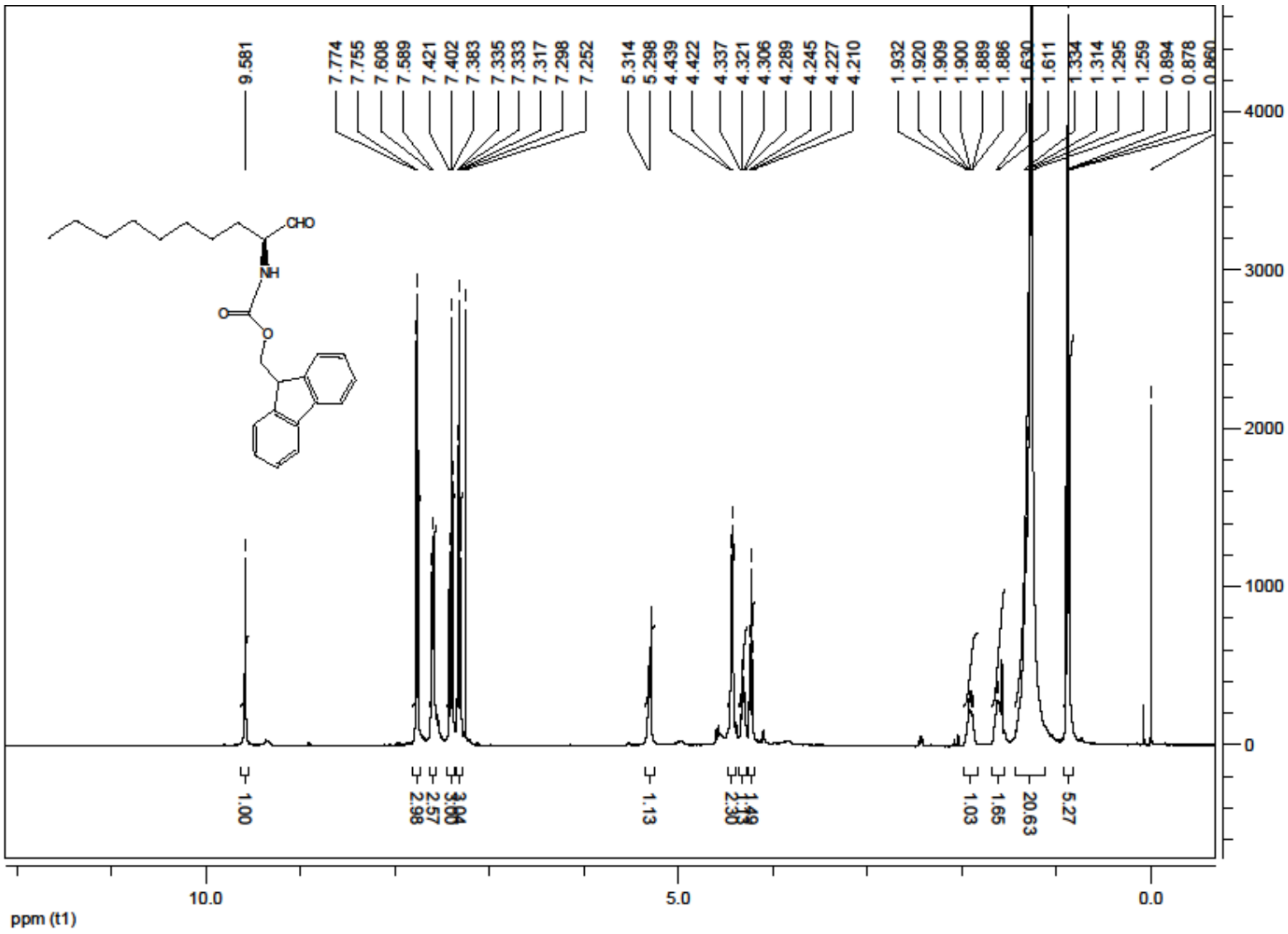

Figure S17. ^{1}H NMR spectrum of **9** ($CDCl_3$, 400 MHz).

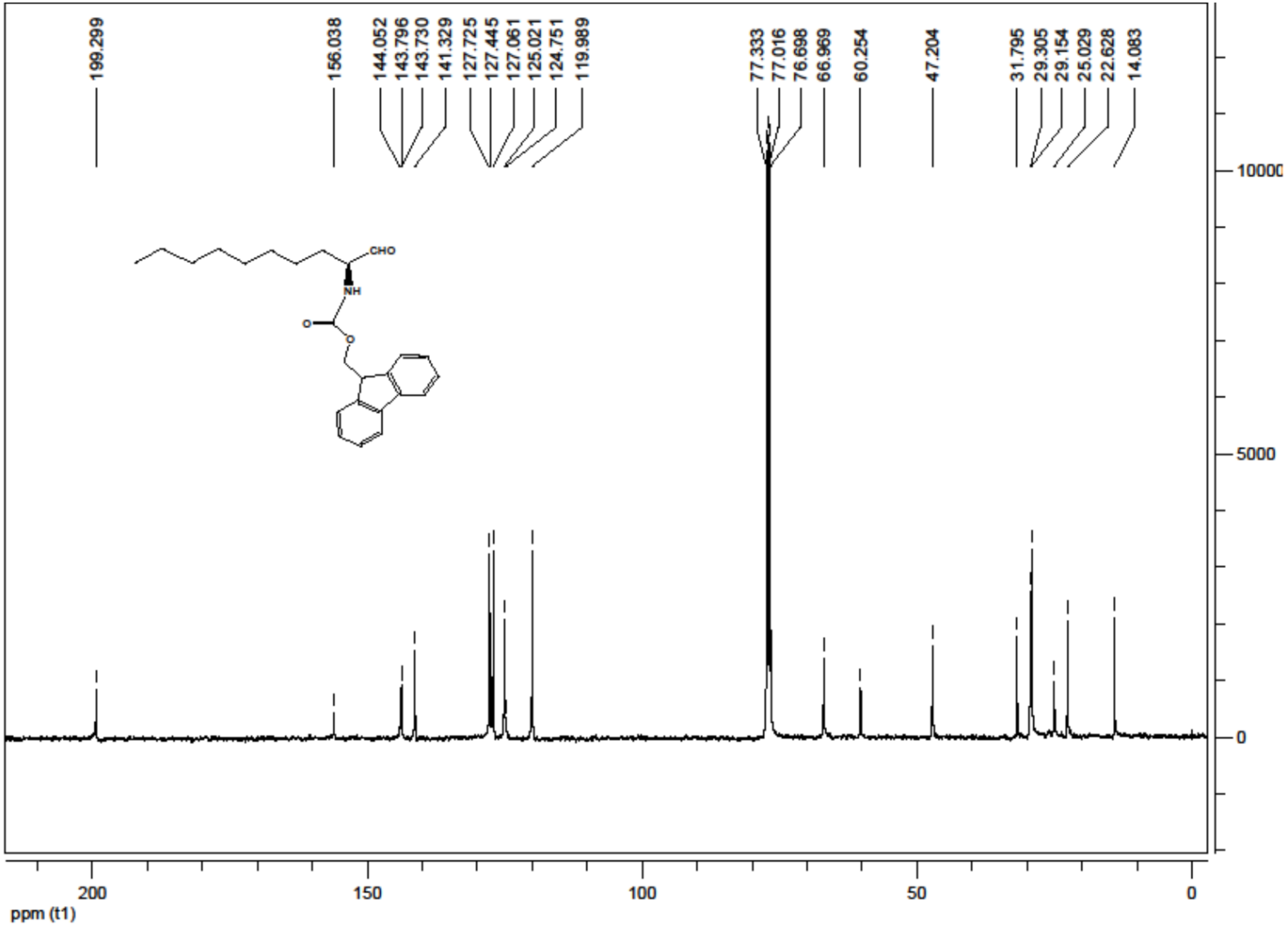

Figure S18. ^{13}C NMR spectrum of **9** ($CDCl_3$, 100 MHz).

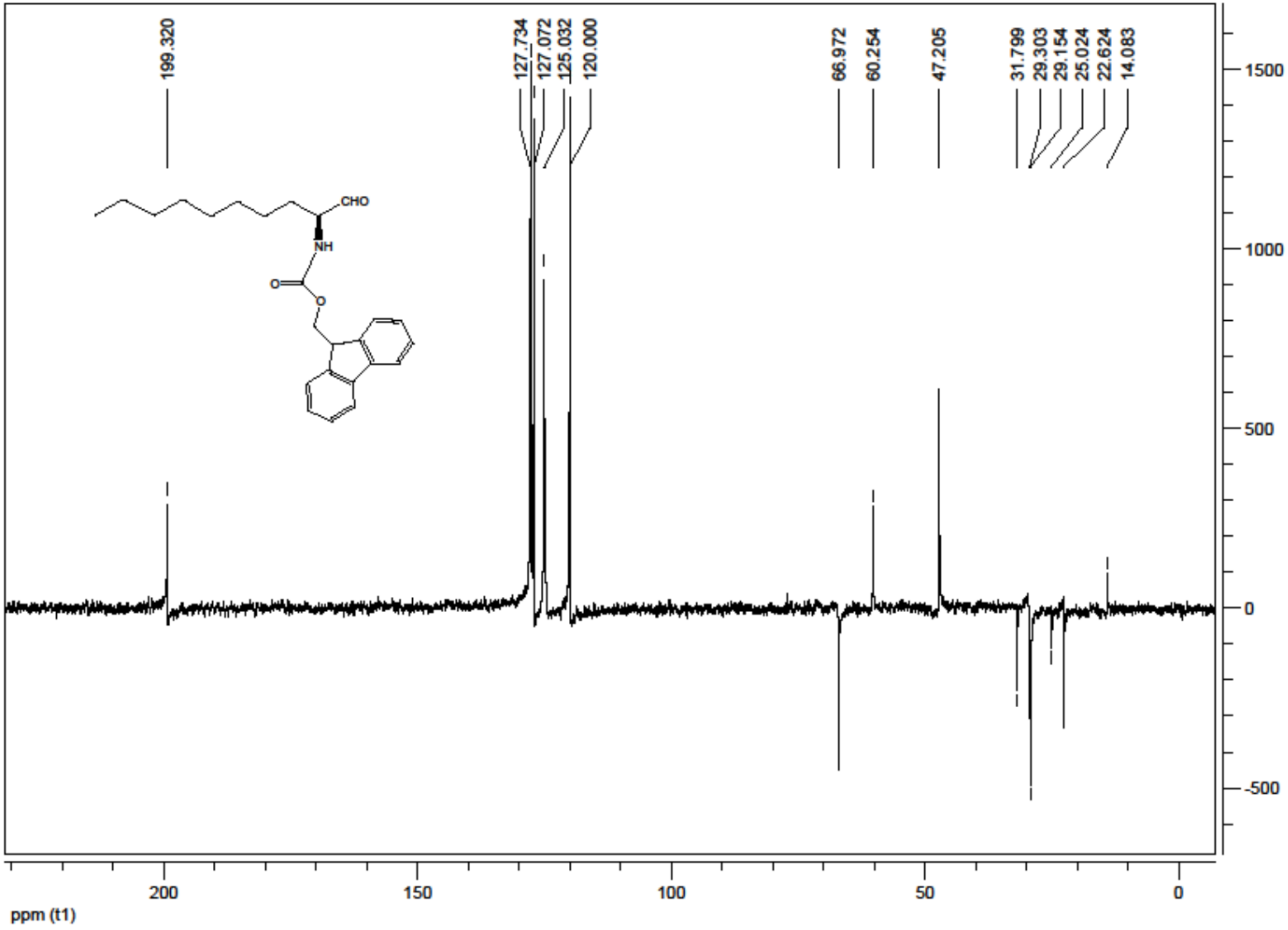

s

Figure S19. DEPT-135 spectrum of **9** ($CDCl_3$, 100 MHz).

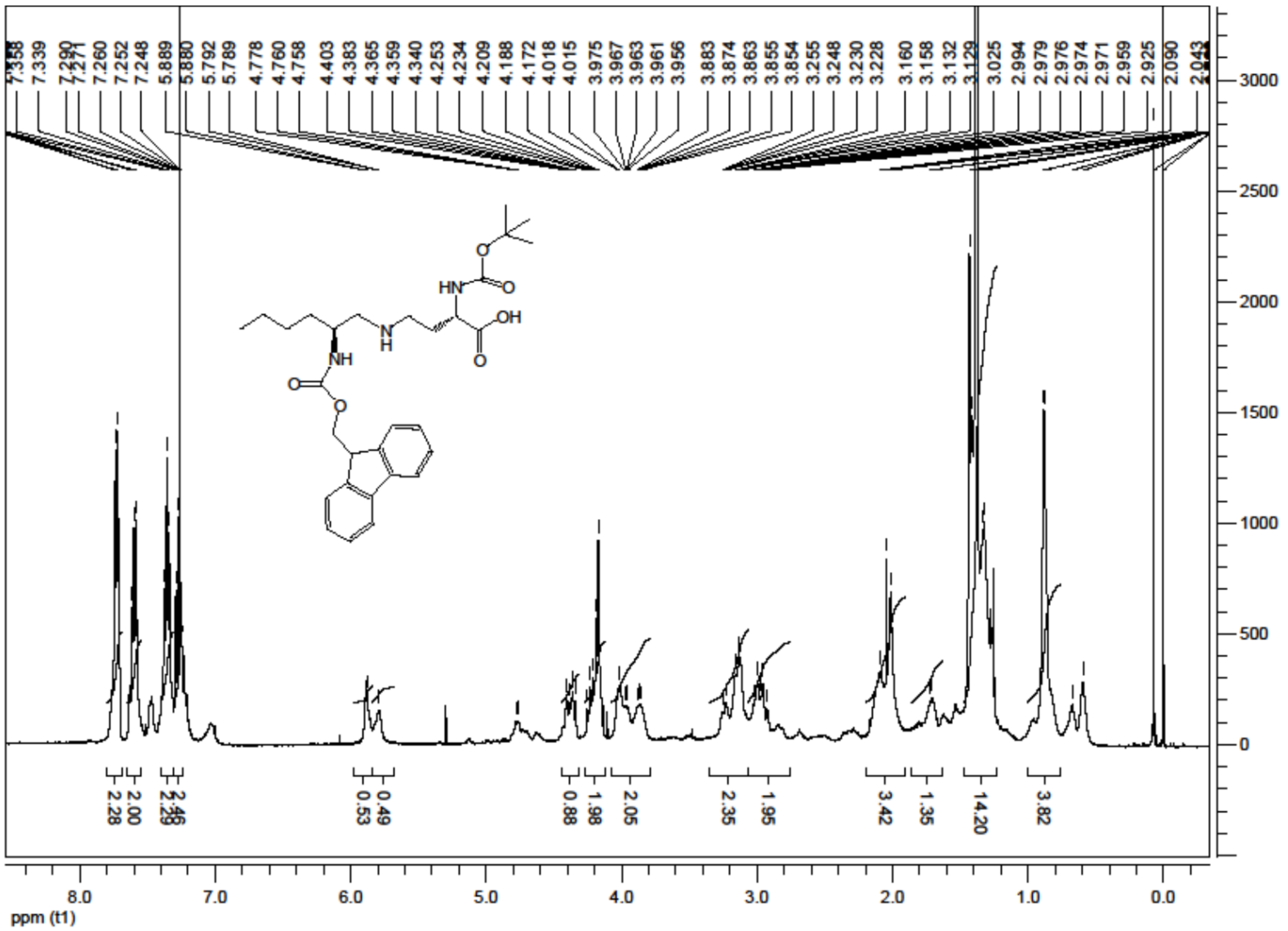

Figure S20. ^{1}H NMR spectrum of **11** ($CDCl_3$, 400 MHz).

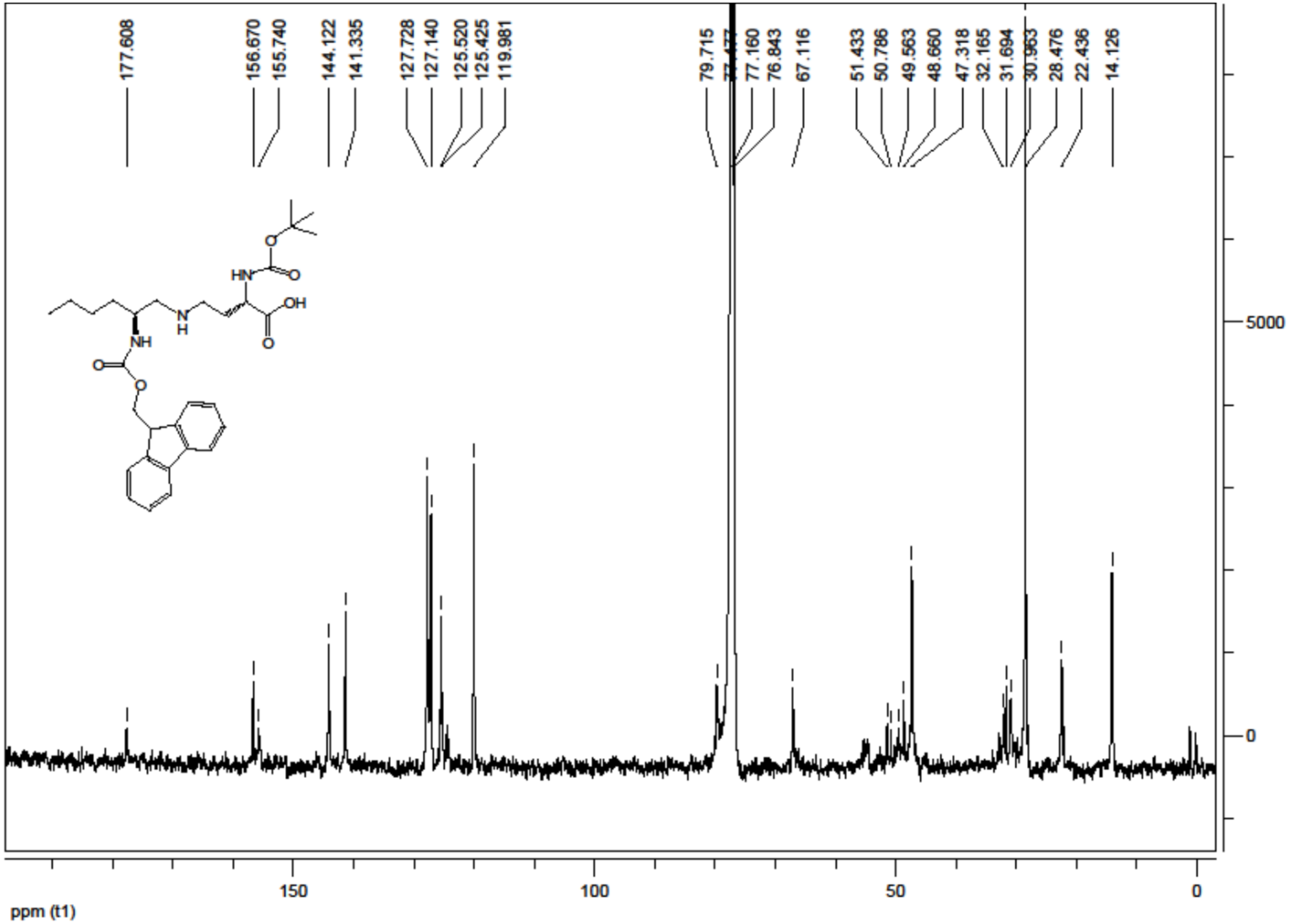

Figure S21. ^{13}C NMR spectrum of **11** ($CDCl_3$, 100 MHz).

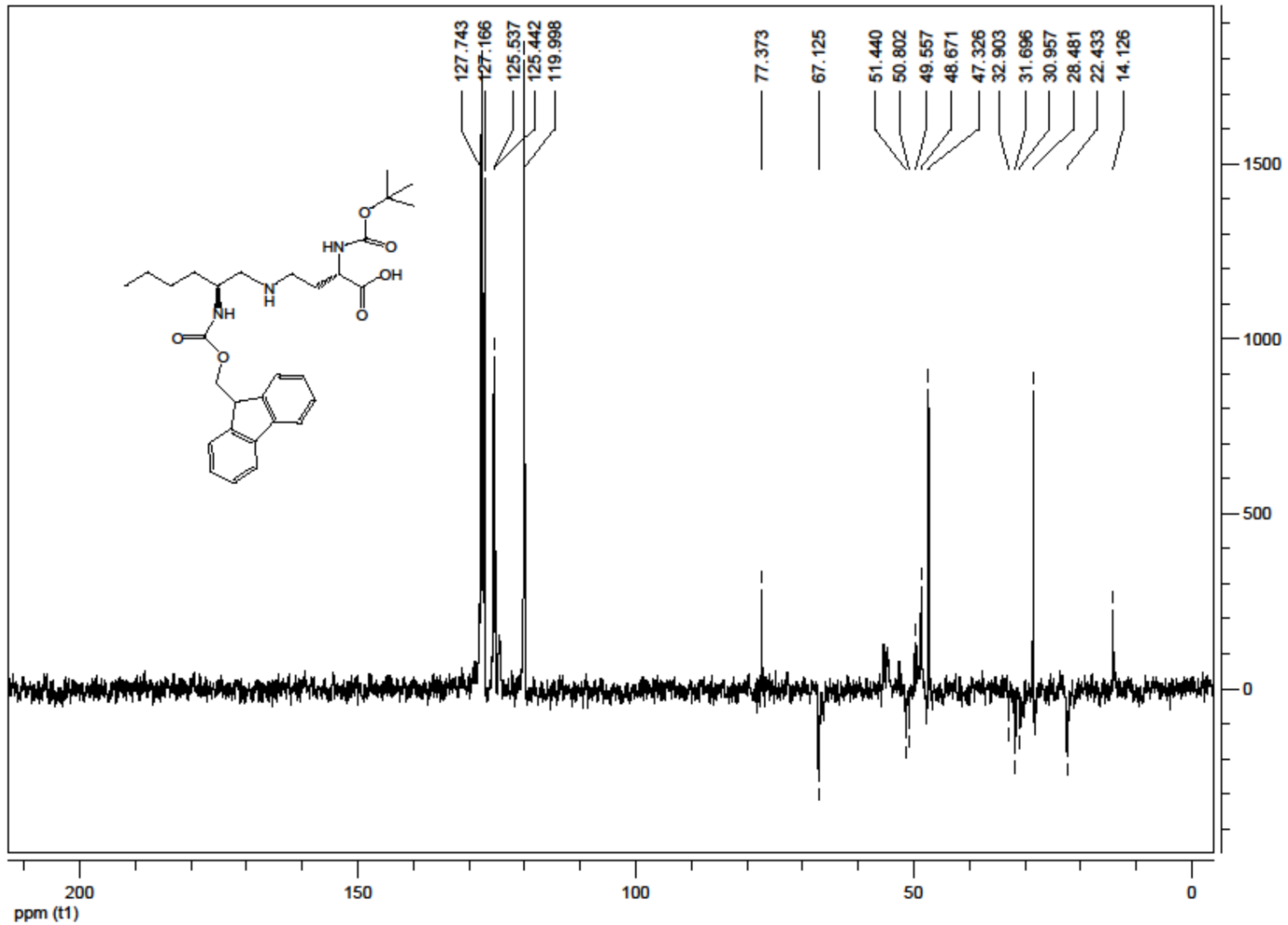

Figure S22. DEPT-135 spectrum of **11** ($CDCl_3$, 100 MHz).

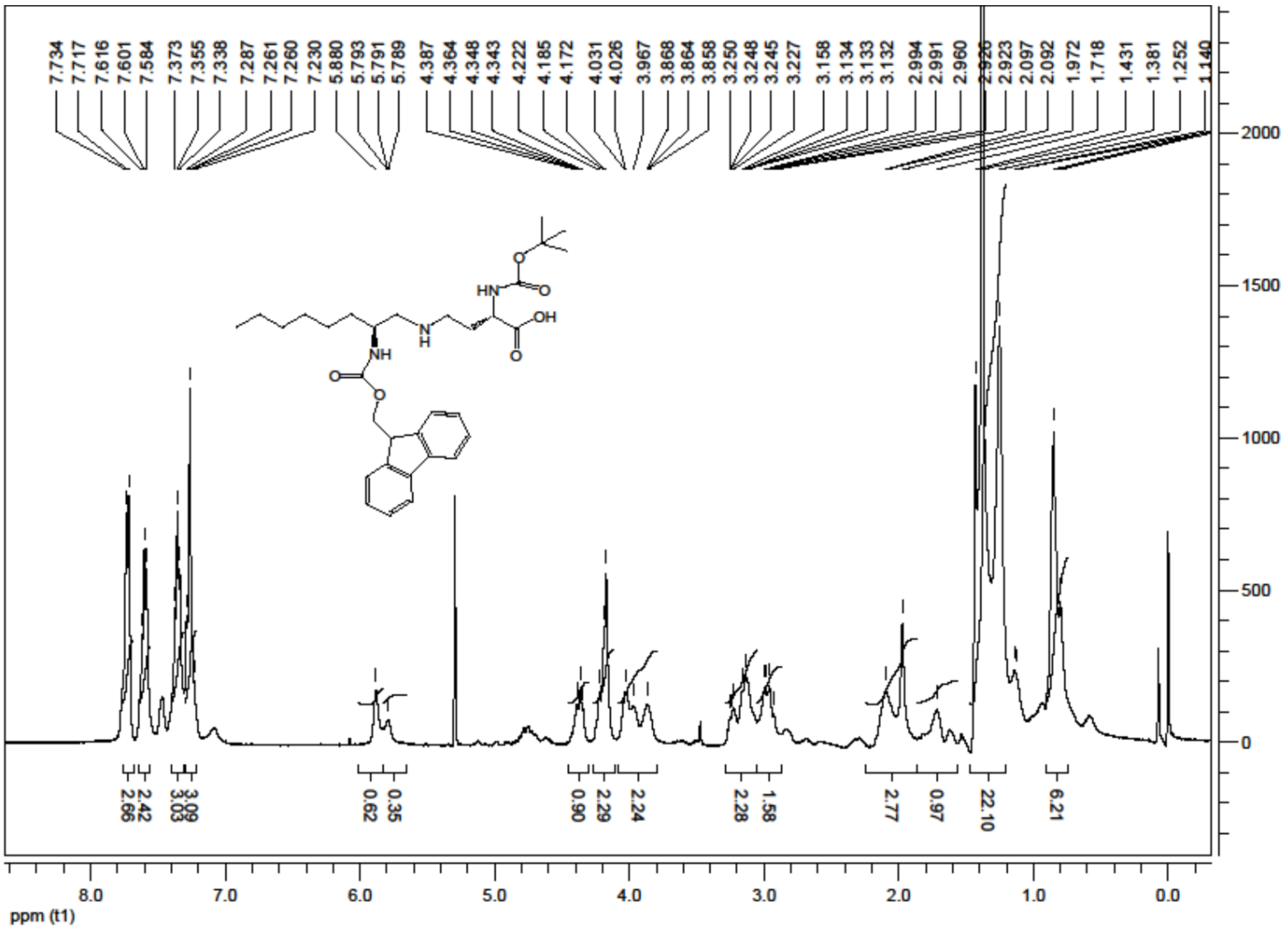

Figure S23. ^{1}H NMR spectrum of **12** ($CDCl_3$, 400 MHz).

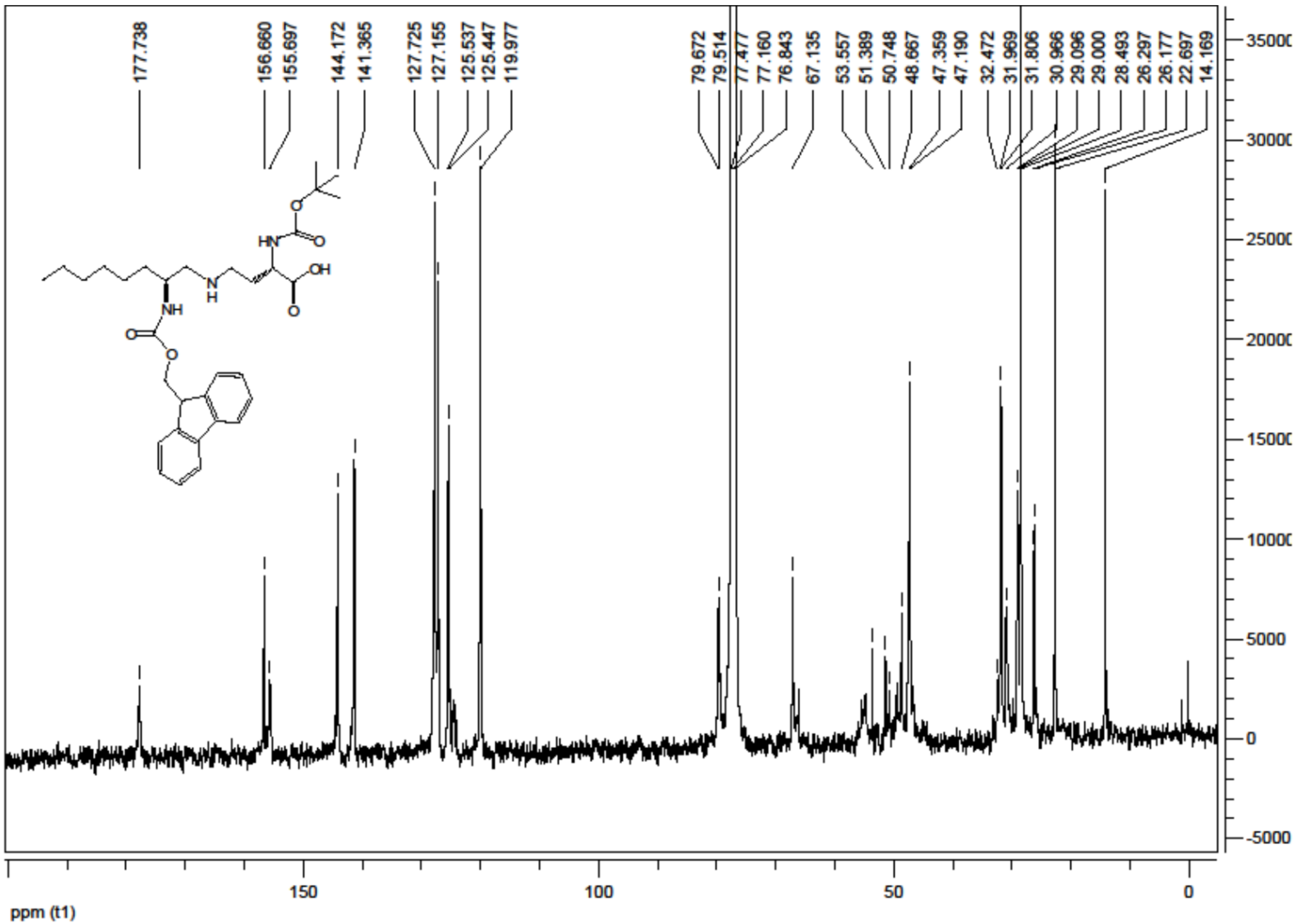

Figure S24. ^{13}C NMR spectrum of **12** ($CDCl_3$, 100 MHz).

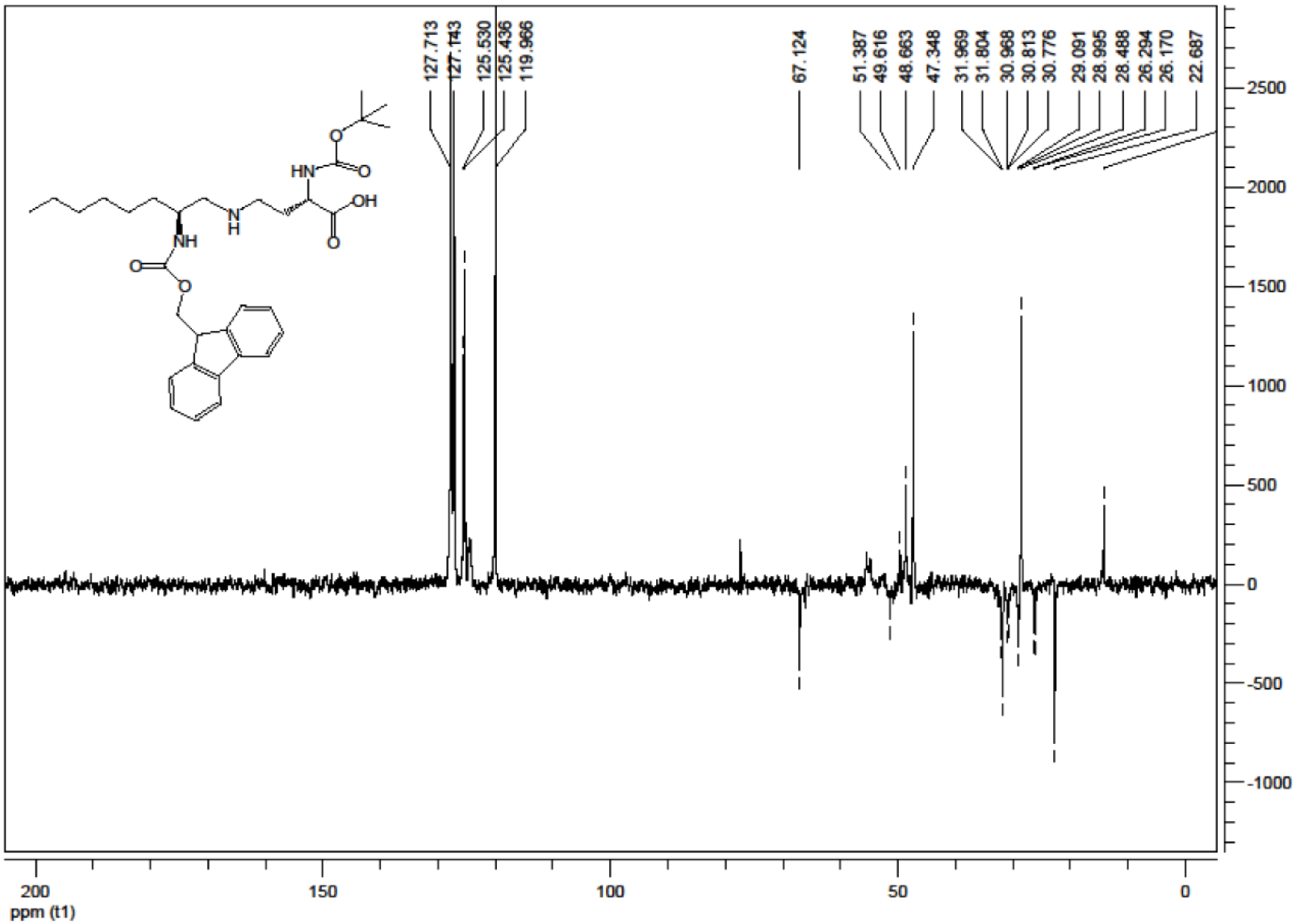

Figure S25. DEPT-135 spectrum of **12** ($CDCl_3$, 100 MHz).

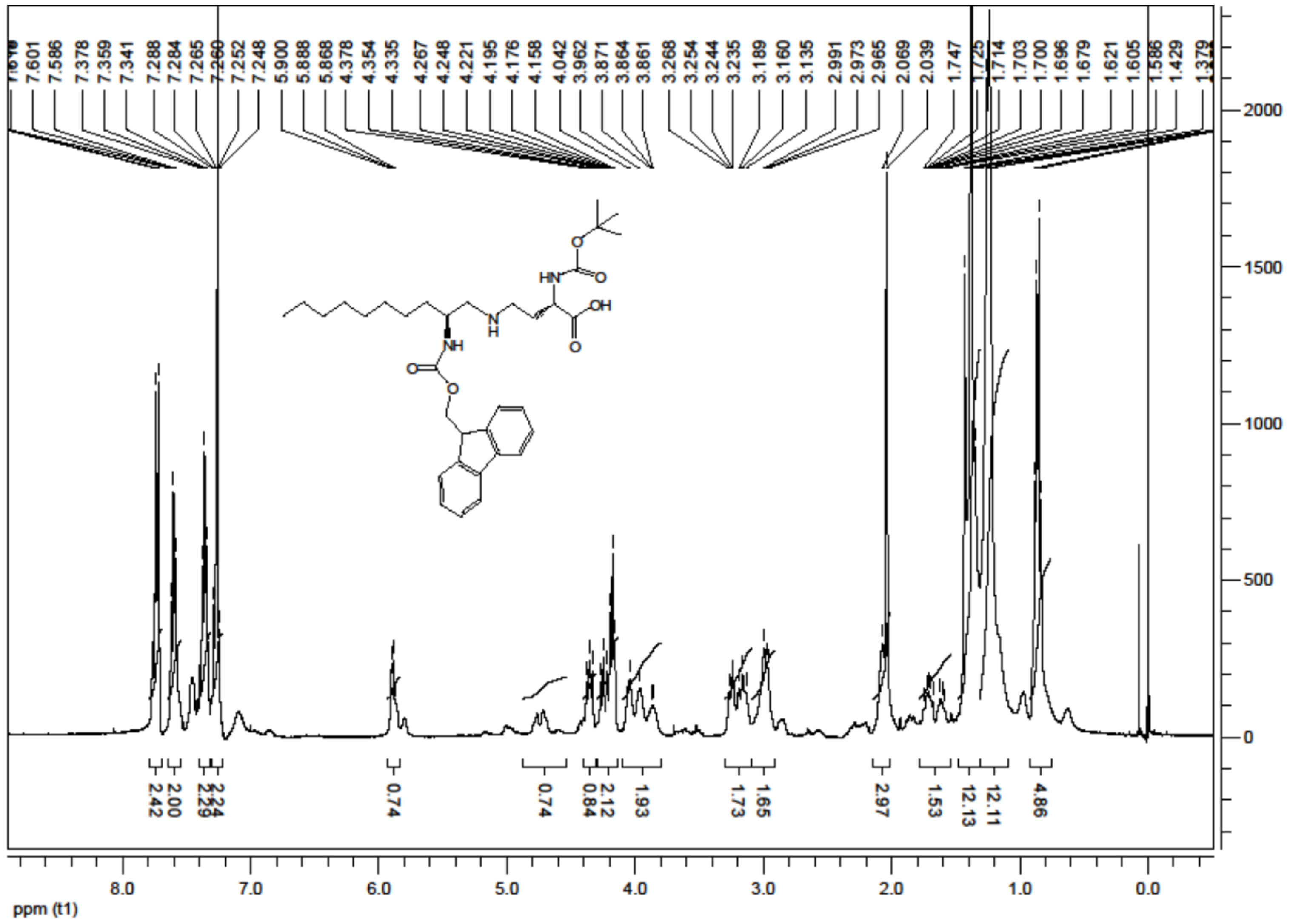

Figure S26. ^{1}H NMR spectrum of **13** ($CDCl_3$, 400 MHz).

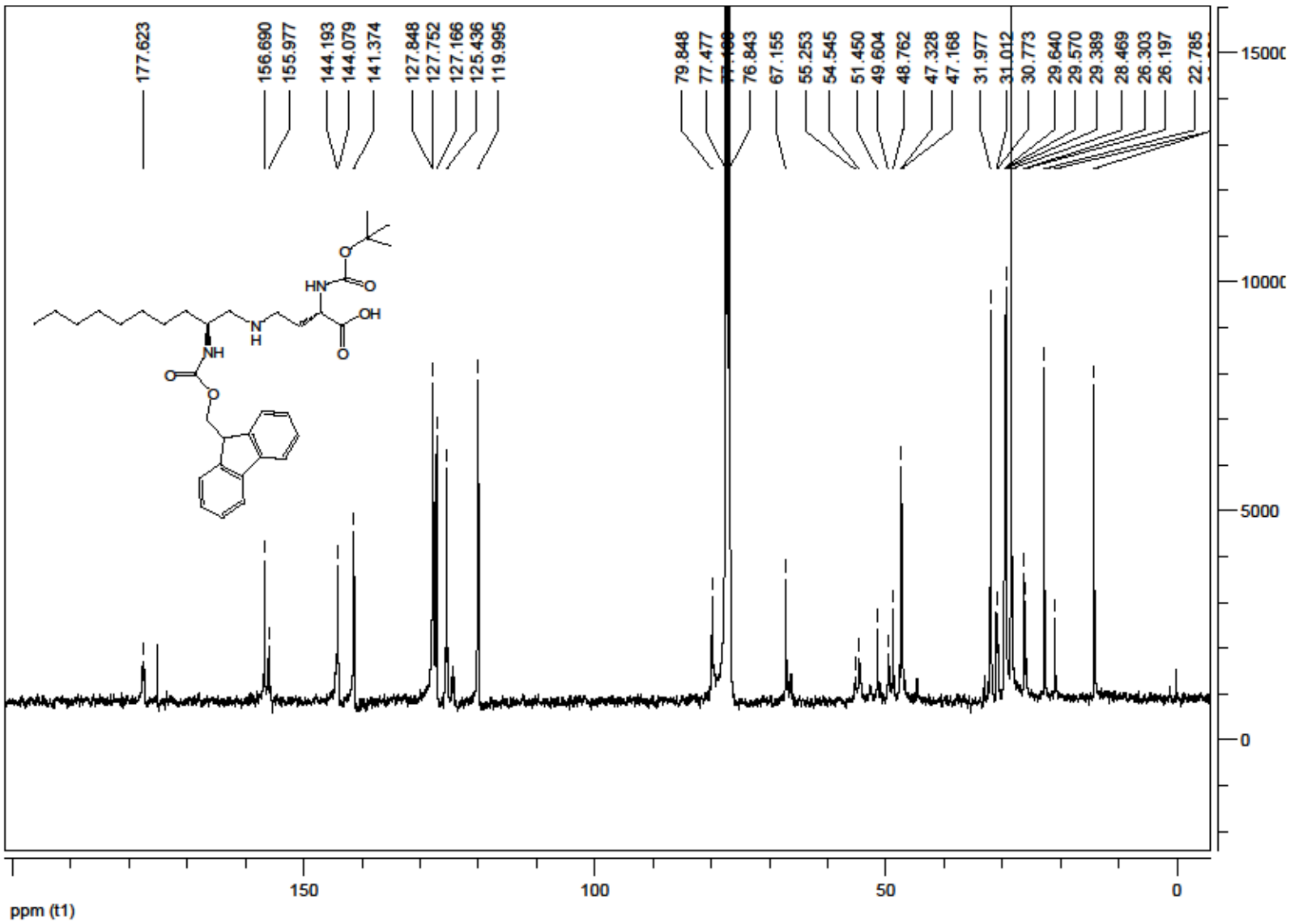

Figure S27. ^{13}C NMR spectrum of **13** ($CDCl_3$, 100 MHz).

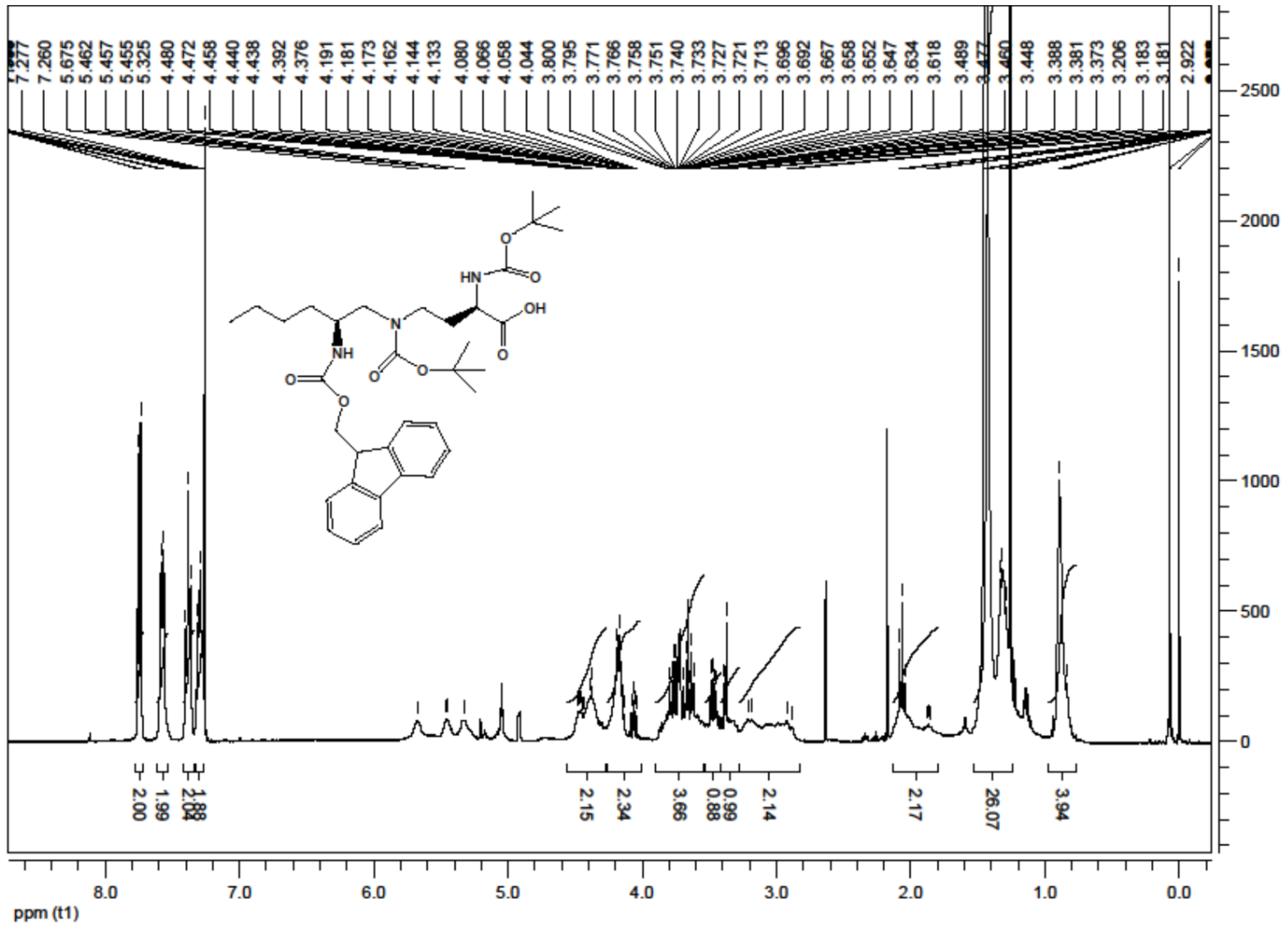

Figure S28. ^1H NMR spectrum of **14** ($CDCl_3$, 400 MHz).

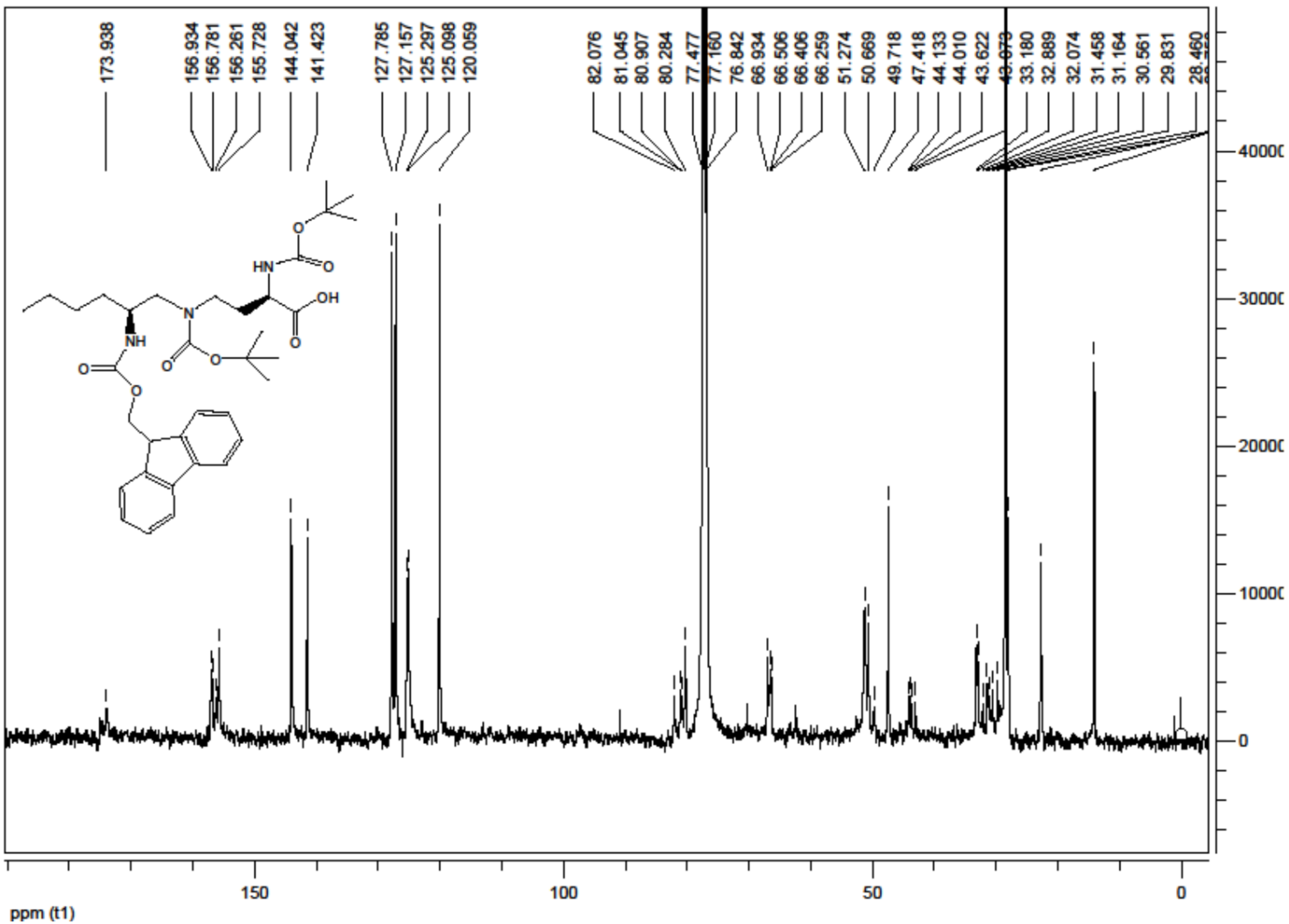

Figure S29. ^{13}C NMR spectrum of **14** ($CDCl_3$, 100 MHz).

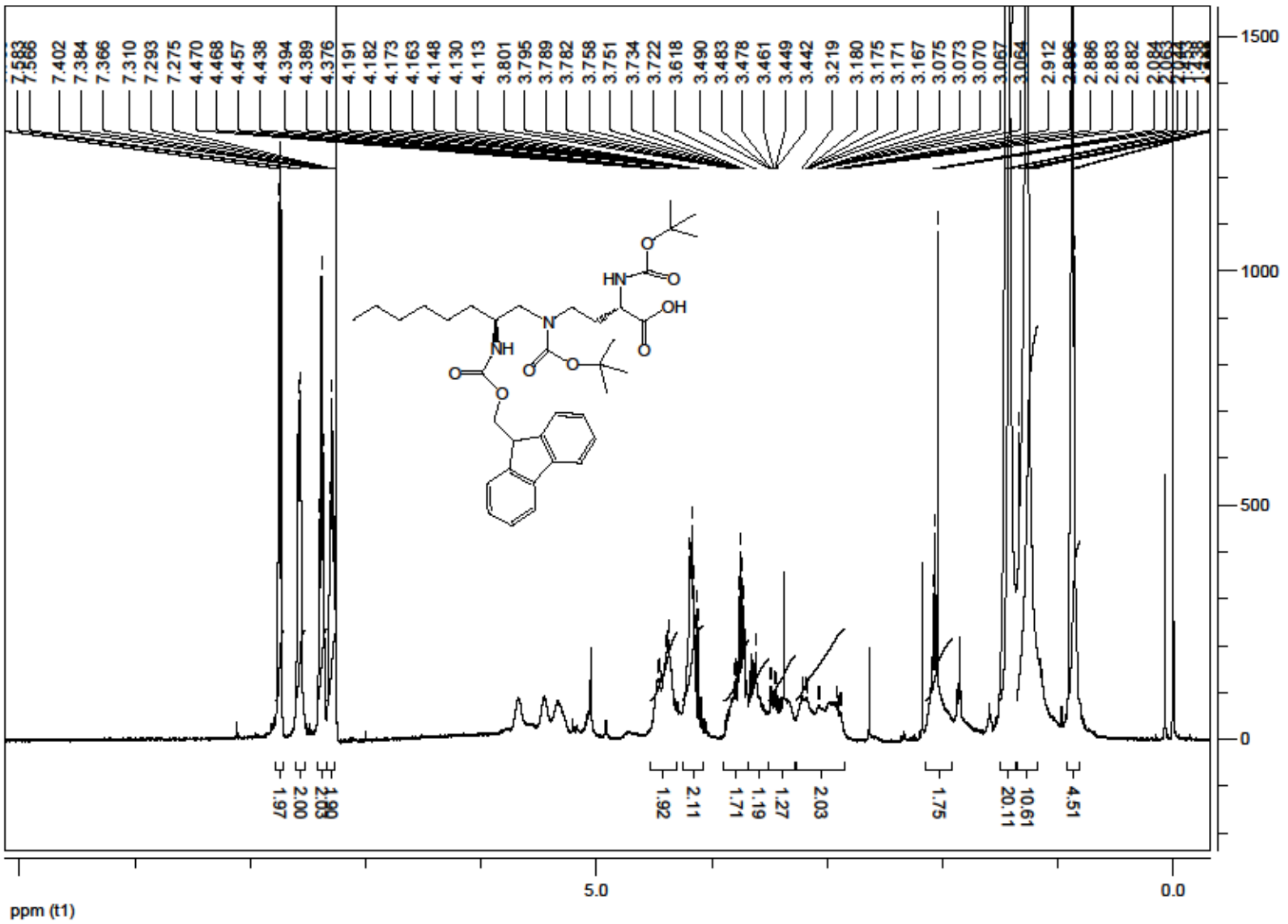

Figure S30. ^{1}H NMR spectrum of **15** ($CDCl_3$, 400 MHz).

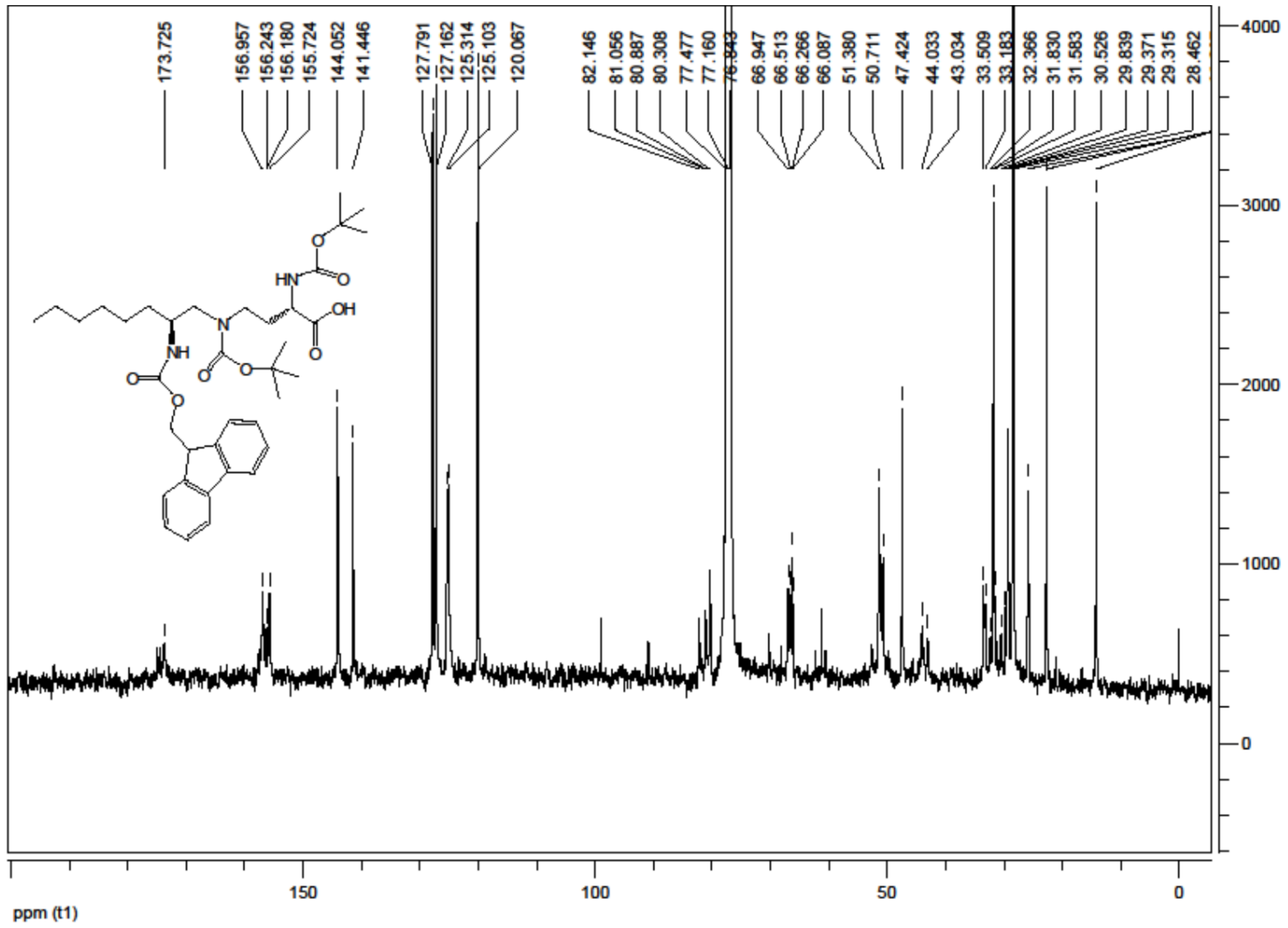

Figure S31. ^{13}C NMR spectrum of **15** ($CDCl_3$, 100 MHz).

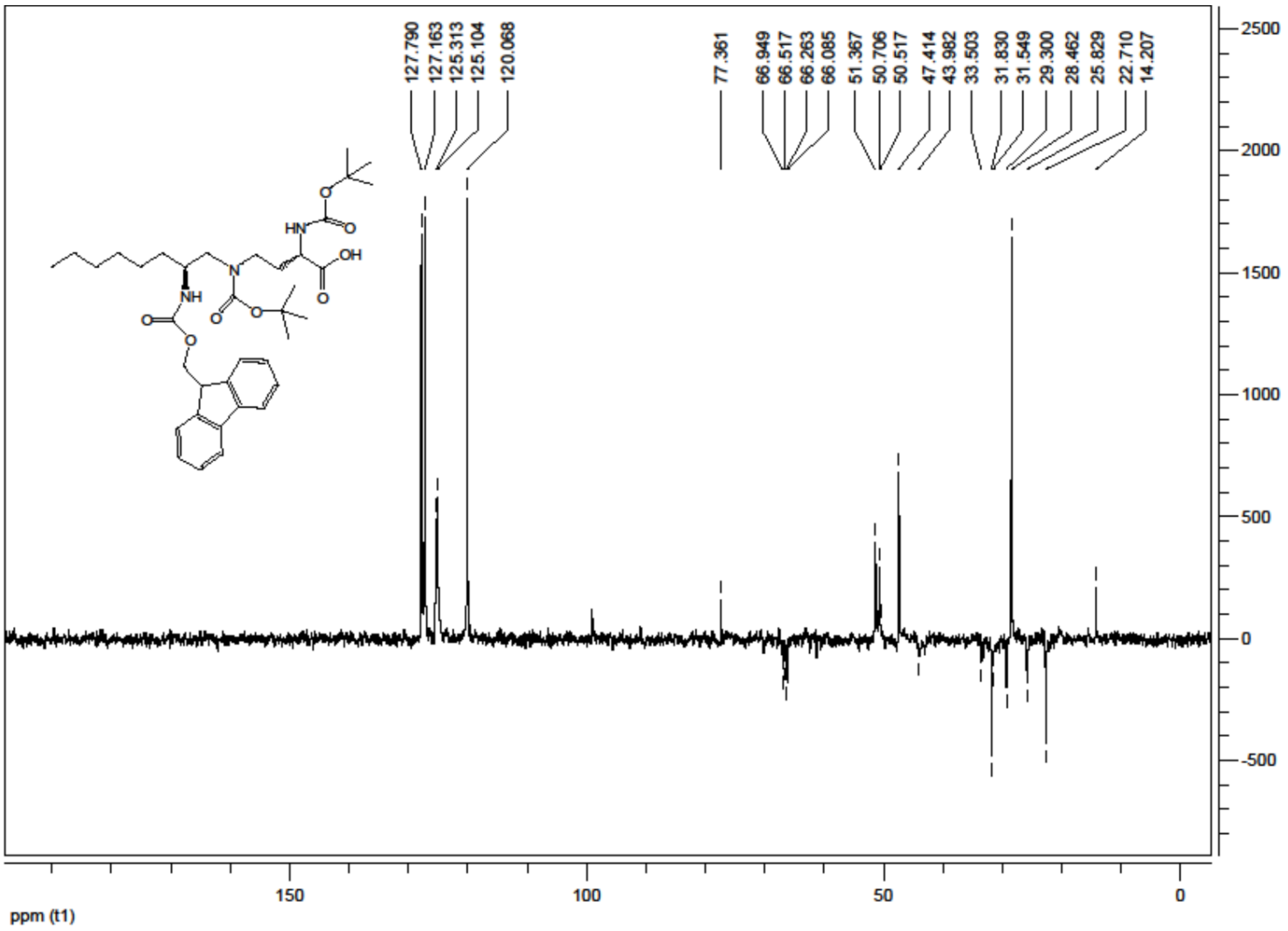

Figure S32. DEPT-135 spectrum of **15** ($CDCl_3$, 100 MHz).

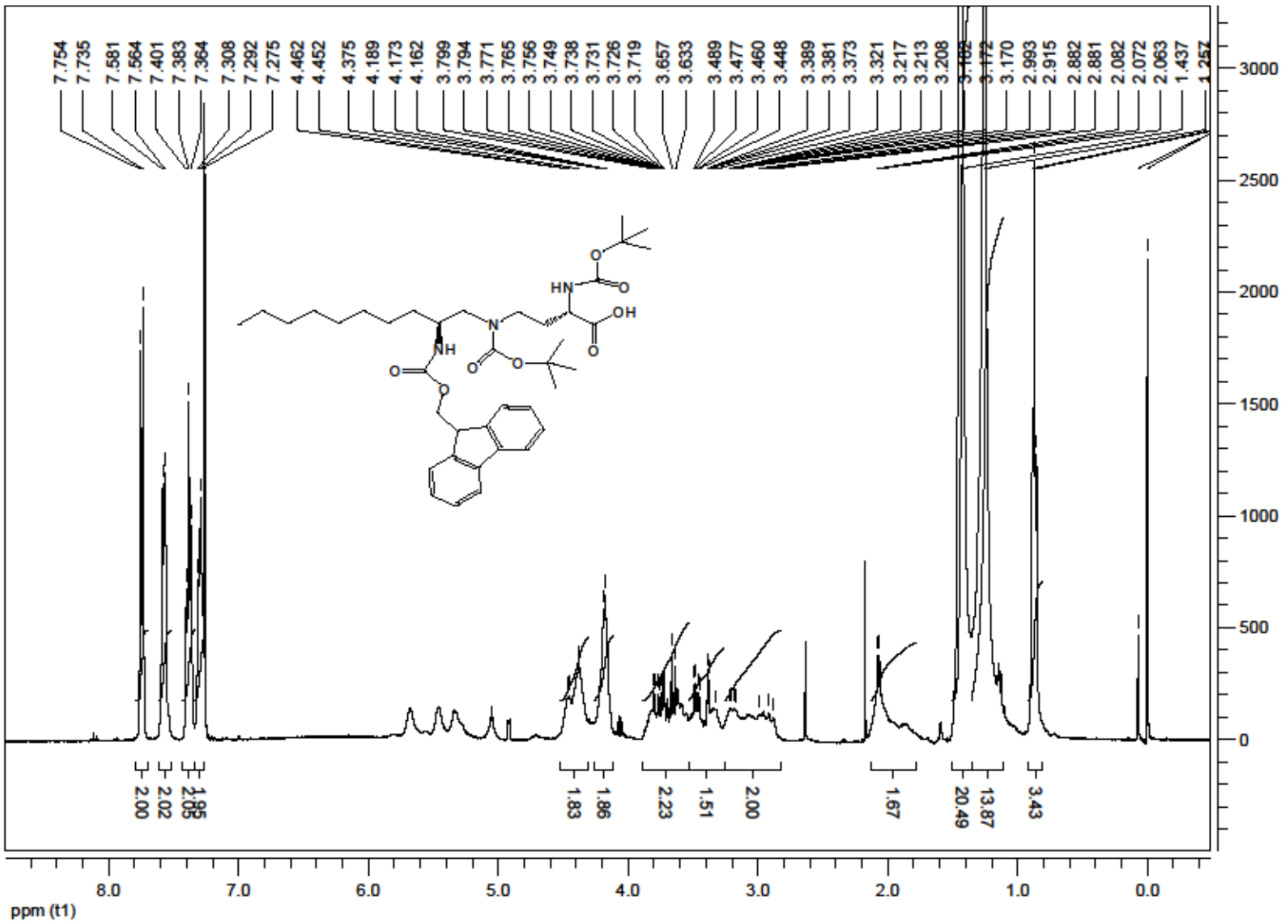

Figure S33. ^{1}H NMR spectrum of **16** ($CDCl_3$, 400 MHz).

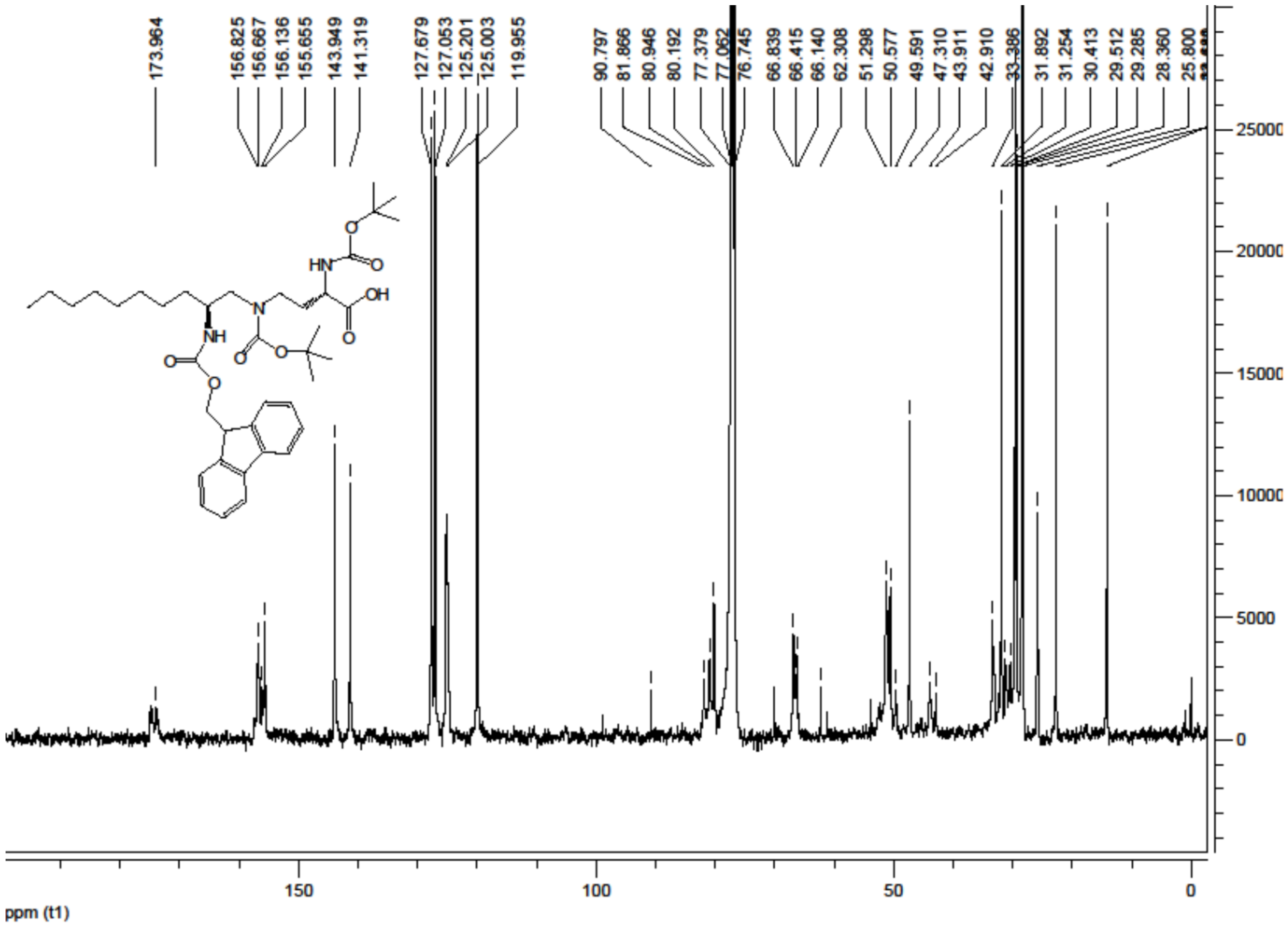

Figure S34. ^{13}C NMR spectrum of **16** ($CDCl_3$, 100 MHz).

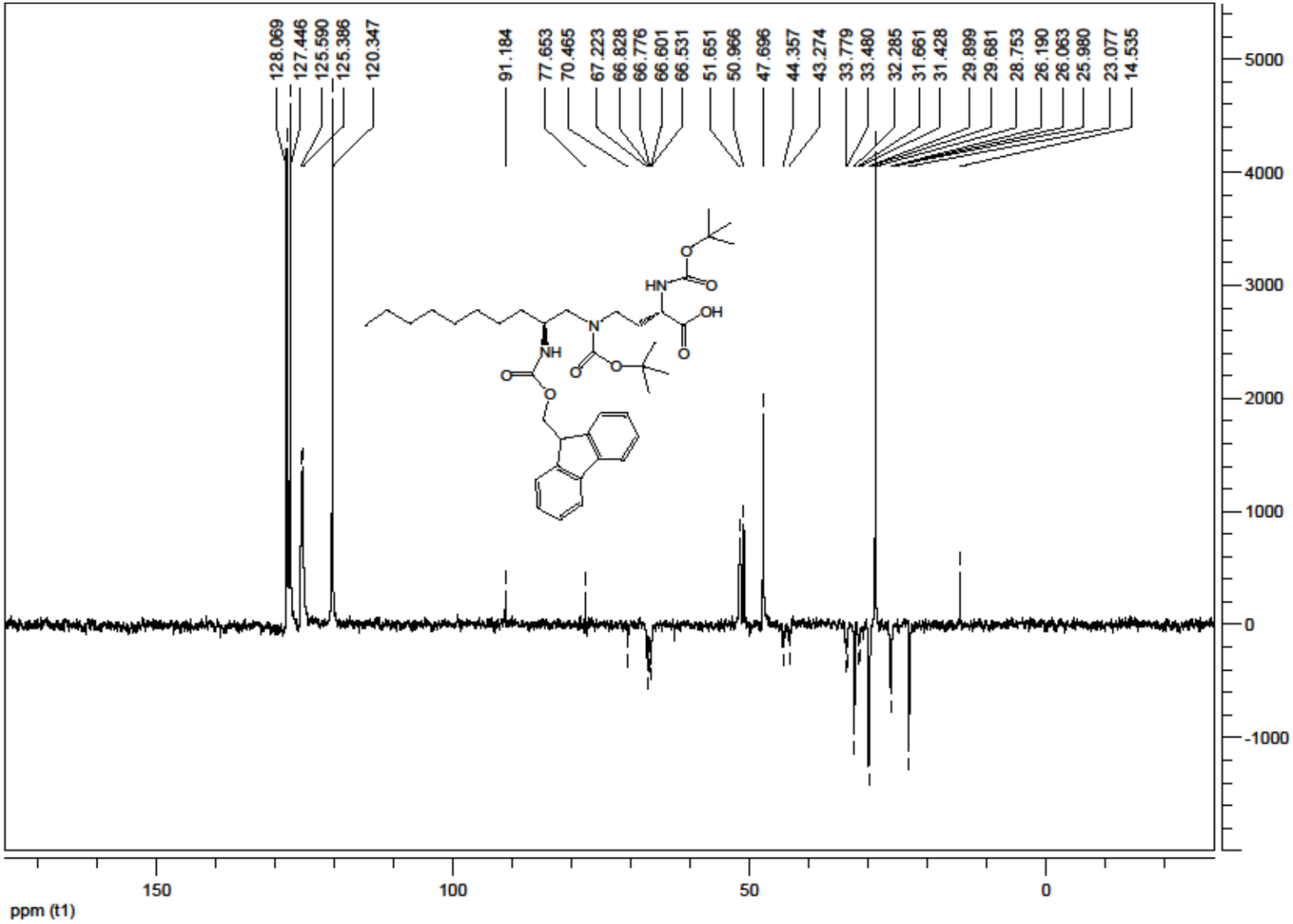

Figure S35. DEPT-135 spectrum of **16** ($CDCl_3$, 100 MHz).

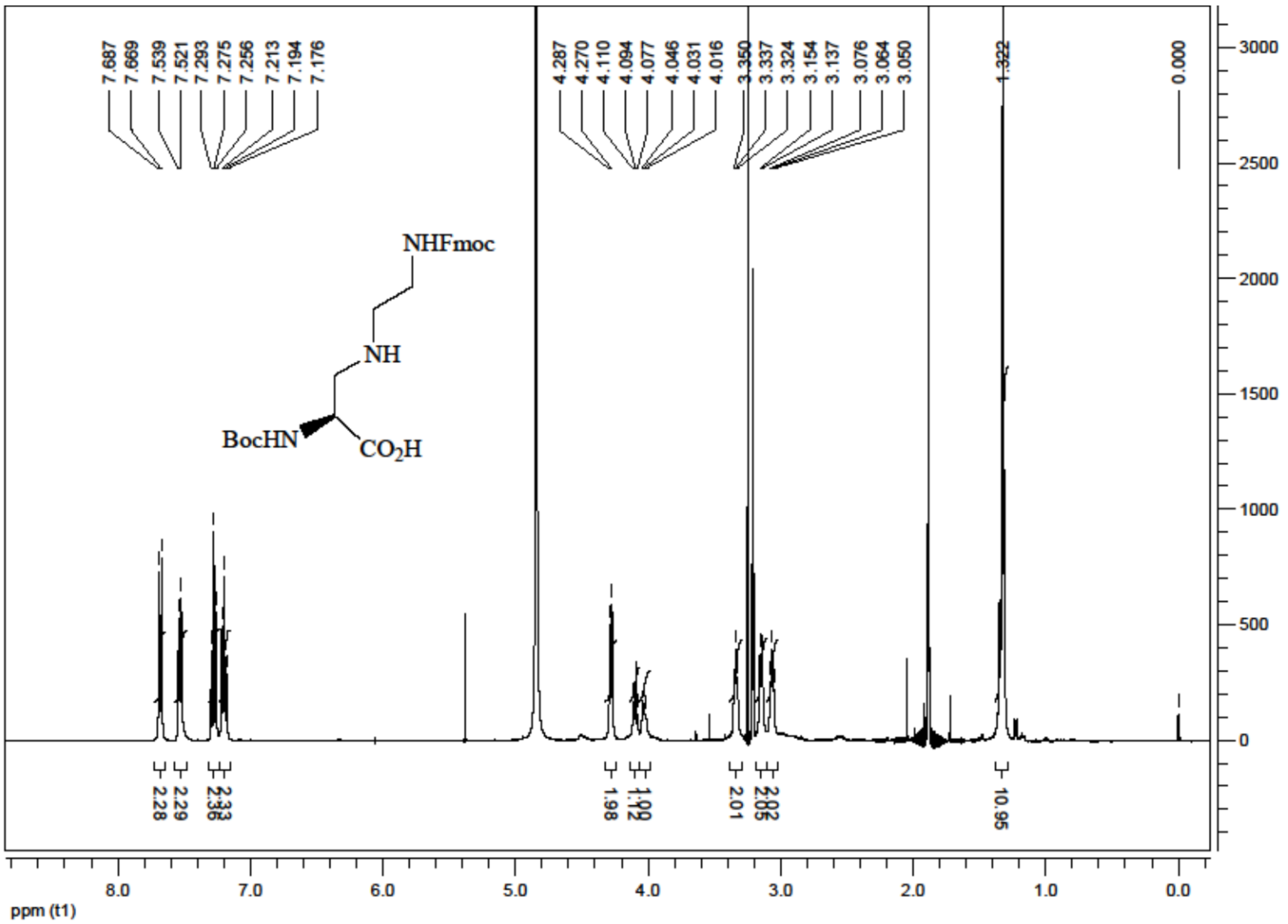

Figure S36. ^{1}H NMR spectrum of **22** (CD_3OD, 400 MHz).

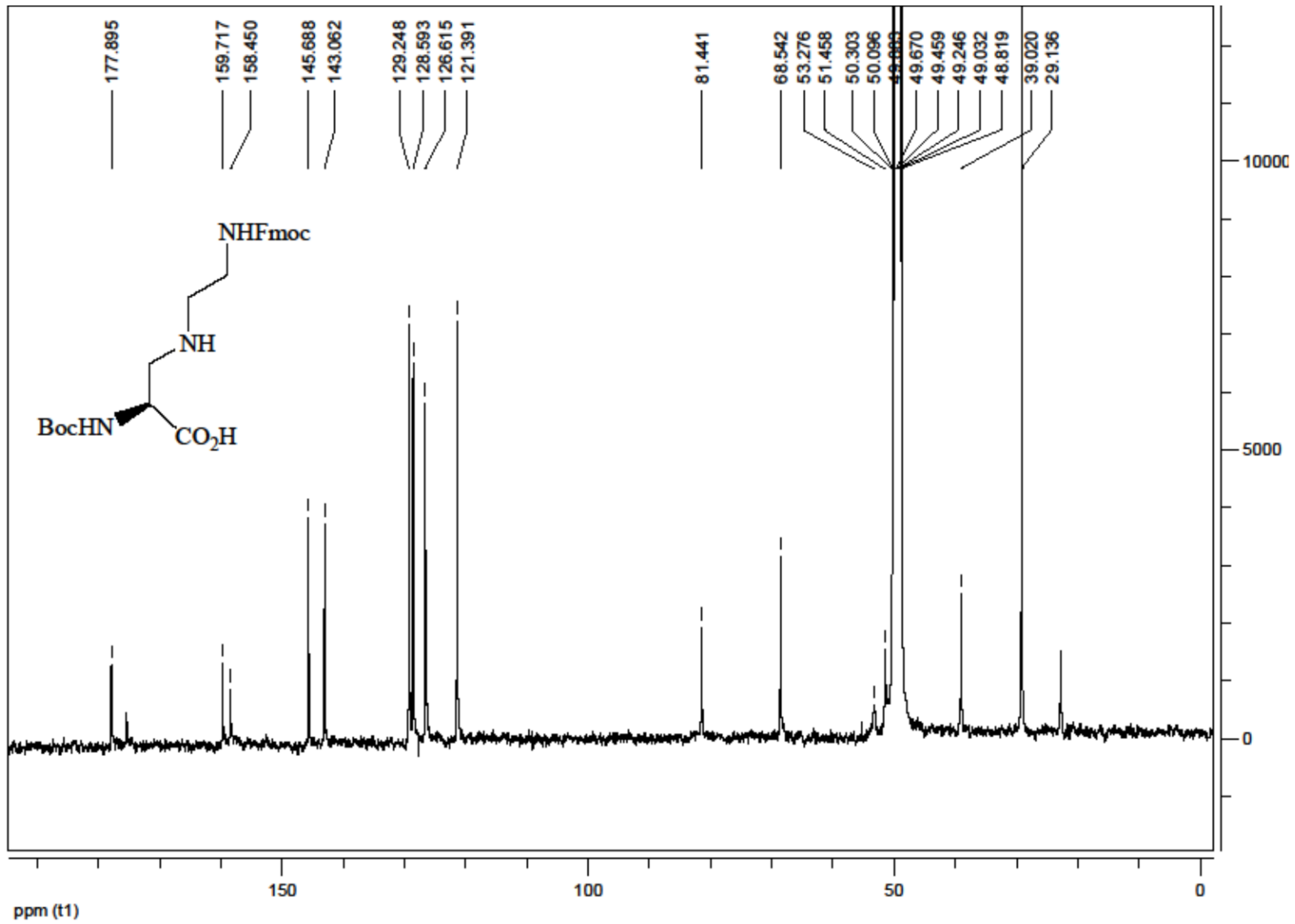

Figure S37. ^{13}C NMR spectrum of **22** (CD_3OD, 100 MHz).

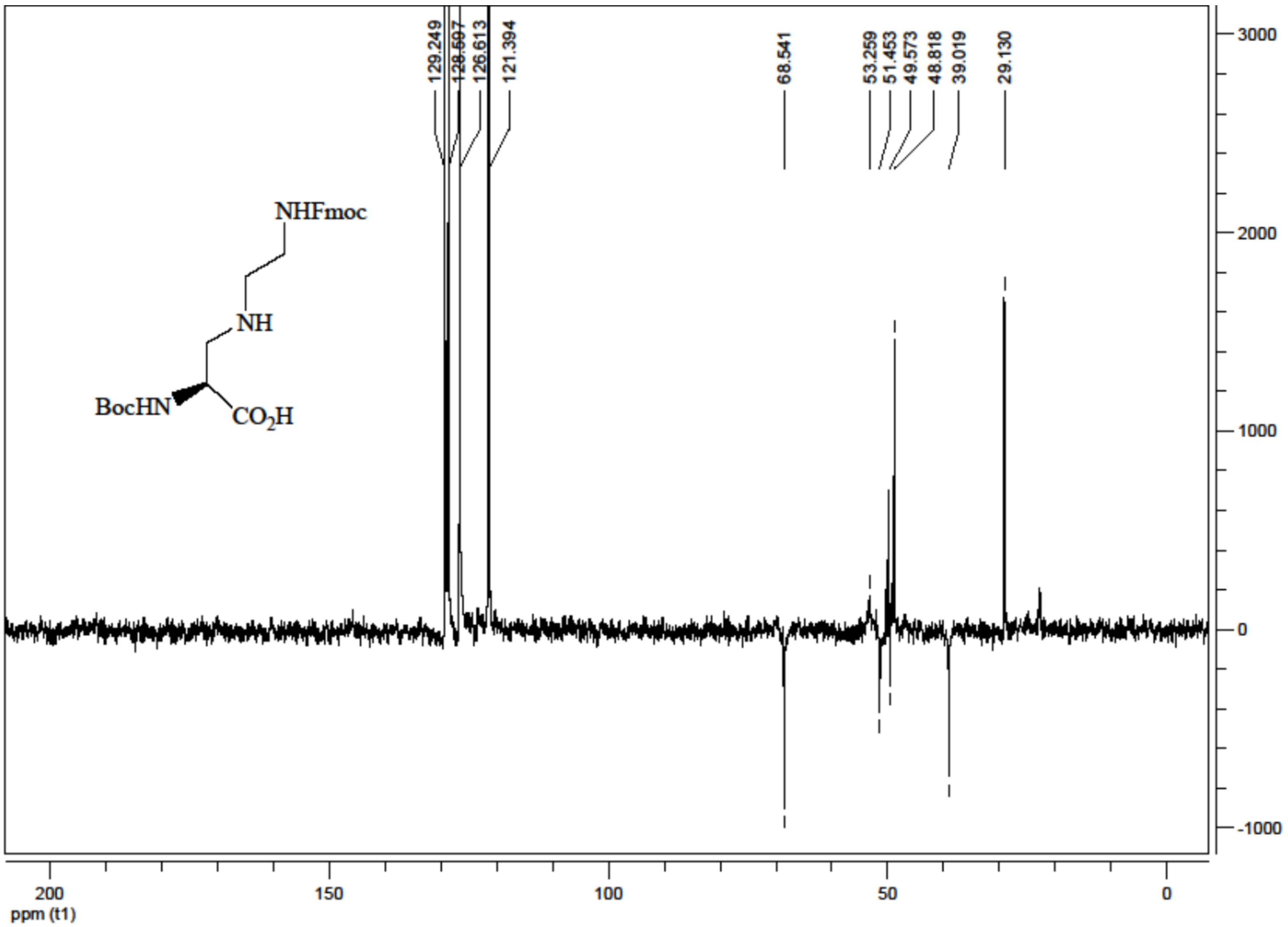

Figure S38. DEPT-135 spectrum of **22** (CD_3OD, 100 MHz).

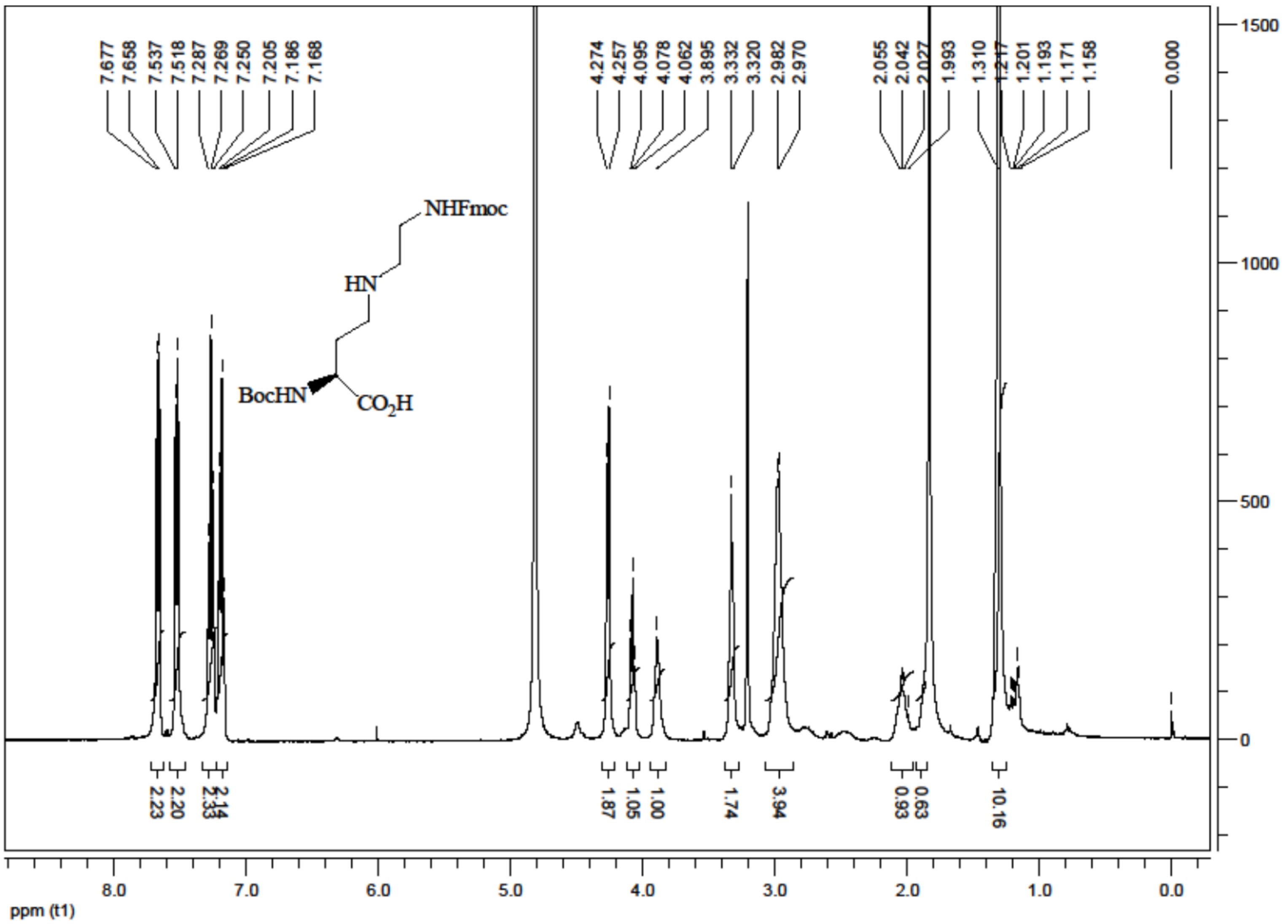

Figure S39. ^{1}H NMR spectrum of **23** (CD_3OD, 400 MHz).

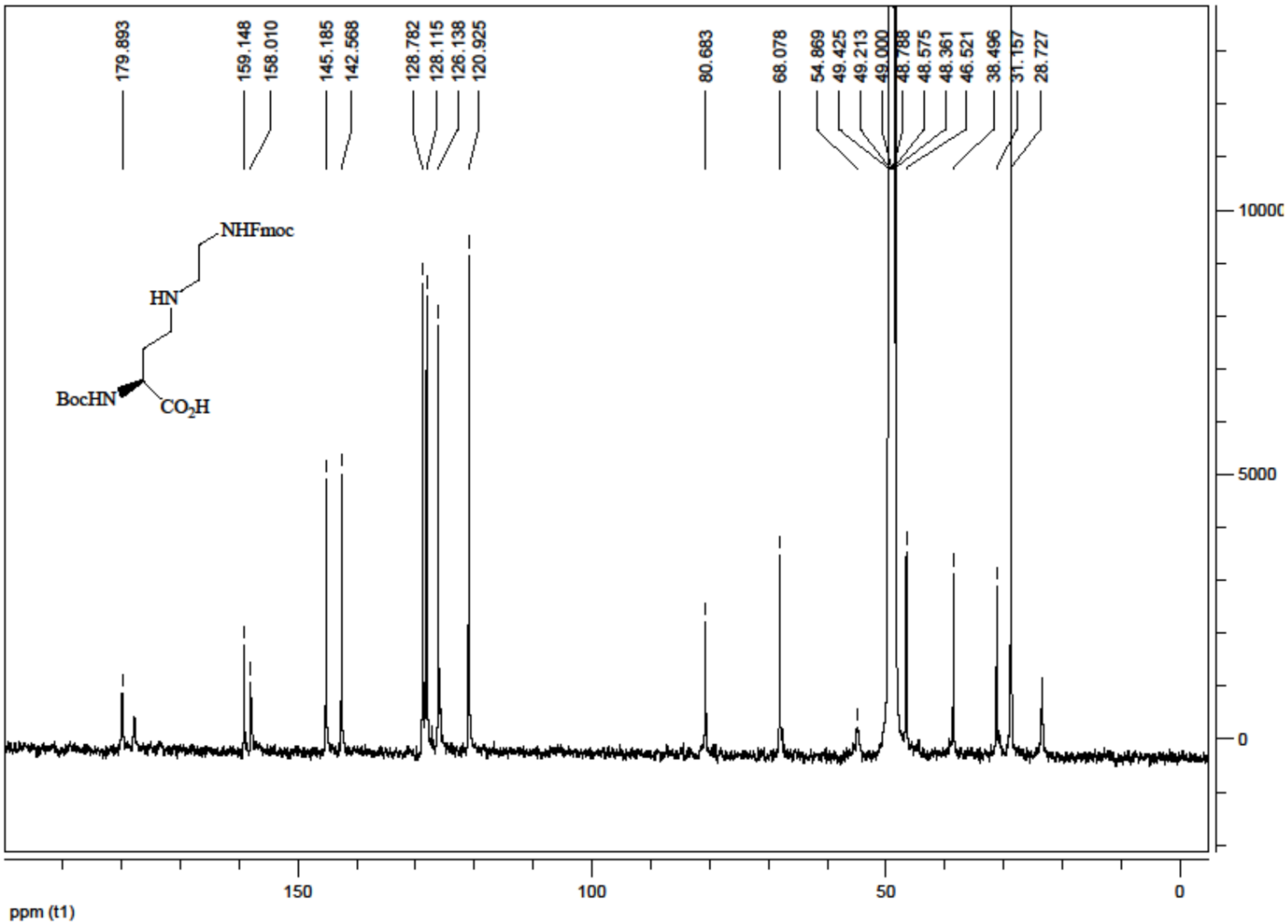

Figure S40. ^{13}C NMR spectrum of **23** (CD,OD, 100 MHz).

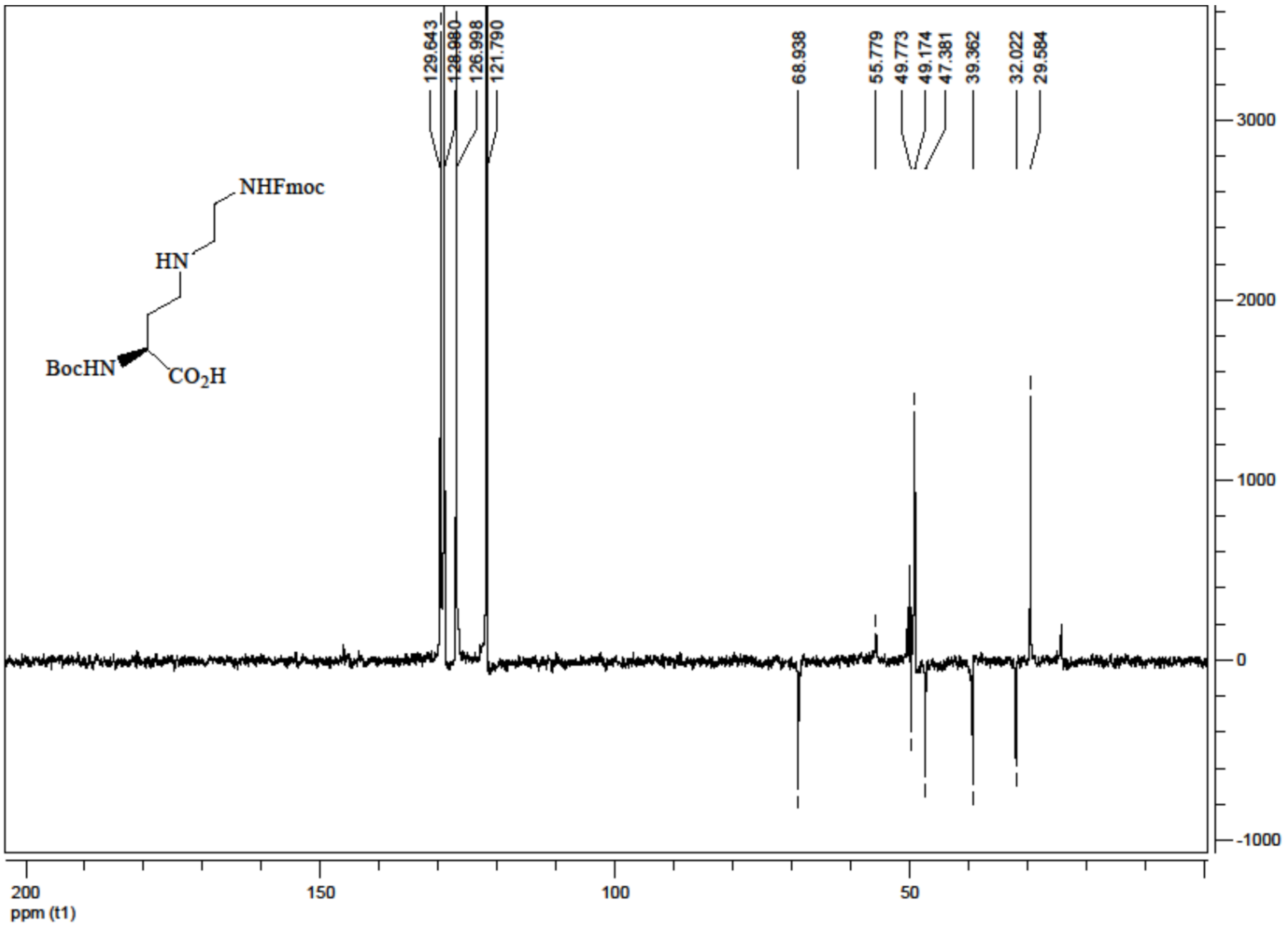

Figure S41. DEPT-135 spectrum of **23** (CD_3OD, 100 MHz).

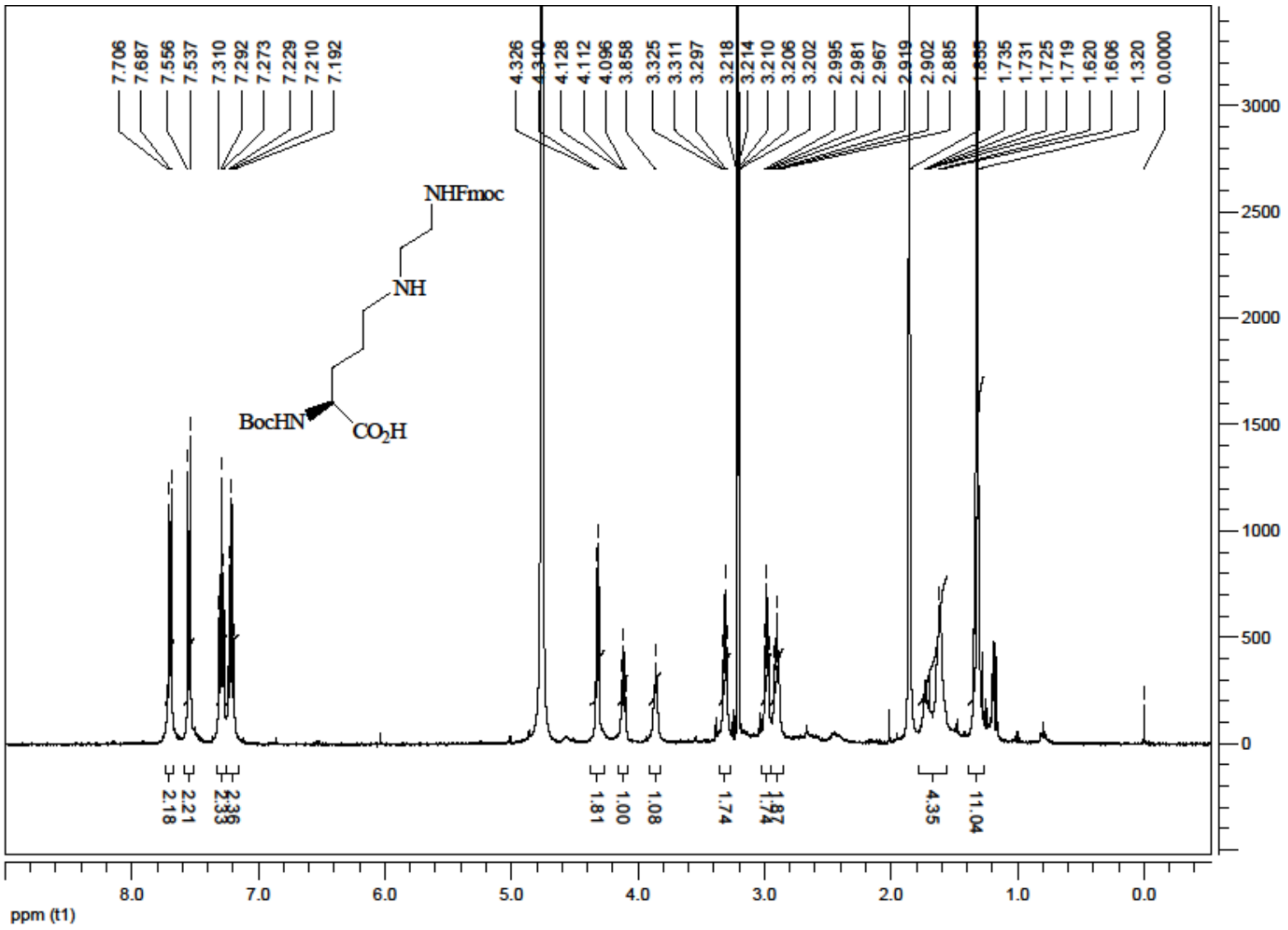

Figure S42. ^{1}H NMR spectrum of **24** (CD_3OD, 400 MHz).

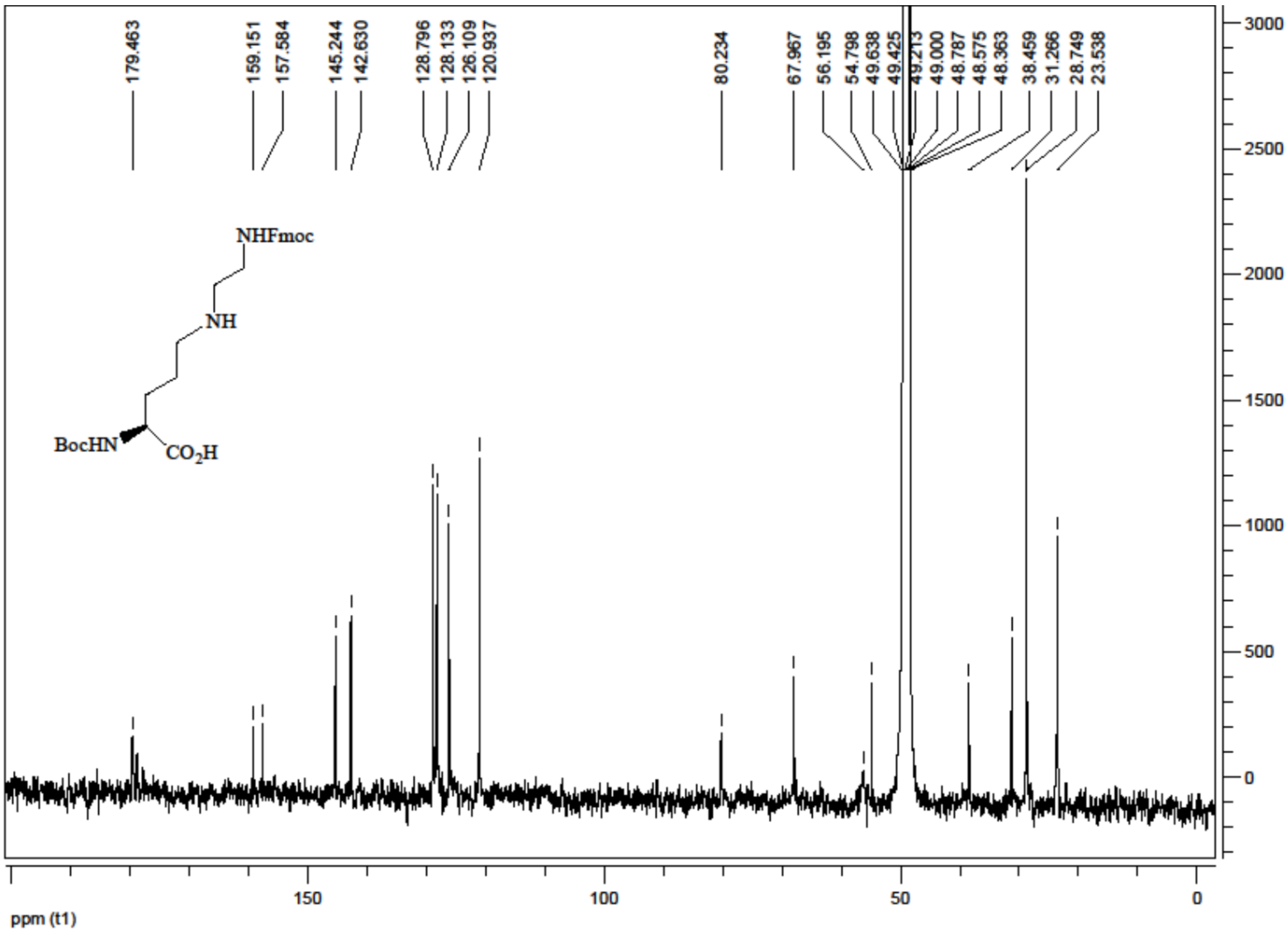

Figure S43. ^{13}C NMR spectrum of **24** (CD_3OD, 100 MHz).

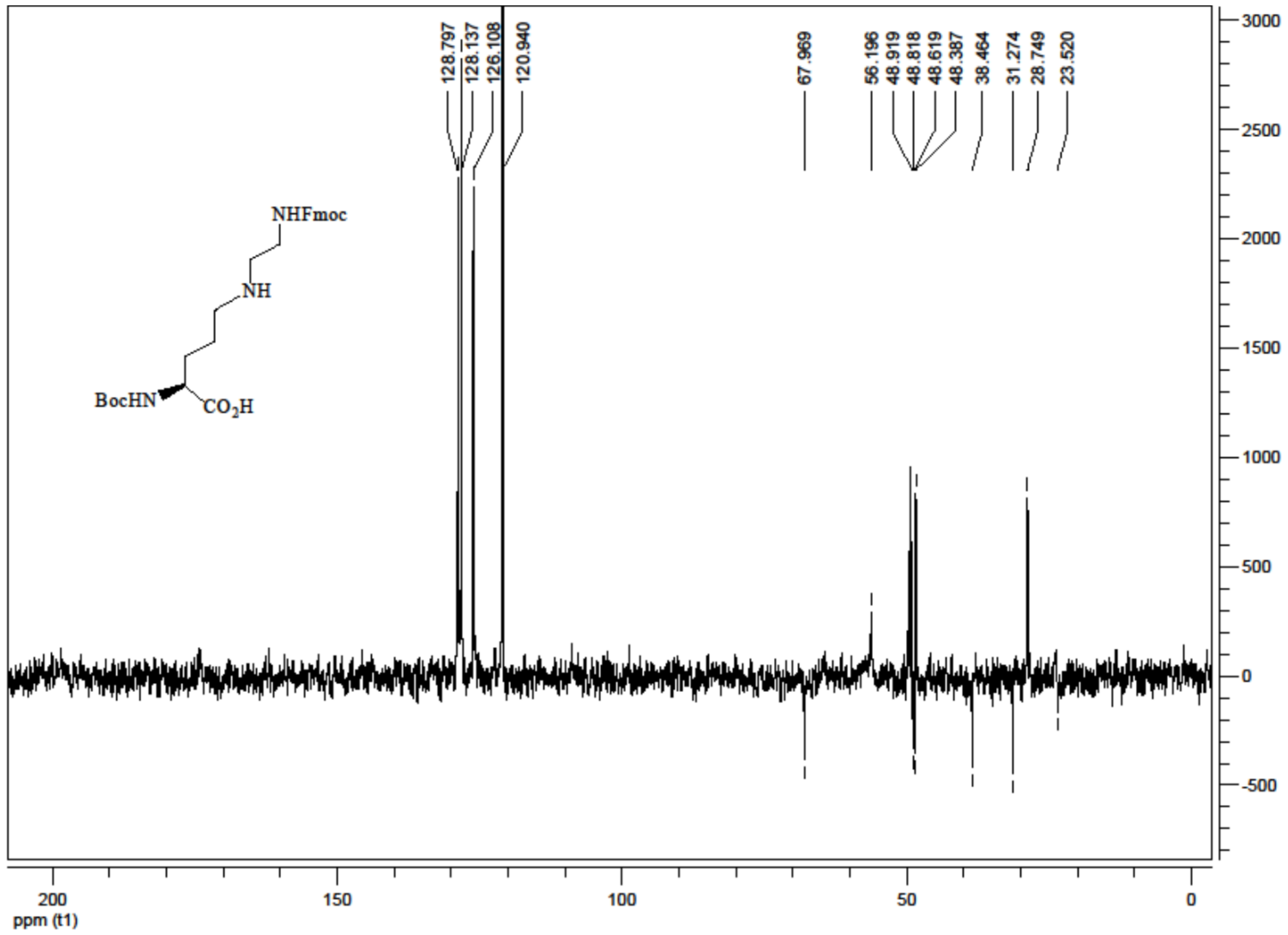

Figure S44. DEPT-135 spectrum of **24** (CD_3OD, 100 MHz).

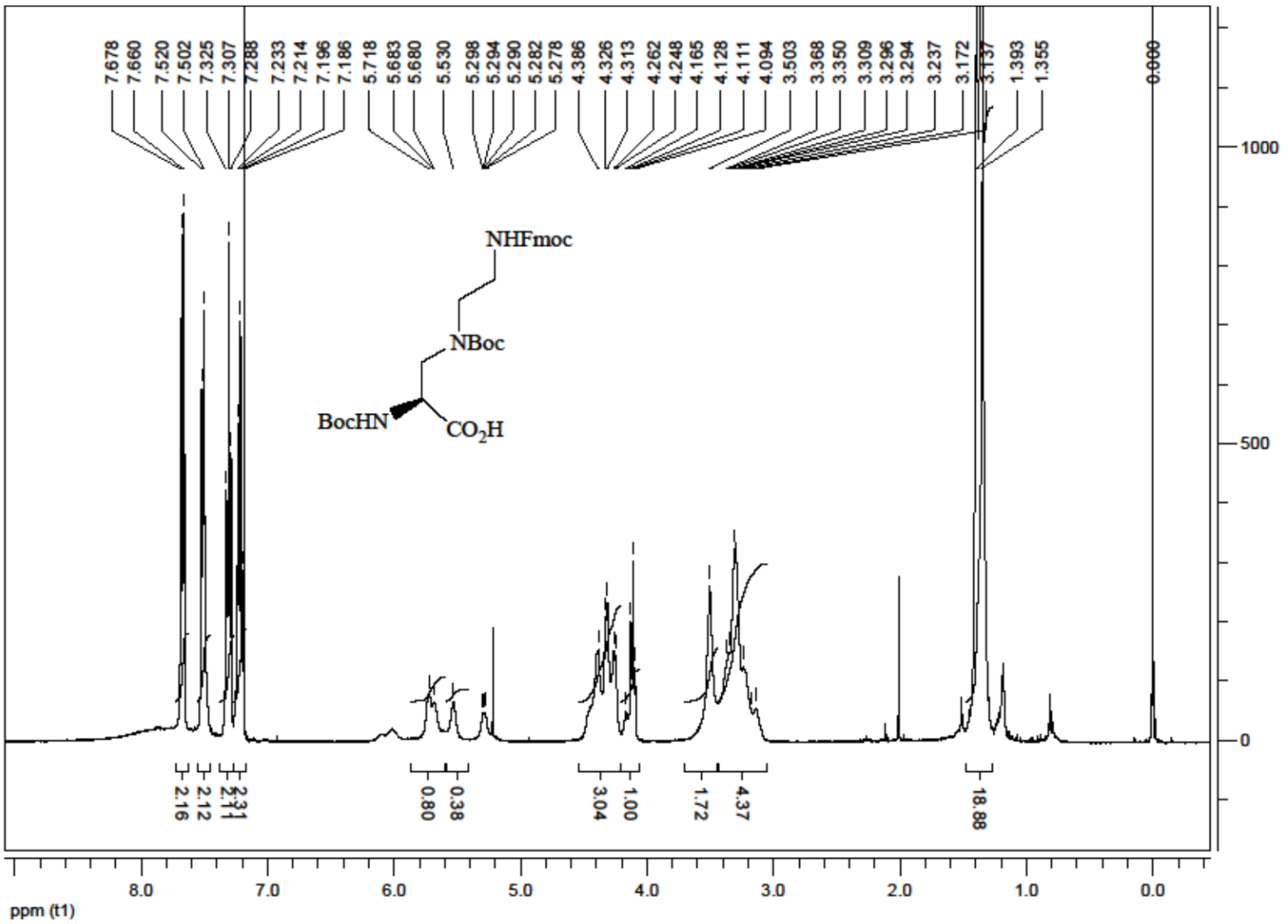

Figure S45. ^{1}H NMR spectrum of **25** ($CDCl_3$, 400 MHz).

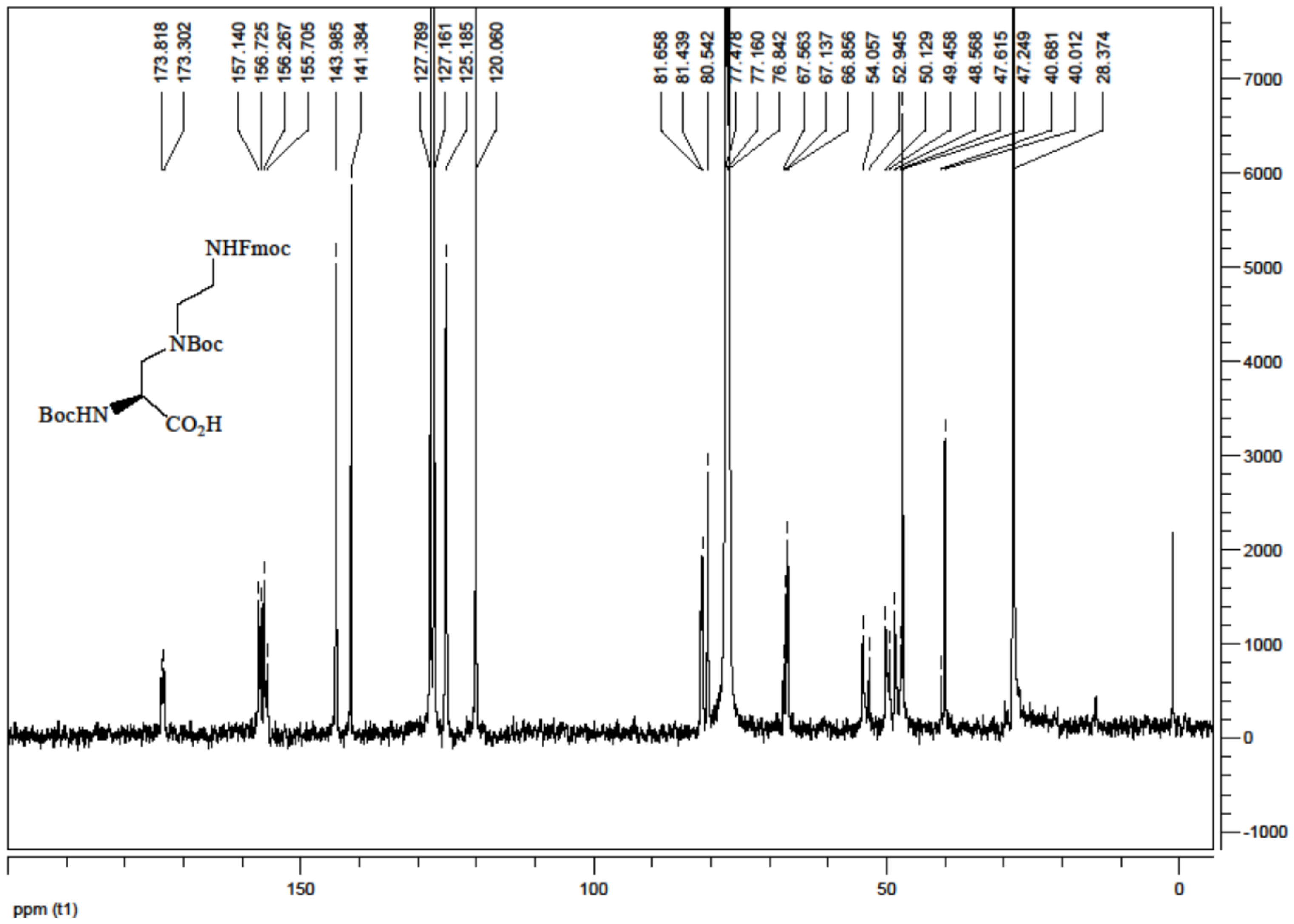

Figure S46. ^{1}H-^{1}H COSY spectrum of **25** in $CDCl_3$.

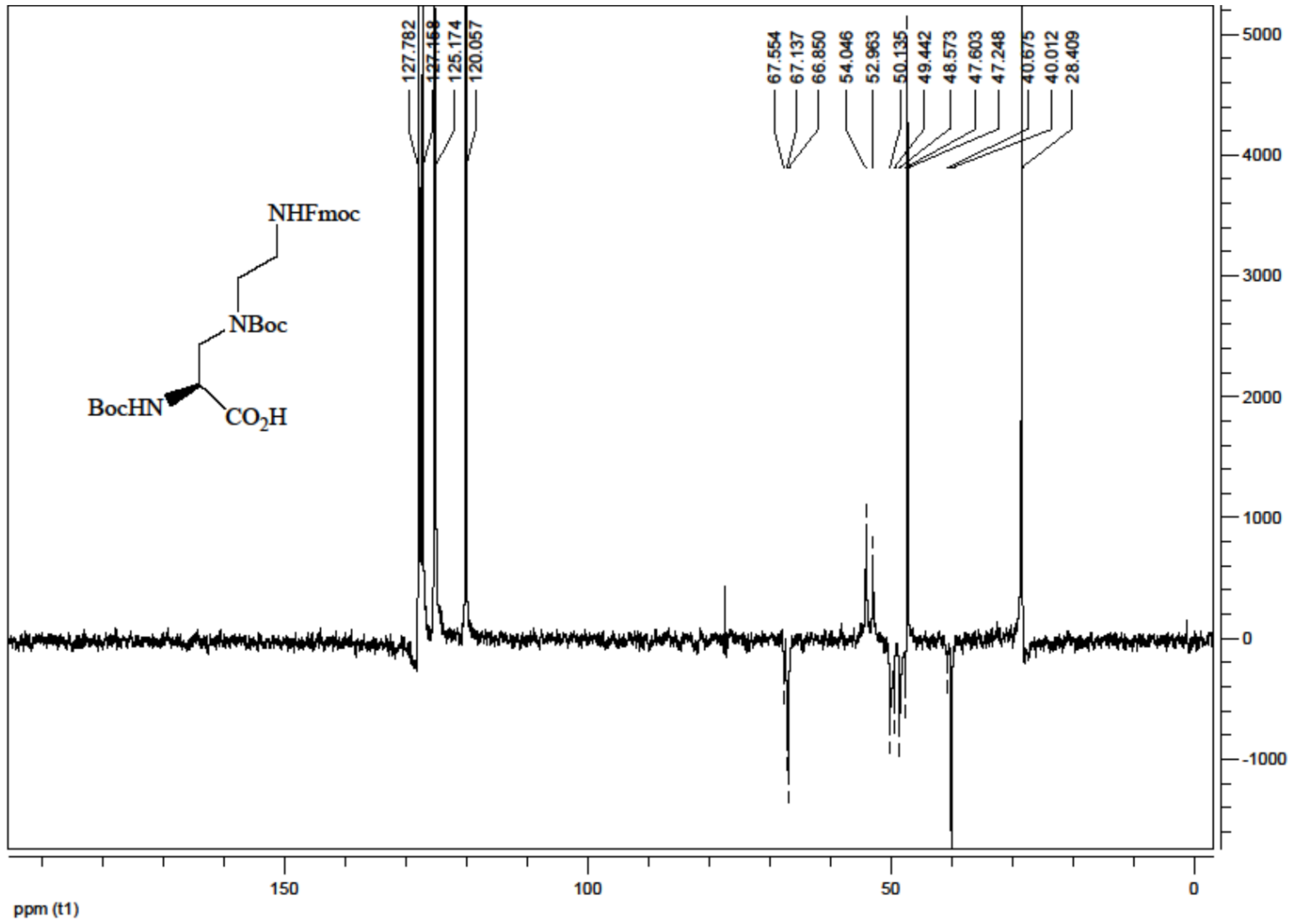

Figure S47. DEPT-135 spectrum of **25** ($CDCl_3$, 100 MHz).

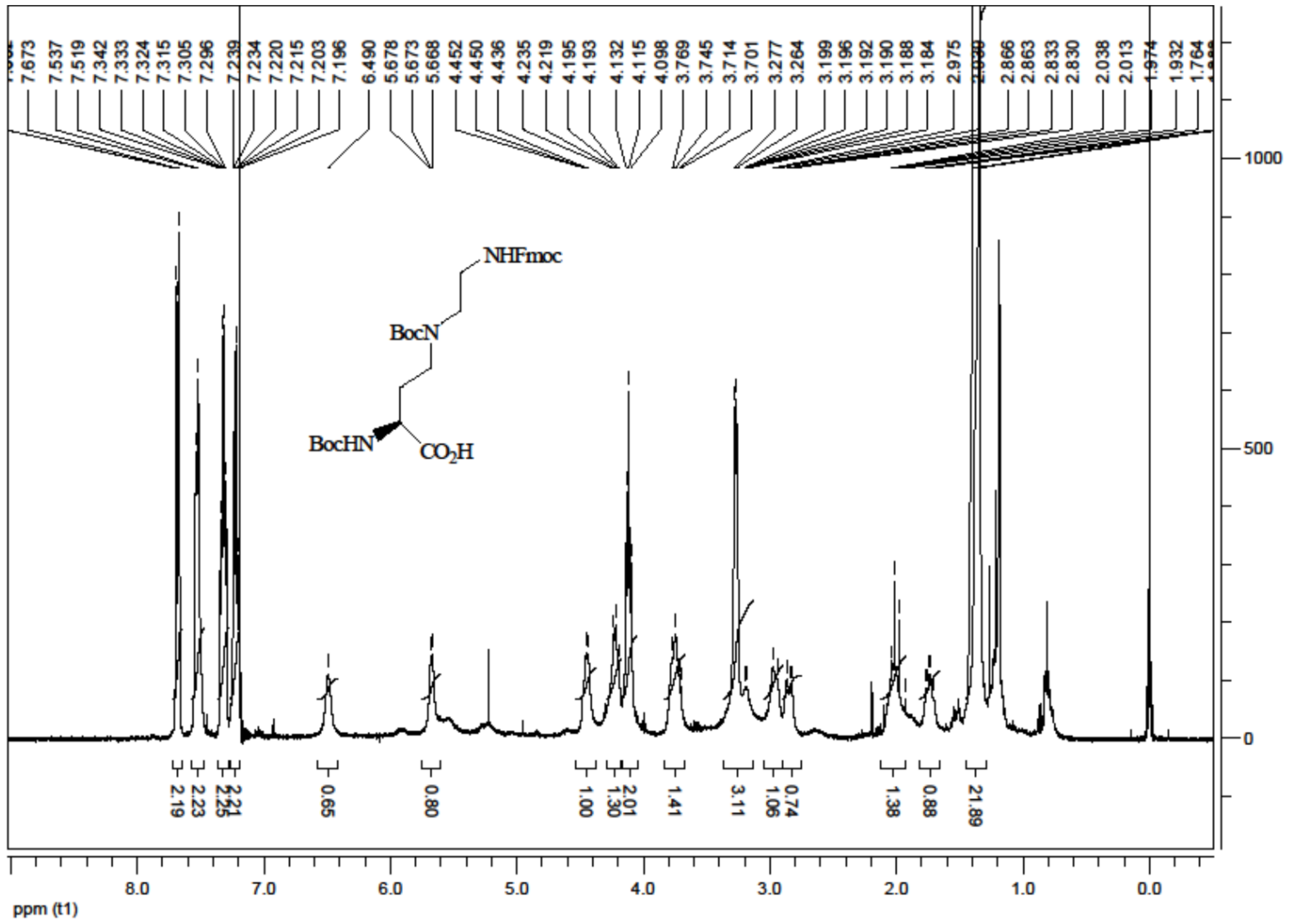

Figure S48. ^{1}H NMR spectrum of **26** ($CDCl_3$, 400 MHz).

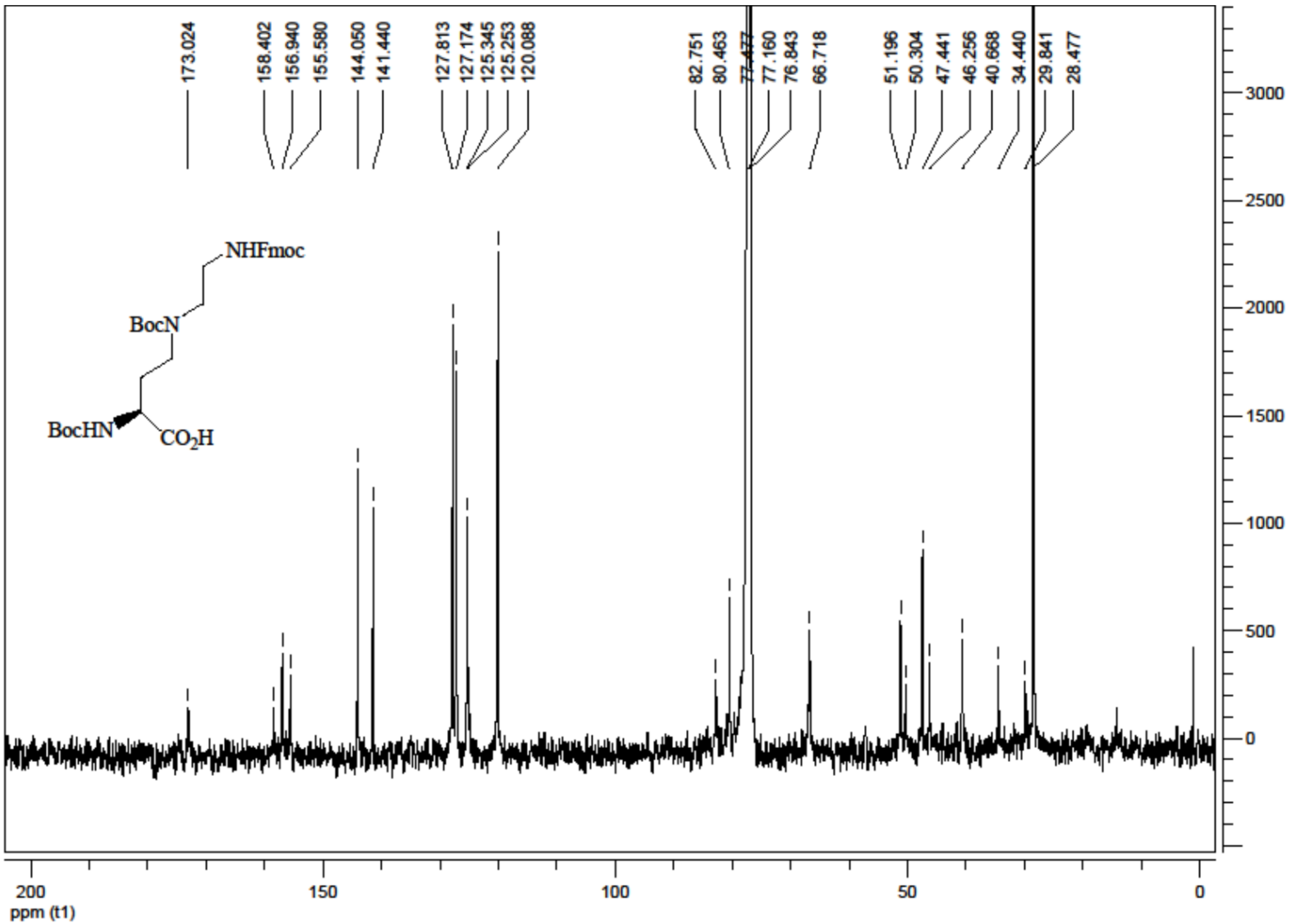

Figure S49. ^{13}C NMR spectrum of **26** ($CDCl_3$, 100 MHz).

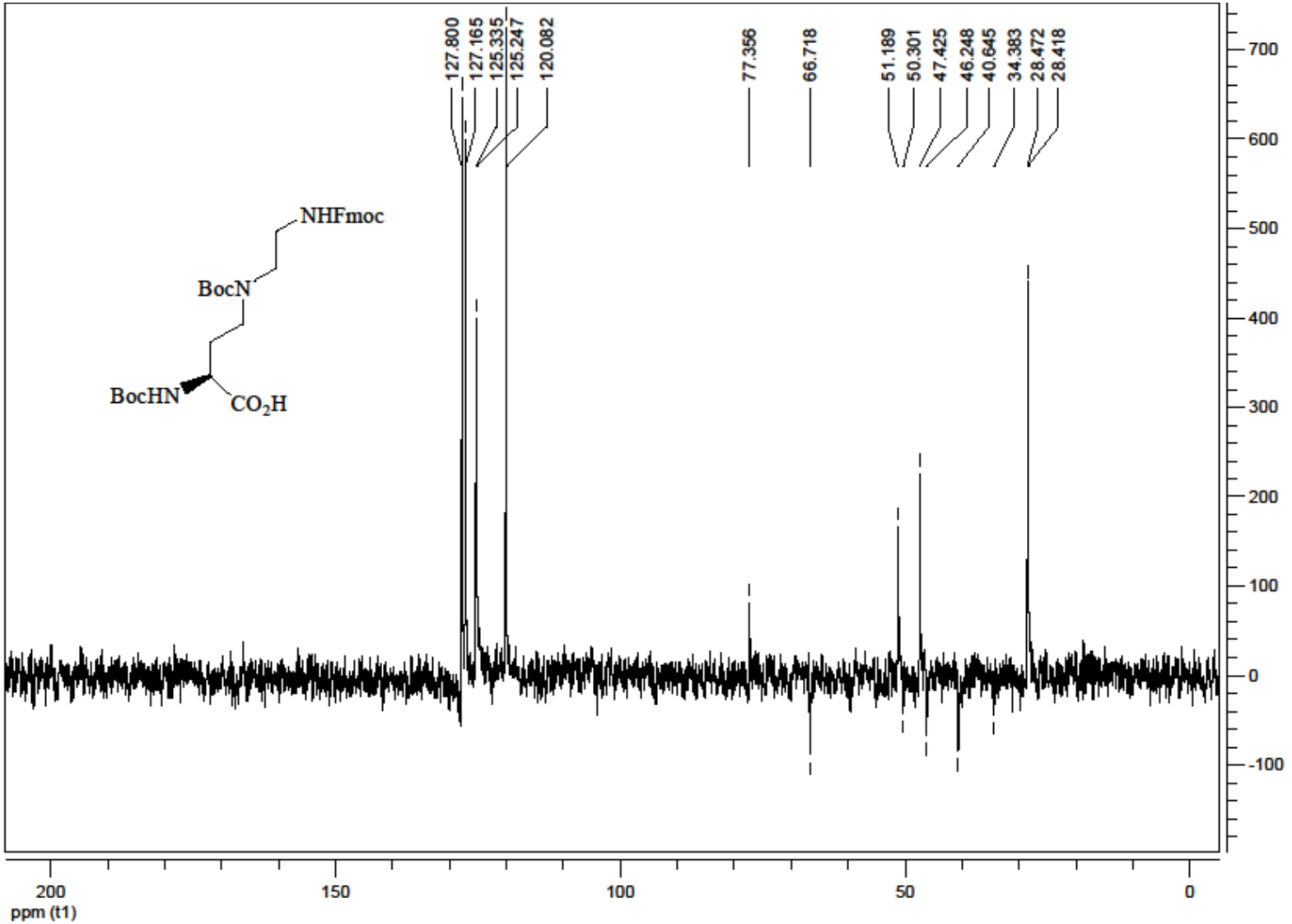

Figure S50. DEPT-135 spectrum of **26** ($CDCl_3$, 100 MHz).

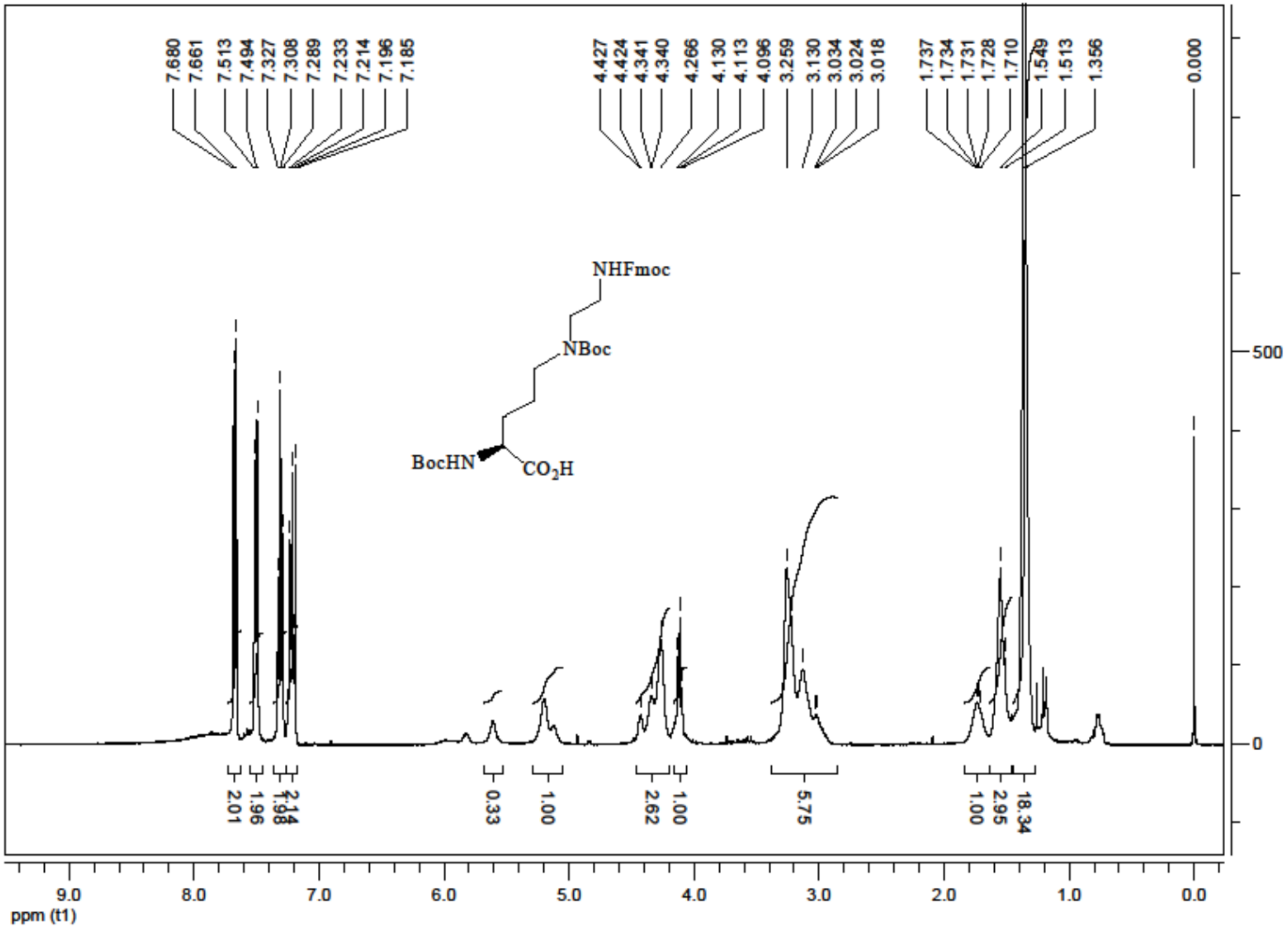

Figure S51. ^{1}H NMR spectrum of **27** ($CDCl_3$, 400 MHz).

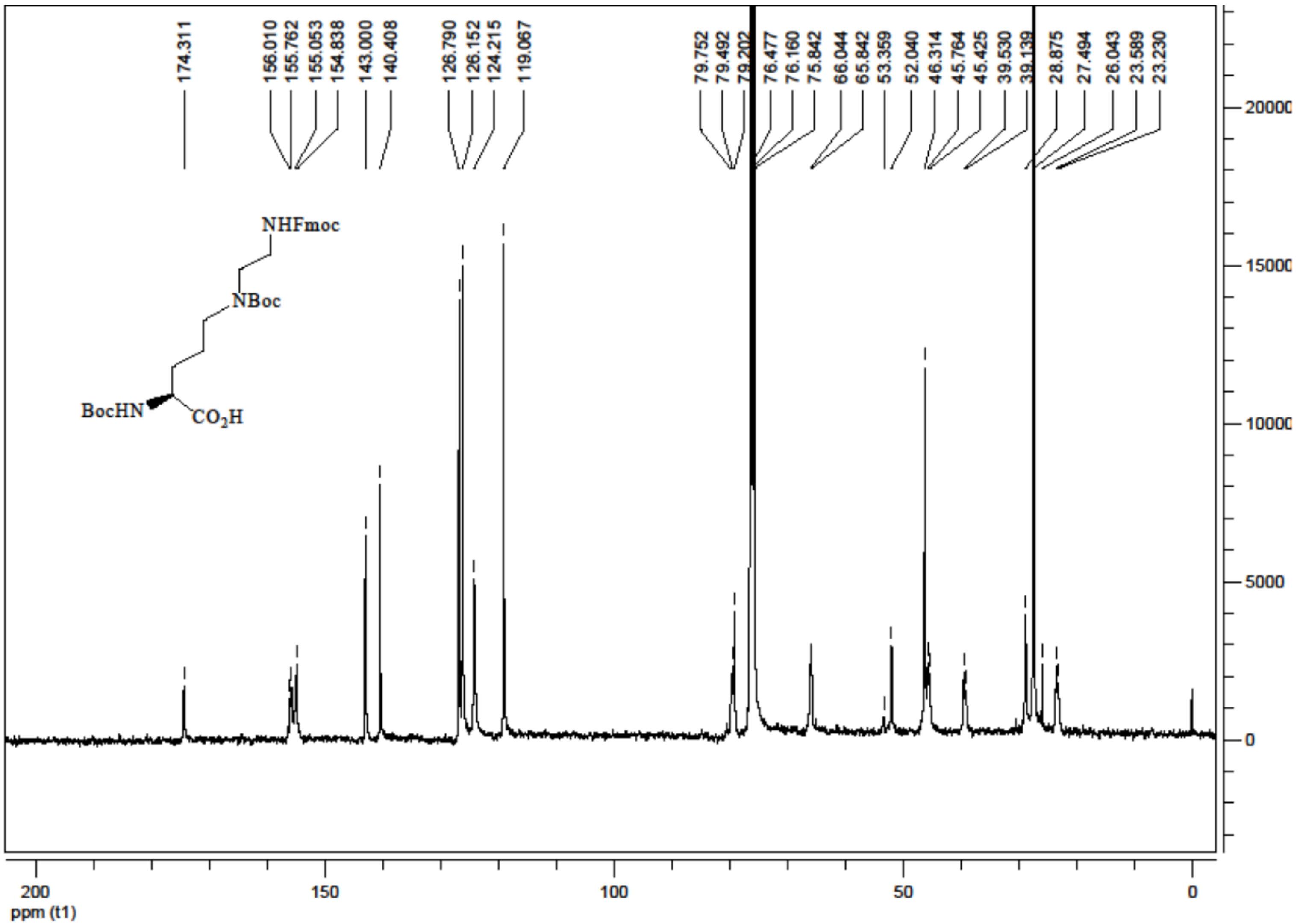

Figure S52. ^{13}C NMR spectrum of **27** ($CDCl_3$, 100 MHz).

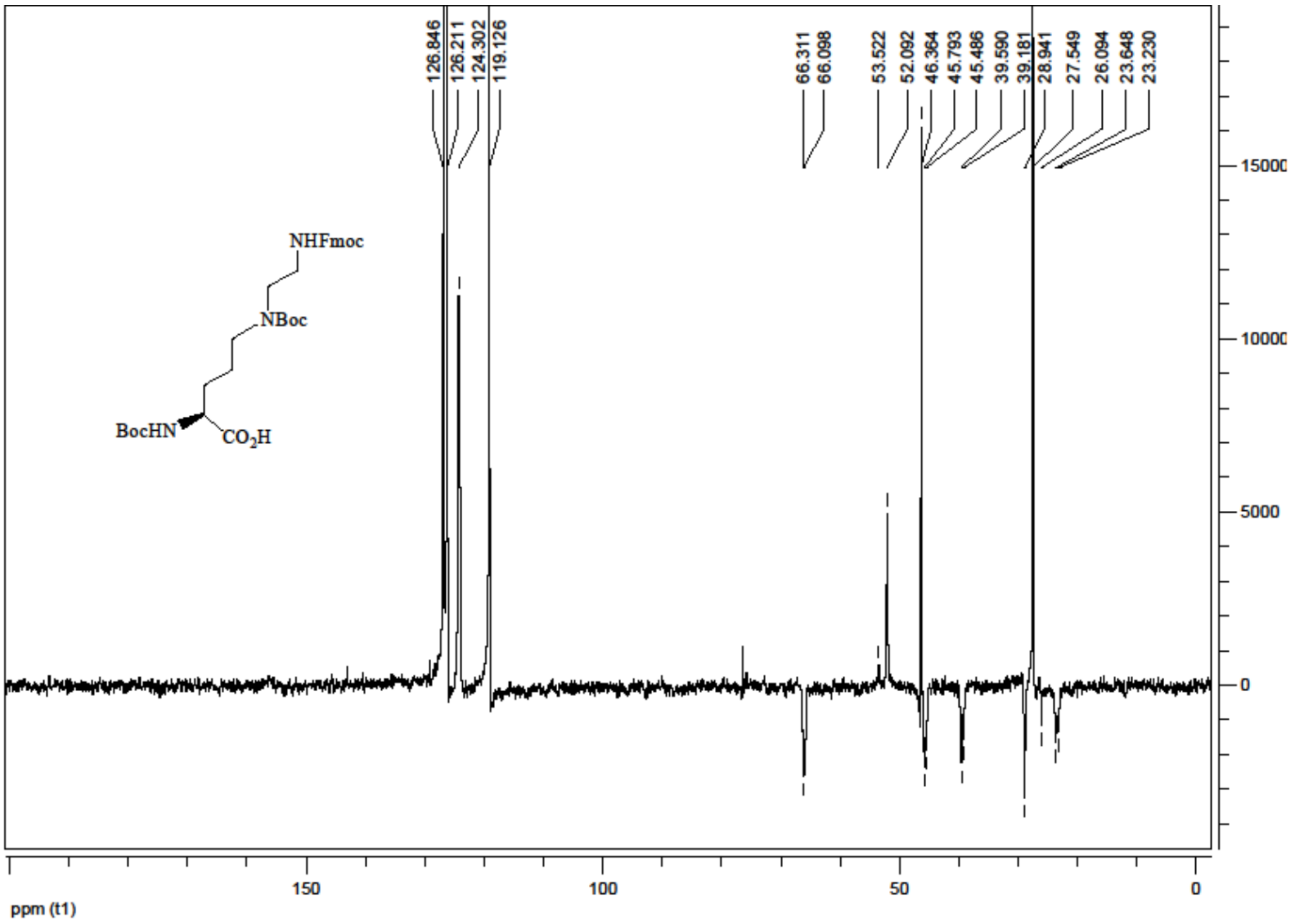

Figure S53. DEPT-135 spectrum of **27** ($CDCl_3$, 100 MHz).

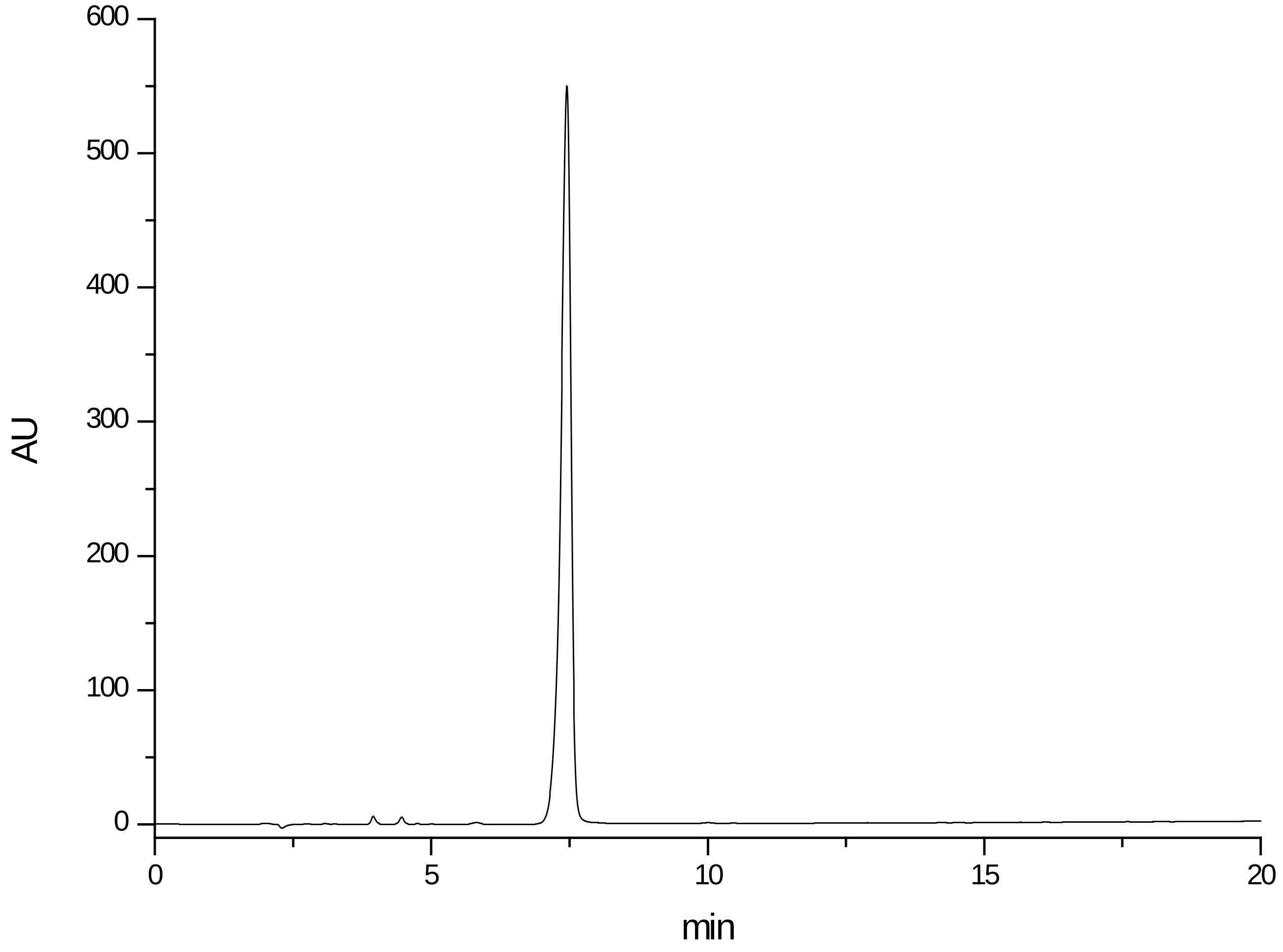

Figure S54. RP-HPLC chromatogram of compound **14**.

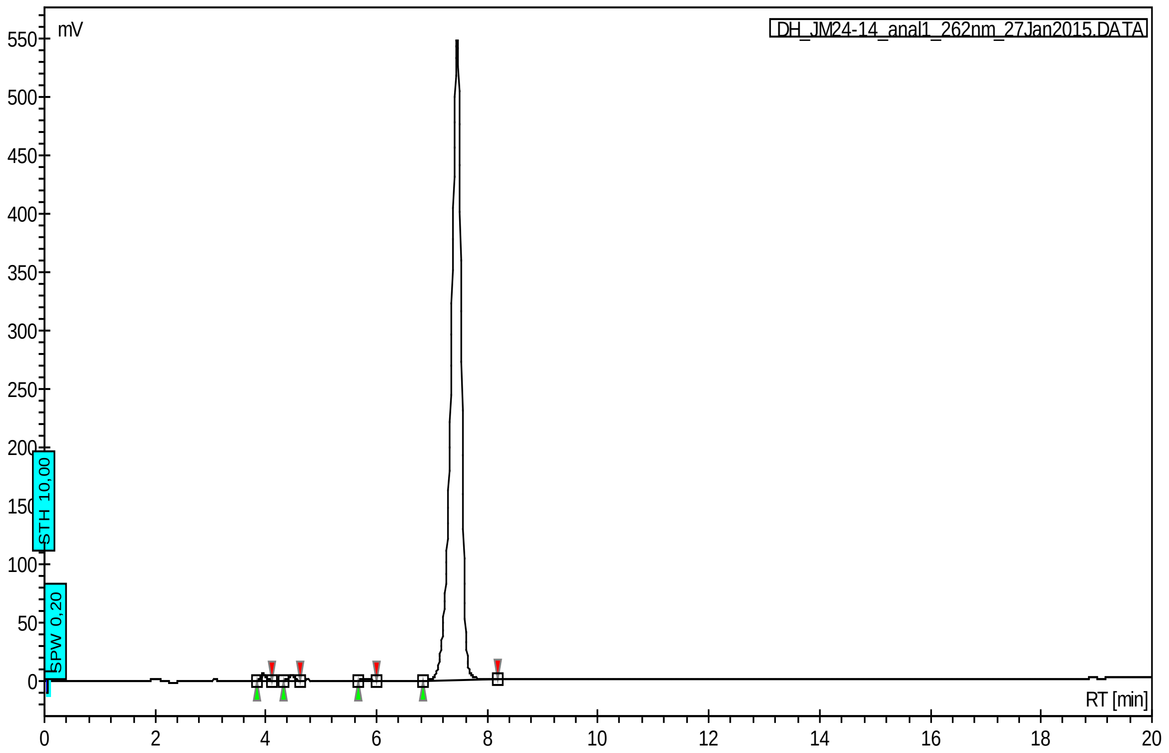

#	Time [Min]	Quantity [% Area]	Height [mV]	Area [mV.Min]	Area % [%]
1	3,950	0,48	6,1	0,6	0,479
2	4,458	0,47	5,4	0,6	0,469
3	5,817	0,21	1,4	0,2	0,205
4	7,450	98,85	549,5	117,9	98,847
Total		100,00	562,3	119,3	100,000

Figure S55. Integration of RP-HPLC chromatogram of compound **14**.

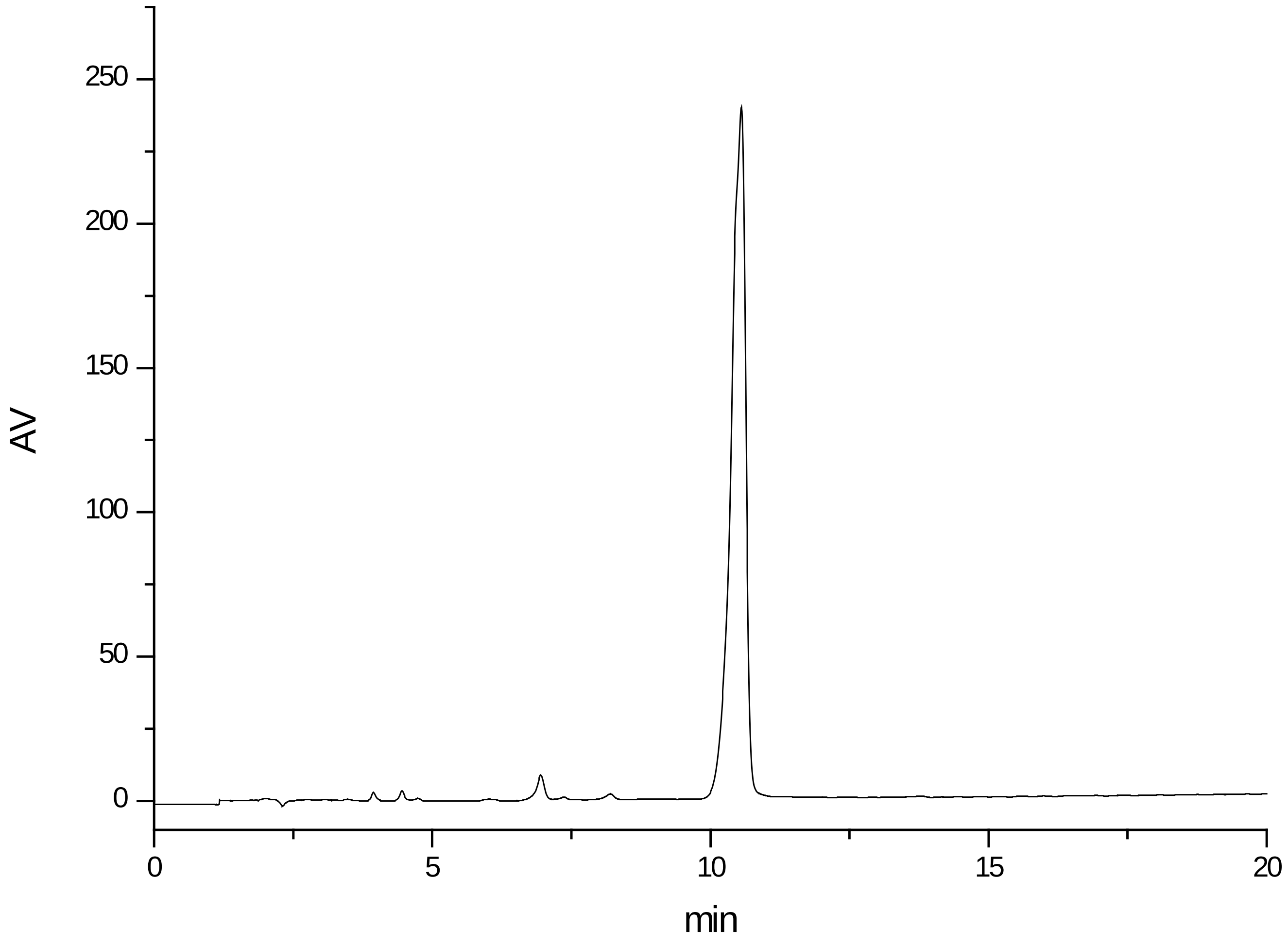

Figure S56. RP-HPLC chromatogram of compound **15**.

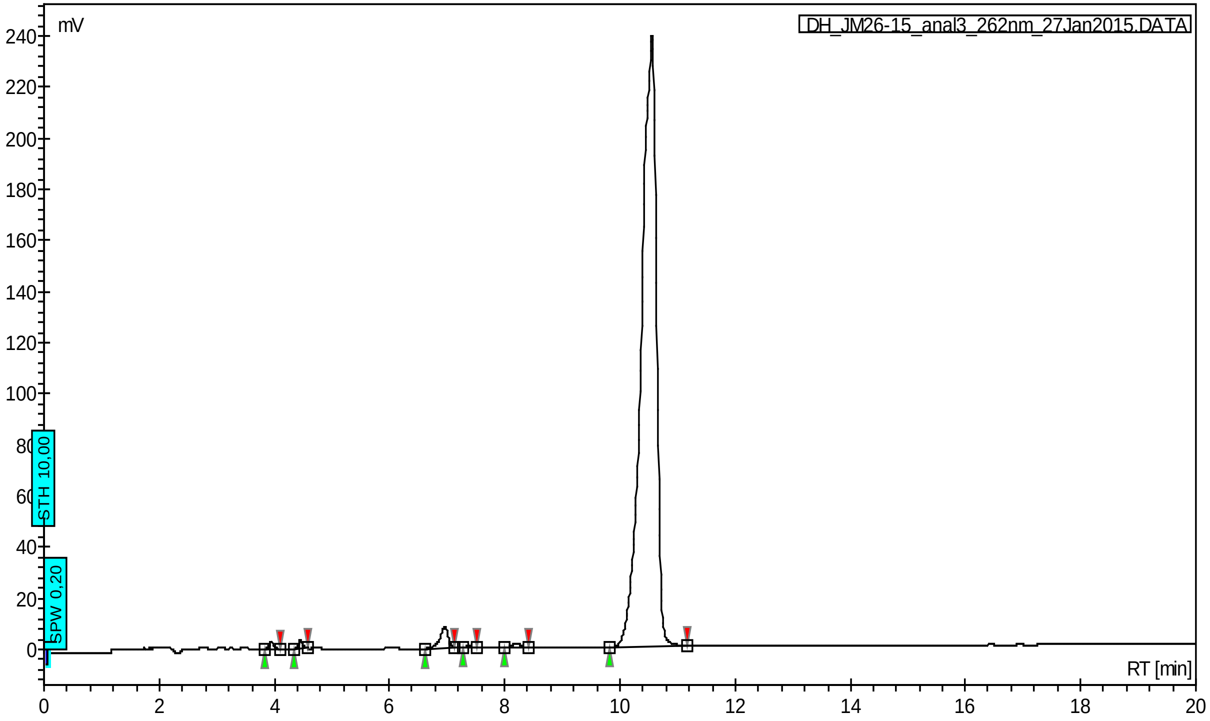

#	Time [Min]	Quantity [% Area]	Height [mV]	Area [mV.Min]	Area % [%]
1	3,942	0,37	2,9	0,3	0,366
2	4,450	0,41	3,3	0,3	0,413
3	6,950	1,87	8,5	1,4	1,870
4	7,375	0,10	0,7	0,1	0,099
5	8,200	0,40	1,8	0,3	0,400
6	10,558	96,85	239,2	72,7	96,852
Total		100,00	256,4	75,1	100,000

Figure S57. Integration of RP-HPLC chromatogram of compound **15**.

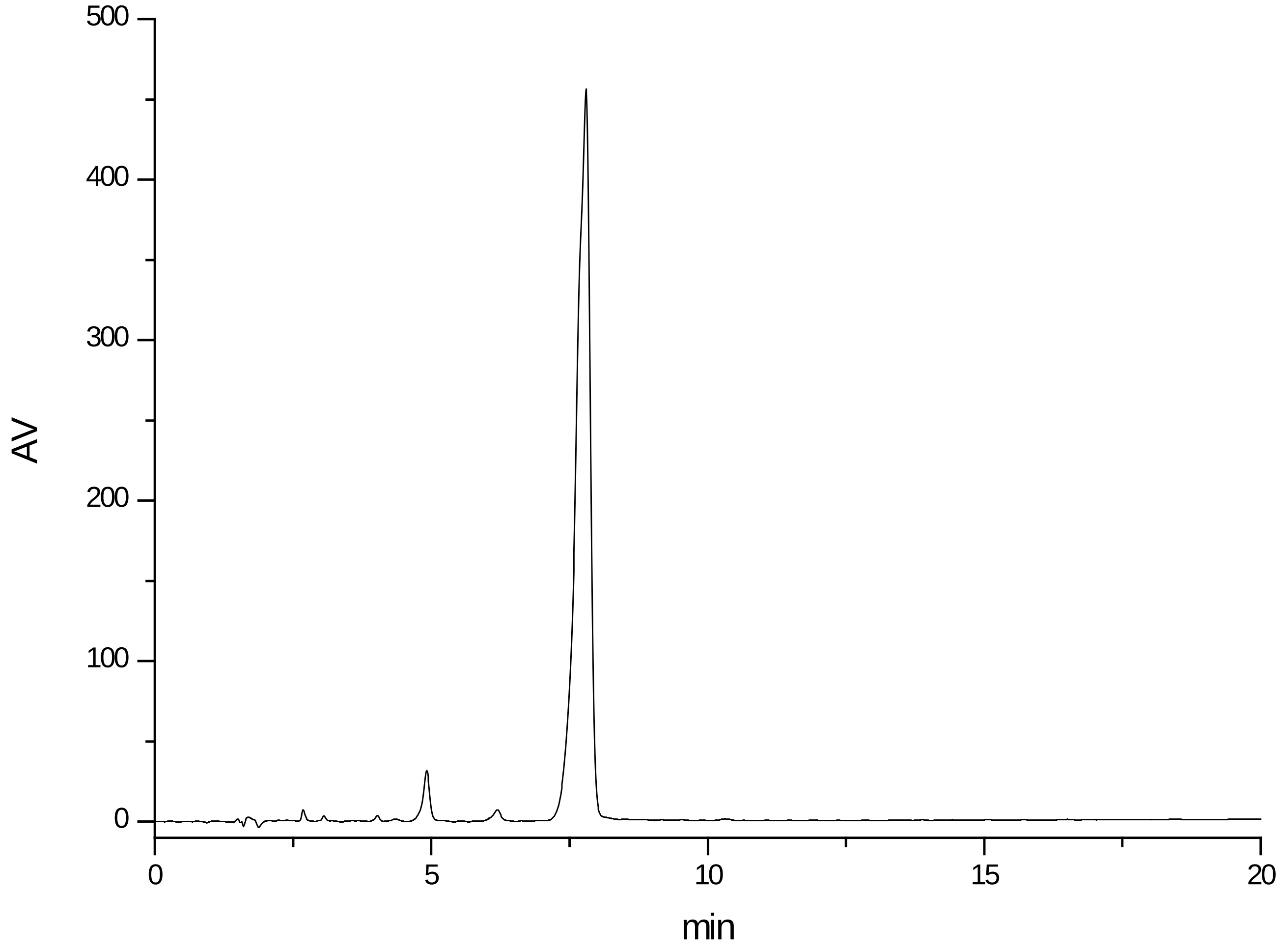

Figure S58. RP-HPLC chromatogram of compound **16**.

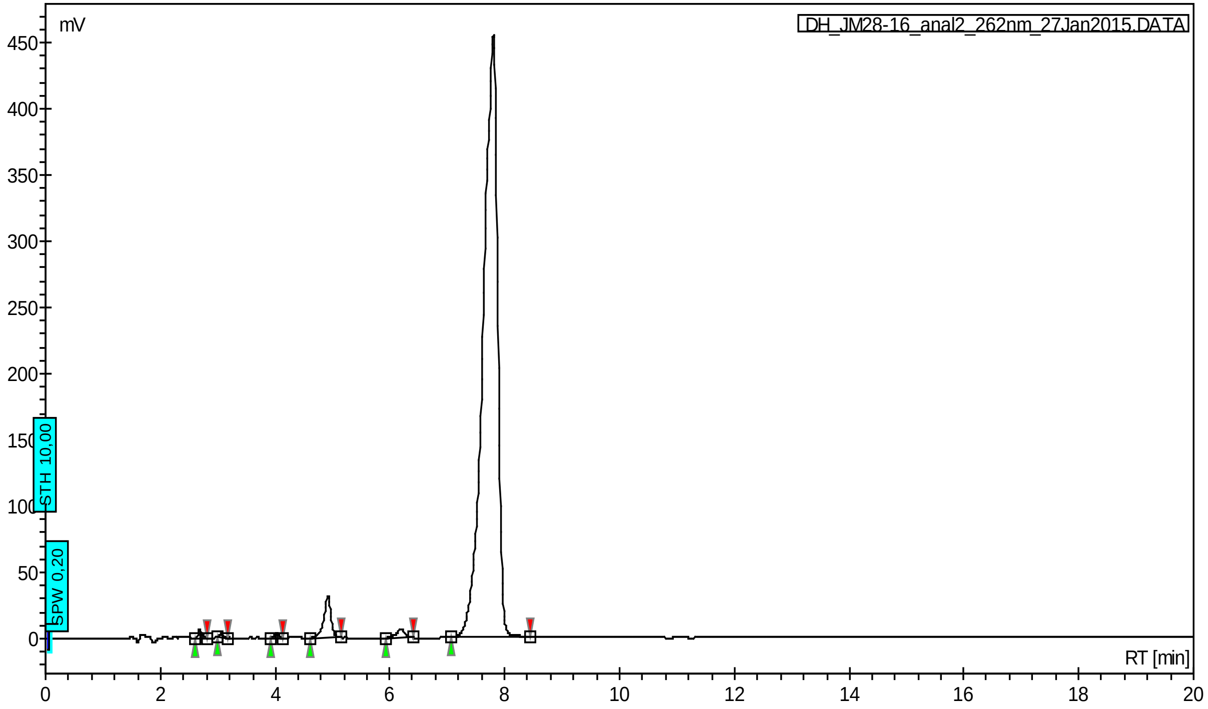

#	Time [Min]	Quantity [% Area]	Height [mV]	Area [mV.Min]	Area % [%]
1	2,683	0,34	6,9	0,5	0,344
2	3,058	0,14	2,9	0,2	0,138
3	4,025	0,23	3,4	0,3	0,232
4	4,917	3,24	31,3	4,5	3,245
5	6,192	0,82	6,9	1,1	0,819
6	7,800	95,22	455,6	132,7	95,222
Total		100,00	507,1	139,3	100,000

Figure S59. Integration of RP-HPLC chromatogram of compound **16**.

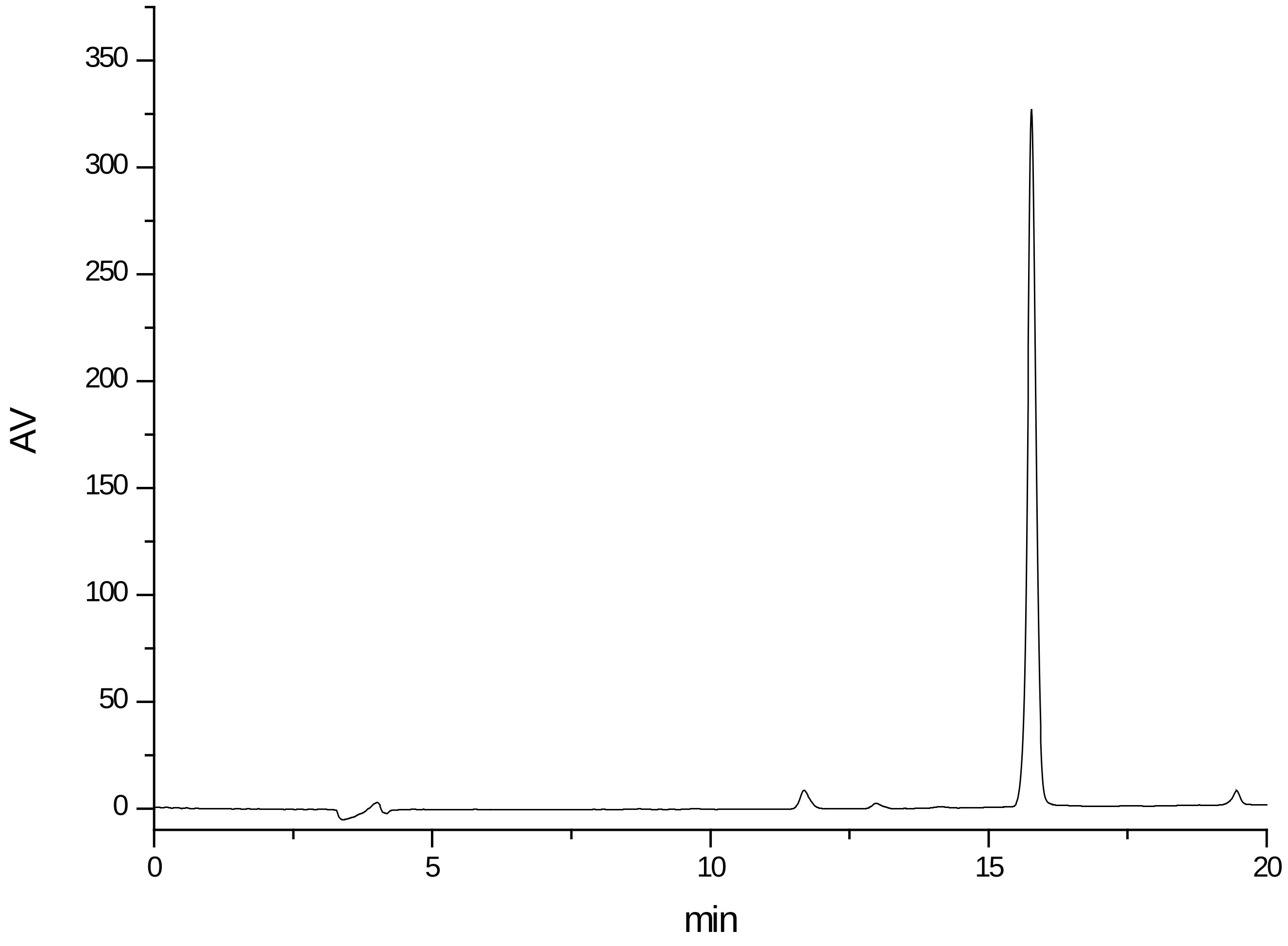

Figure S60. RP-HPLC chromatogram of compound **25**.

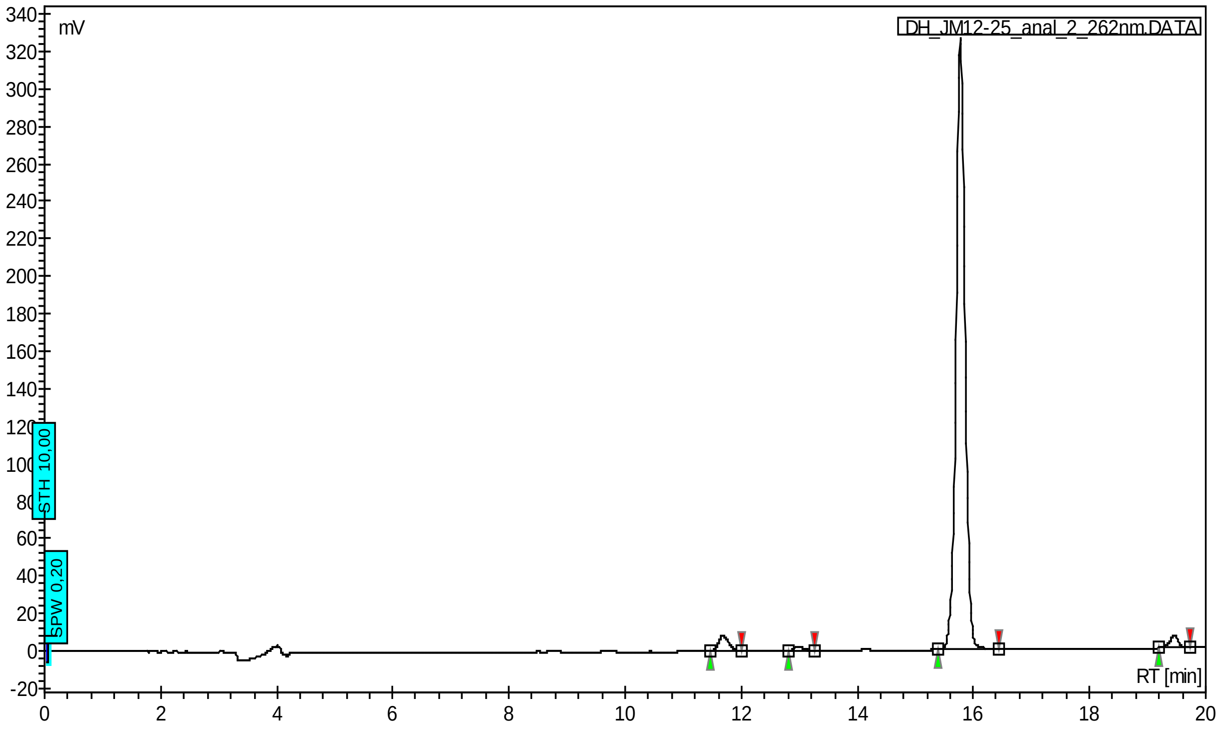

#	Time [Min]	Quantity [% Area]	Height [mV]	Area [mV.Min]	Area % [%]
1	11,683	2,84	8,5	1,7	2,840
2	12,983	0,78	2,4	0,5	0,783
3	15,775	94,60	326,4	58,2	94,598
4	19,458	1,78	6,6	1,1	1,779
Total		100,00	343,9	61,5	100,000

Figure S61. Integration of RP-HPLC chromatogram of compound **25**.

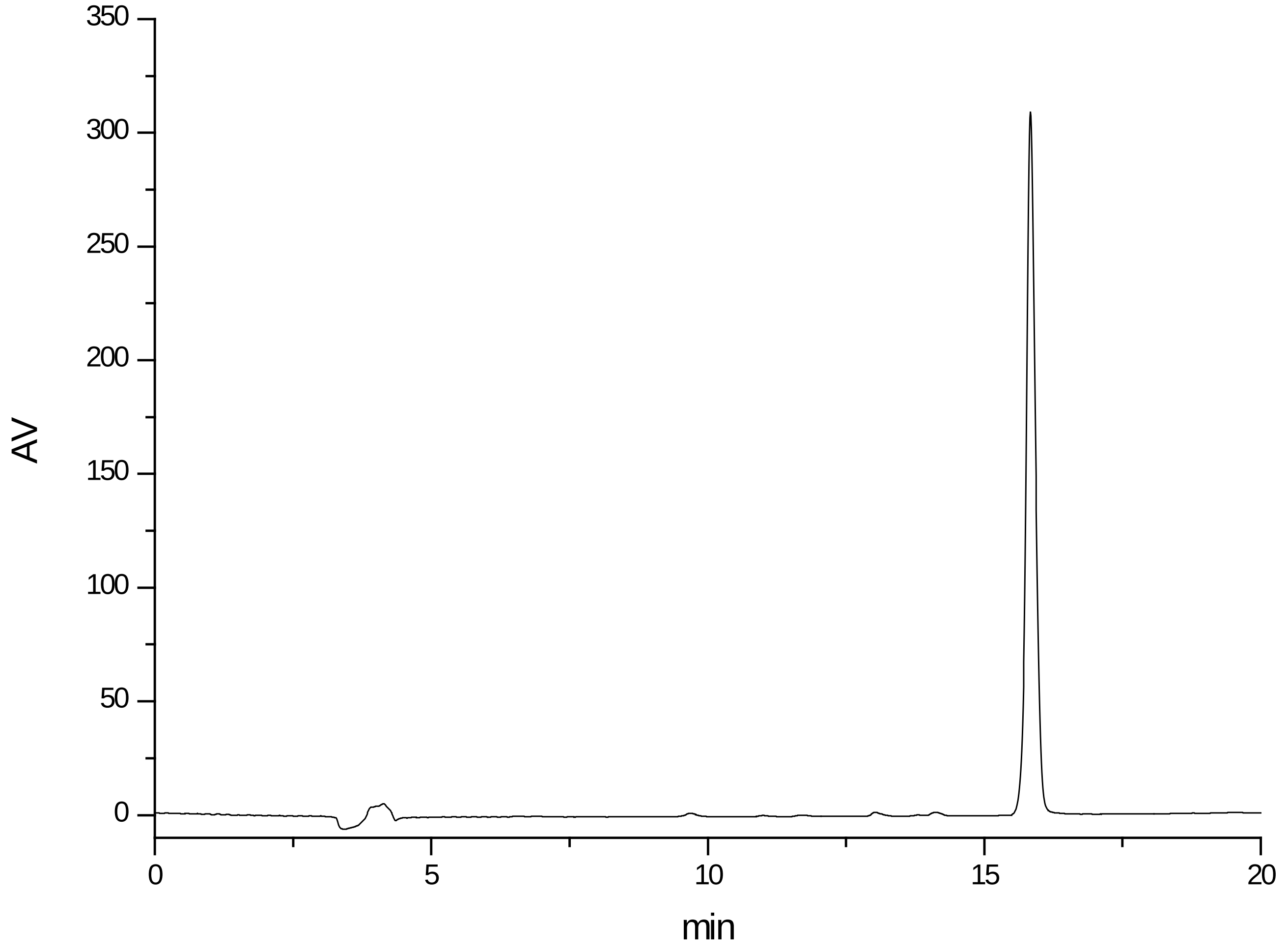

Figure S62. RP-HPLC chromatogram of compound **26**.

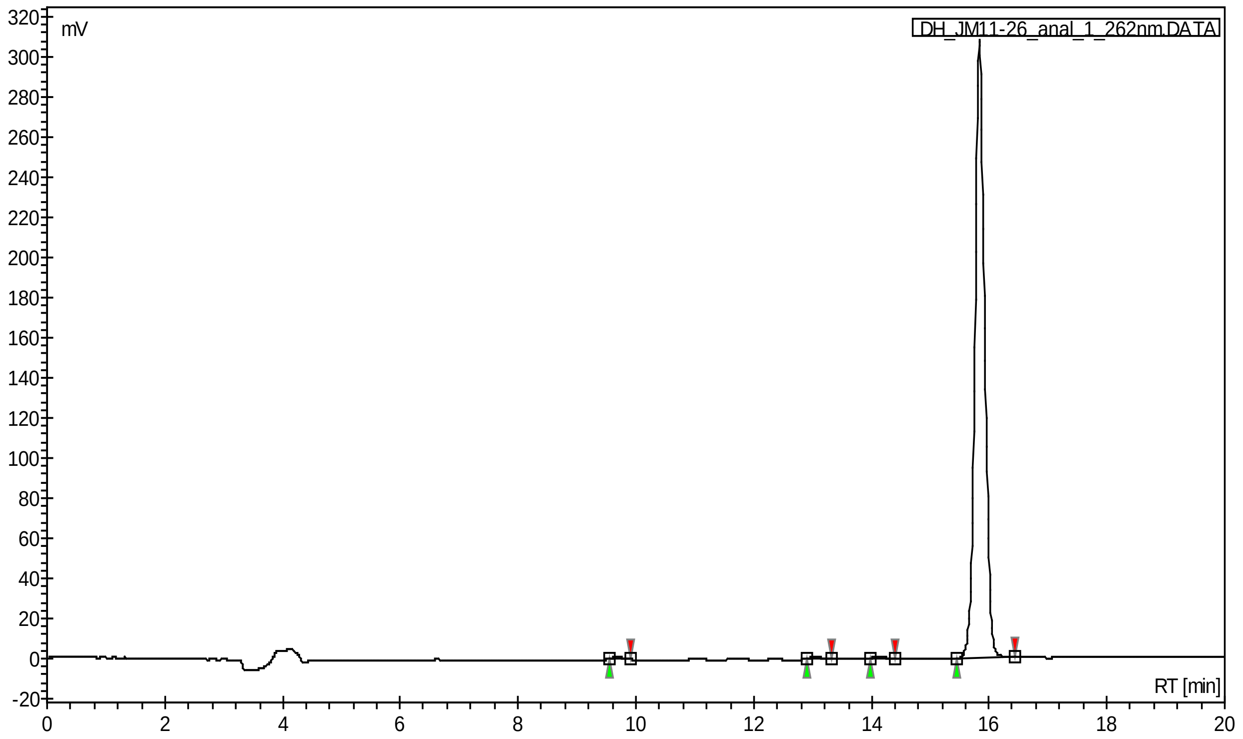

#	Time [Min]	Quantity [% Area]	Height [mV]	Area [mV.Min]	Area % [%]
1	9,683	0,36	1,1	0,2	0,359
2	13,025	0,54	1,6	0,3	0,545
3	14,117	0,48	1,4	0,3	0,484
4	15,833	98,61	309,2	59,0	98,612
Total		100,00	313,4	59,9	100,000

Figure S63. Integration of RP-HPLC chromatogram of compound **26**.

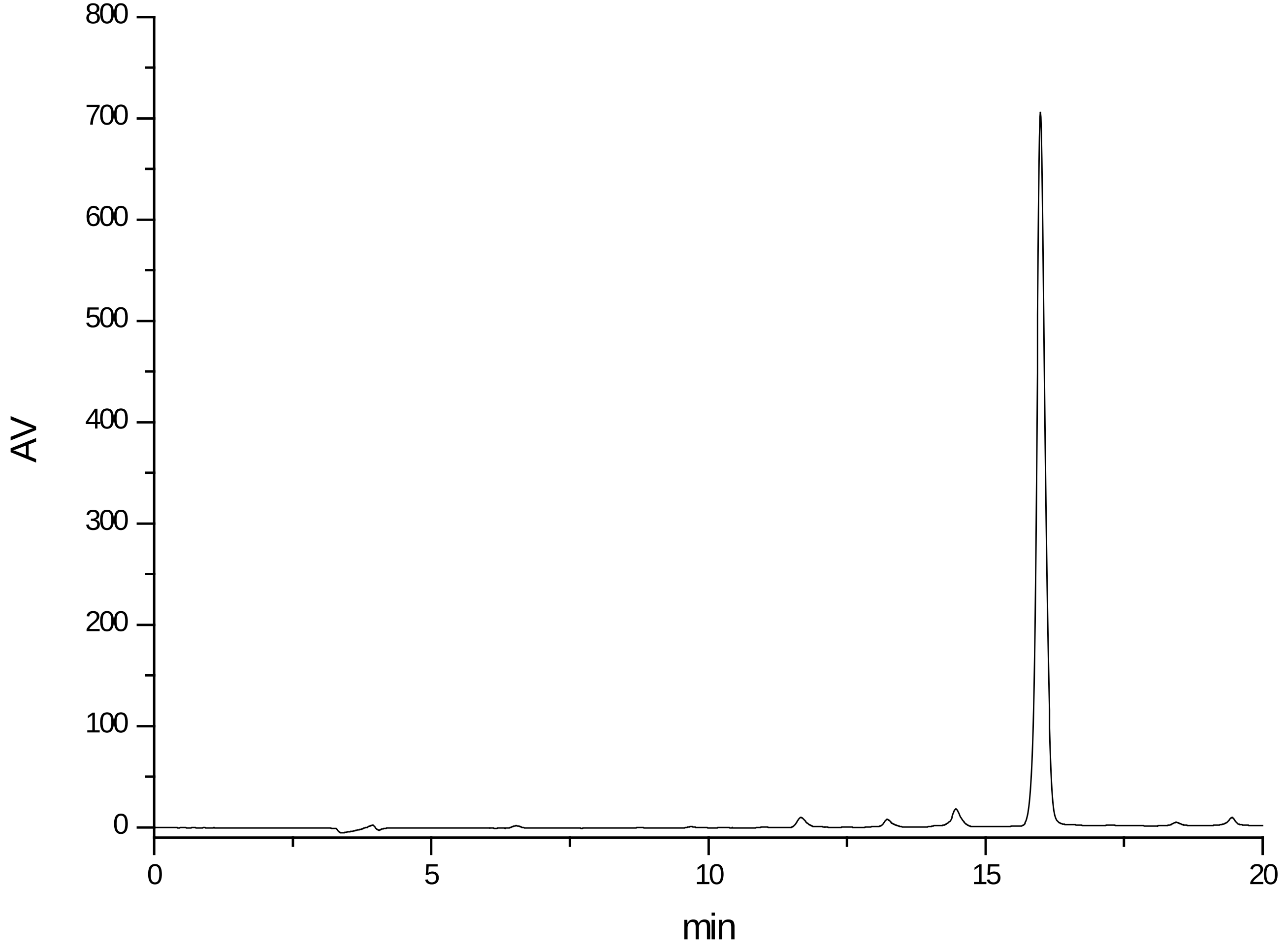

Figure S64. RP-HPLC chromatogram of compound **27**.

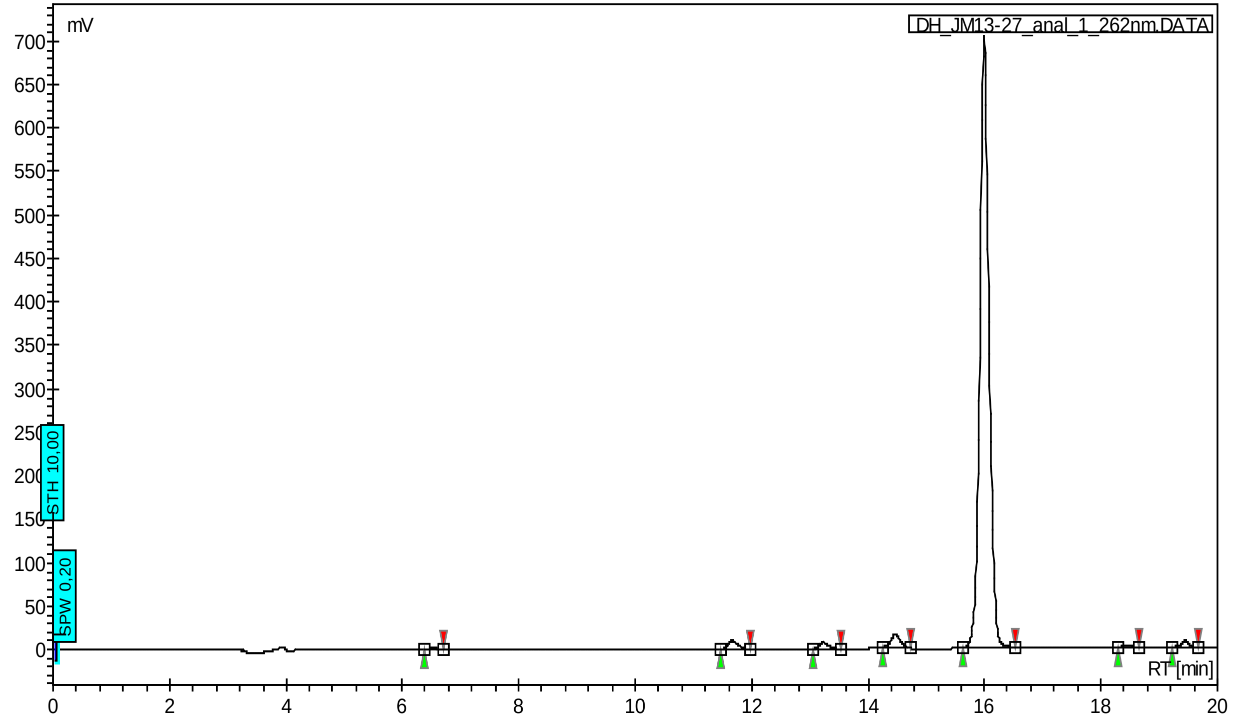

#	Time [Min]	Quantity [% Area]	Height [mV]	Area [mV.Min]	Area % [%]
1	6,533	0,26	2,2	0,4	0,262
2	11,667	1,30	9,5	1,8	1,295
3	13,225	0,90	7,1	1,2	0,898
4	14,467	2,27	16,6	3,1	2,269
5	15,992	94,13	704,9	127,7	94,132
6	18,442	0,33	2,9	0,4	0,329
7	19,442	0,81	7,3	1,1	0,814
Total		100,00	750,4	135,7	100,000

Figure S65. Integration of RP-HPLC chromatogram of compound **27**.
